# Unravelling the Molecular Mechanisms Underlying the Protective Effect of Lactate on the High-Pressure Resistance of *Listeria monocytogenes*

**DOI:** 10.3390/biom11050677

**Published:** 2021-04-30

**Authors:** Cristina Serra-Castelló, Ilario Ferrocino, Anna Jofré, Luca Cocolin, Sara Bover-Cid, Kalliopi Rantsiou

**Affiliations:** 1 IRTA—Food Safety and Functionality Program, Finca Camps i Armet s/n, 17121 Monells, Spain; cristina.serra@irta.cat (C.S.-C.); anna.jofre@irta.cat (A.J.); 2 Department of Agricultural, Forest and Food Science, University of Turin, 10095 Grugliasco, Italy; ilario.ferrocino@unito.it (I.F.); lucasimone.cocolin@unito.it (L.C.)

**Keywords:** *Listeria monocytogenes*, pressurization, HPP, organic acids, piezo-resistance

## Abstract

Formulations with lactate as an antimicrobial and high-pressure processing (HPP) as a lethal treatment are combined strategies used to control *L. monocytogenes* in cooked meat products. Previous studies have shown that when HPP is applied in products with lactate, the inactivation of *L. monocytogenes* is lower than that without lactate. The purpose of the present work was to identify the molecular mechanisms underlying the piezo-protection effect of lactate. Two *L. monocytogenes* strains (CTC1034 and EGDe) were independently inoculated in a cooked ham model medium without and with 2.8% potassium lactate. Samples were pressurized at 400 MPa for 10 min at 10 °C. Samples were subjected to RNA extraction, and a shotgun transcriptome sequencing was performed. The short exposure of *L. monocytogenes* cells to lactate through its inoculation in a cooked ham model with lactate 1h before HPP promoted a shift in the pathogen’s central metabolism, favoring the metabolism of propanediol and ethanolamine together with the synthesis of the B12 cofactor. Moreover, the results suggest an activated methyl cycle that would promote modifications in membrane properties resulting in an enhanced resistance of the pathogen to HPP. This study provides insights on the mechanisms developed by *L. monocytogenes* in response to lactate and/or HPP and sheds light on the understanding of the piezo-protective effect of lactate.

## 1. Introduction

*Listeria monocytogenes* is a facultative anaerobic Gram-positive pathogen that can cause listeriosis, with several outbreaks being associated with ready-to-eat (RTE) products. The risk assessments developed so far indicate that within the RTE meat products, cooked meat products have to be considered of high risk due to the exposure to recontamination with *L. monocytogenes* during the preparation of convenient formats (i.e., sliced/diced and packaged) and due to the potential of *L. monocytogenes* to grow during the refrigerated storage thanks to its psychrotrophic nature [1].

Differences in food safety microbiological criteria regarding *L. monocytogenes* are found between countries, setting from a maximum of 100 CFU/g of *L. monocytogenes* during the shelf-life of the product in EU [2] to the zero-tolerance policy (not detected in 25 g) in USA [3]. In this regard, control measures can be implemented by food manufacturers to comply with the legislation by minimizing the prevalence of the pathogen as well as by limiting its growth in contaminated products.

Among all the available control strategies, high pressure processing (HPP) is an emergent non-thermal technology widely applied in the meat industry. HPP is often used as a post-lethality treatment (PLT) with the aim of reducing microbial loads in foods that have been exposed to microbial recontamination before their commercialization, i.e., during slicing and packaging operations [4]. Another control measure frequently used by the meat industry to prevent the growth of *L. monocytogenes* is the use of antimicrobial agents (AMA), especially organic acids and/or their salts [5]. It is known that organic acids in a medium exist in equilibrium between the undissociated and dissociated state, the former being able to cross the cell membrane entering into the cell, where it dissociates liberating anions [6]. The presence of an increased amount of lactate anions inside the cell increases the osmotic pressure and affects the functioning of the cell metabolism, thus resulting in an impaired bacterial growth. 

In some cases, the combined application of HPP and organic acid salts is chosen by the food industry in order to comply with the highest level of control of *L. monocytogenes* requested in Alternative 1 (combination of a PLT and an AMA) under the requirements of zero tolerance policy of USA [3]. According to the hurdle technology concept described by Leistner [7], the intelligent combination of hurdles (as sub-lethal stresses) leads to an increased effectiveness in controlling *L. monocytogenes* survival/growth. However, cross-protection of a sublethal stress against subsequent treatments can also occur, damaging cells without killing them [8]. Few studies have been conducted dealing with the effect of combination of strategies (i.e., HPP and organic acids) on *L. monocytogenes* in meat products [4,9]. Interestingly, Serra-Castelló et al. [10] showed that the HPP inactivation of three *L. monocytogenes* strains (CTC1034, CTC1011 and Scott A) in cooked ham formulated with potassium lactate was lower than in cooked ham without this antimicrobial. This piezo-protective effect was quantified showing it was strain and lactate dose-dependent. Additionally, in cooked meat products, *L. monocytogenes* surviving HPP was found to grow at higher rate compared to non-pressurized *L. monocytogenes* during the storage of the products [11], such piezo-stimulation effect was enhanced in products formulated with lactate [11].

The present study aimed to investigate by means of transcriptomics the molecular mechanisms underlying the piezo-protective effect exerted by lactate on *L. monocytogenes* HPP inactivation in a cooked ham model medium.

## 2. Material and Methods

### 2.1. Cooked Ham Model Medium Formulation and Characterization

Cooked ham model medium (CHMM) was prepared with Brain Heart Infusion (BHI) broth (Beckson Dickinson, Sparks, MD, USA) and the addition of the following ingredients (g/L) usually used in the manufacture of cooked ham from pork meat: sodium chloride, 15.7; dextrose, 5.77; sodium ascorbate, 0.6; and sodium nitrite, 0.1. The medium was sterilized at 121 °C for 20 min. In order to have samples without organic acids (control) and with lactate, two lots of CHMM were prepared: without and with 2.8% (*v/v*) potassium lactate (using HiPure P Plus, Corbion©, Montmeló, Spain, known to have 76–80% *w/w* of potassium lactate).

### 2.2. L. monocytogenes Strains and Pre-Culture Conditions

Strains of *L. monocytogenes* used in the present study included two different serotypes with relevance from the clinical and from the food and food processing environment perspective [12]. The meat isolate CTC1034 (serotype 4b) from the IRTA Food Safety Program’s collection and previously used in studies dealing with the application of HPP in meat products [10,11,13,14] and the *L. monocytogenes* strain EGDe (serotype 1/2a) as a reference strain. For this study, three biological replicates of each strain were prepared from −80 °C stock cultures.

*L. monocytogenes* strains CTC1034 and EGDe were refreshed into 8 mL of BHI broth for 7 h at 37 °C. Afterwards, 1% (*v*/*v*) were consecutively subcultured in 200 mL of fresh BHI at 37 °C for 14 and 24 h, respectively, in order to standardize the strains at the early stationary phase. After incubation, cultures were preserved frozen at −80 °C supplemented with 20% of glycerol until used [15]. 

### 2.3. Preparation of the Samples and HPP

For each biological replicate, cultures of *L. monocytogenes* strains CTC1034 and EGDe were thawed at ambient temperature and centrifuged at 8240× *g* for 7 min at 12 °C. Supernatants were discarded and cell pellets were resuspended in the same volume of CHMM without or with 2.8% of lactate. Cultures were distributed in 4 × 10 cm PA/PE pouches (oxygen permeability of 50 cm^3^/m^2^/24 h and a low water vapor permeability of 2.8 g/m^2^/24 h; Sistemvac, Estudi Graf S.A., Girona, Spain), which were closed by thermosealing. Cultures were kept for 1 h at 10 °C to allow the adaptation of *L. monocytogenes* cells in CHMM medium without and with 2.8% of lactate. Half of the samples were subsequently pressurized at 400 MPa for 10 min using an industrial HPP equipment (Wave 6000; Hiperbaric, Burgos, Spain). The come-up time was 2.50 min and the pressure release time was almost immediate (<2 s). The pressurization fluid was water and the initial temperature was set at 10 °C. After pressurization, samples were kept for 30 min at 10 °C before *L. monocytogenes* enumeration and RNA extraction. Non-pressurized samples were kept at 10 °C until analysis together with the HPP samples.

### 2.4. L. monocytogenes Enumeration and Data Analysis

For each treatment and biological replicate, *L. monocytogenes* concentration was determined by plate colony count method from the appropriate tenfold serial dilution prepared in 0.1% Bacto Peptone (Difco Laboratories, Detroit, MI, USA) with 0.85% NaCl. Samples were spread on CHROMagar^TM^ Listeria (CHROMagar, Paris, France) and incubated at 37 °C for 48 h according to the manufacturer instructions. Chromogenic media for *L. monocytogenes* are known to be able to recover high pressure injured *L. monocytogenes* [16,17]. In any case, plates were further checked after additional 24–48 h to make sure that sub-lethally injured cells had time to recover and form colonies and, thus, minimize the overestimation of the lethal effect of HPP [10,11]. *L. monocytogenes* counts were Log transformed, and the inactivation value in terms of Log reduction was calculated by subtracting from the counts found in non-pressurized cultures (Log N_0_) those of the pressurized cultures (Log N), i.e., LogN_0_ − LogN = Log N_0_/N, both in the control and 2.8%-lactate lots.

### 2.5. Nucleic Acid Extraction and Sequencing

DNA of the samples prepared according to Section 2.3 was extracted from *L. monocytogenes* strain CTC1034 by using 1 mL of an overnight culture of BHI centrifuged at 14,000× *g* for 10 min. The pellet was then used for DNA extraction according to the protocol described in Cocolin et al. [18]. DNA was quantified using the QUBIT DS-HS kit (Thermo Fisher Scientific, Milan, Italy) and it was standardized at 50 ng/μL. Whole genome sequencing (WGS) was performed using NEBNext^®^ library prep Kit according to the manufacturers’ instructions in paired-end (2 × 150 bp) on a NextSeq 550 Illumina system by the Novagene Company (Cambridge, United Kingdom).

For the transcriptomic analysis, *L. monocytogenes* cultures of CTC1034 and EGDe strains were centrifuged at 10,416× *g* for 5 min at 10 °C and pellets corresponding to 3.6 mL of culture were resuspended with 125 µL of RNAlater solution (Invitrogen, Thermo Fisher Scientific, Barcelona, Spain,) and kept at −80 °C. Total RNA was extracted from the pellets using the RNeasy PowerMicrobiome Kit (QIAGEN, Hilden, Germany) following the manufacturers’ instructions, and residual DNA was removed with TURBO DNase (Invitrogen, Thermo Fisher Scientific, Milan, Italy) according to the manufacturers’ instructions. RNA concentrations were quantified by using a Nanodrop Instrument (Spectrophotometer ND-1000, Thermo Fisher Scientific, Milan, Italy). The RNA integrity was verified by agarose gel electrophoresis. The RNA sequencing library preparation and cDNA synthesis were performed using the NEBNext Ultra RNA Library Prep Kit according to the manufacturers’ instructions at Genewiz Inc. (Leipzig, Germany). The transcriptome was studied for all the samples from the experiment and sequencing was carried out on a NextSeq 550 Sequencer yielding 150 bp paired-end reads.

### 2.6. Bioinformatics and Data Analysis

WGS of *L. monocytogenes* strains CTC1034 led to 5,484,770 paired-end reads. Low-quality bases (Phred score < 20) were trimmed, and reads shorter than 60 bp were discarded using the SolexaQA++software v3.1.7.1 and PRINSEQ v0.20.4, respectively [19,20]. Reads were assembled using SPAdes v3.14.1 [21]; genes were annotated with Prokka v 1.14.5 [22] and used to build the reference database. A draft genome of *L. monocytogenes* EGDe (NC_003210.1) was downloaded from NCBI (BioProject: PRJNA61583), and genes were annotated with Prokka. The pangenome calculation and phylogenetic analysis of *L. monocytogenes* strains were obtained by Roary v. 3.11.2 [23].

In order to investigate the molecular background that could explain the observed differences in the inactivation between the two *L. monocytogenes* strains as well as the piezo-protective effect of lactate, a transcriptomic approach was implemented. Total RNA was extracted, sequenced, and compared between *L. monocytogenes* cultures shortly exposed to (i) CHMM (control without HPP), (ii) CHMM supplemented with lactate (without HPP), (iii) CHMM and subjected to HPP, and (iv) CHMM supplemented with lactate and subjected to HPP.

Raw reads were quality filtered by SolexaQA++ software and PRINSEQ (Phred score < 20, <60 bp). Reads were aligned against the respective build database by using Bowtie2 in end-to-end, sensitive mode according to the strain used. The number of reads mapped to each gene (.*sam* files) were then used for KEGG functional analysis using MEGAN6 software [24]. Data normalization and determination of differentially abundant KEGG genes, among the studied conditions (lactate and HPP, alone, or in combination) or strains, were conducted using the Bioconductor DESeq2 package [25] in the statistical environment R [26] with default parameters. The statistical significance (*p*-values) was adjusted for multiple testing using the Benjamini–Hochberg procedure, which assesses the false discovery rate (FDR) by using the DESeq2 package.

Gene set enrichment for pathway analysis was then performed on KEGG orthologs table imported in the *GAGE* Bioconductor package [27] to identify biological pathways overrepresented or underrepresented between sample without lactate and without HPP treatment against the other combination.

### 2.7. Availability of Data and Material

WGS and Metatranscriptomic raw sequence reads were deposited at the Sequence Read Archive of the National Center for Biotechnology Information (Bioproject accession number: PRJNA692371 and PRJNA692360, for *L. monocytogenes* CTC1034 and EGDe, respectively).

### 2.8. Fatty Acid Profile of L. monocytogenes

For the strain CTC1034 the fatty acid profile was analyzed to confirm potential changes in the membrane composition due to exposure to lactate and/or HPP. For this, samples of *L. monocytogenes* CTC1034 were centrifuged at 10,416× *g* for 6 min at 10 °C. Supernatant was discarded and pellets were resuspended in 1 mL of purified water. Cells were disrupted with 0.5 g of glass beads in a mixer mill (Mixer Mill MM200, Retsch, Llanera, Spain) for 5 min at 30 Hz, centrifuged and supernatant was discarded. Pellets were frozen at −20 °C for 2 h before being freeze dried (Lyomicron LM-181004, Coolvacuum, Granollers, Spain). Methyl esters of fatty acids (FAME) were obtained by methylation described by Castro-Gómez et al. [28], using tritridecanoine as an internal standard. FAME analysis was carried out on an Autosystem chromatograph (Perkin Elmer, Beaconsfield, UK) fitted with a VF-23ms, fused silica capillary column (30 m × 0.25 mm i.d. × 0.25 μm film thickness, Varian, Middelburg, The Netherlands) and FID, according to Calvo et al. [29]. The statistical difference of the results among conditions was assessed through the MANOVA test.

## 3. Results and Discussion

### 3.1. Inactivation of L. monocytogenes by HPP

**Figure 1:**
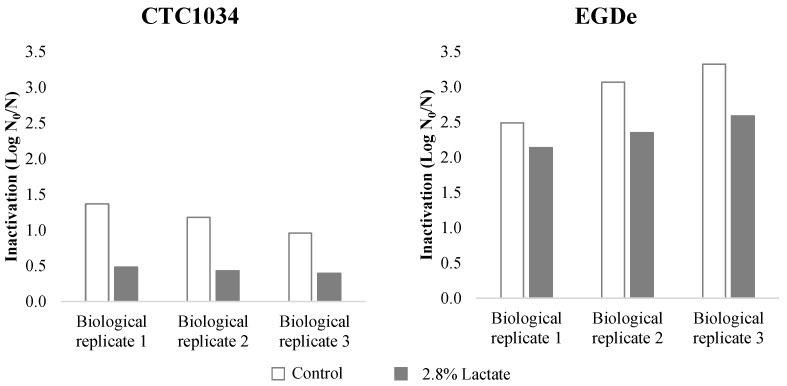
Inactivation (Log N_0_/N) of each biological replicate of the *L. monocytogenes* CTC1034 and EGDe strains observed after HPP (400 MPa for 10 min) in cooked ham model medium without (control) and with 2.8% (*v/v*) potassium lactate.

Inactivation of *L. monocytogenes* strains CTC1034 and EGDe submitted to HPP at 400 MPa for 10 min in the CHMM resembling the composition of a cooked ham, with or without potassium lactate, is shown in Figure 1. The results show that the application of HPP in a medium without lactate inactivated CTC1034 and EGDe strains by an average reduction of 1.17 ± 0.20 and 2.96 ± 0.43 Log units, respectively. Thus, the strain CTC1034 was significantly (*p* < 0.05) more resistant to HPP than EGDe. In the presence of lactate in the CHMM, HPP resulted in a lower inactivation of the strains, recording 0.44 ± 0.04 and 2.36 ± 0.22 Log reduction for CTC1034 and EGDe, respectively. In particular, for the CTC1034 strain, the lethal effect of HPP was lower (*p* < 0.05) in the presence of lactate, corroborating the piezo-protective effect of this antimicrobial on *L. monocytogenes* inactivation as previously shown for this and other strains inoculated in different types of meat products [9,10,30,31].

### 3.2. Analysis of RNA-Seq Results. KEGG Annotation Classification and Pathway Enrichment Analysis of the DEGs

#### 3.2.1. Comparison of *L. monocytogenes* CTC1034 and EGDe Genomes

WGS sequencing of *L. monocytogenes* CTC1034 showed a total of 19 contigs that provide a total genome length of 2,943,406 bp with an average GC content of 38.05%. Sequencing revealed the presence of 2958 CDS, 1 tmRNA and 61 tRNA encoding genes.

The comparison of *L. monocytogenes* genomes of CTC1034 and EGDe strains showed the presence of 2967 core genes including 394 genes encoding hypothetical proteins. Only 77 genes were absent or present in one *L. monocytogenes* strain compared to the other, 35 genes being found in CTC1034 but not in EGDe and 42 being found in EGDe but not in CTC1034. Most of the 35 genes found in CTC1034, but not in EGDe, were related to transcription factors, while the major fraction of genes found in EGDe were involved in protein export and transcription factors. As transcription factors regulate gene expression, a greater abundance in the CTC1034 could be related to the major resistance to HPP stress this strain has shown [32].

#### 3.2.2. Whole Transcriptome Analysis

For the transcriptomic analysis involving both *L. monocytogenes* strains, a total of 152.43 Gbp of clean reads were obtained. For each sample, approximately 6.62 Gbp of reads were found (Supplementary Table S1). The KEGG analysis assigned 864 genes to 24 KEGG pathways.

**Figure 2:**
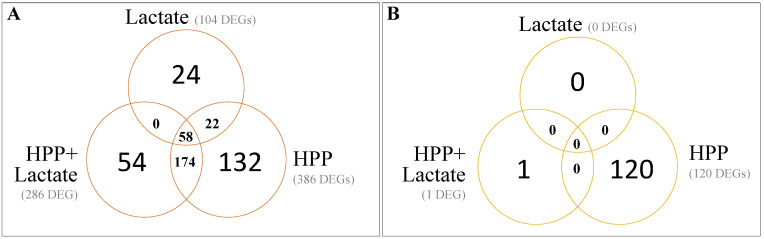
Venn diagrams of differentially expressed genes (DEGs) of *L. monocytogenes* strains CTC1034 (**A**) and EGDe (**B**) due to the exposure of cells to lactate, the application of the HPP (400 MPa for 10 min) and the application of both stresses compared to control conditions (exposed to CHMM without lactate).

Results from the statistical analysis of the KEGG genes obtained with the transcriptomic analysis revealed that the number of differentially expressed genes (DEGs) found in the pairwise comparisons between all the condition combinations studied (effect of lactate, effect of HPP and effect of both factors) was strain-dependent (Figure 2; Supplementary Tables S2–S10).

In this framework, the stress induced by the exposure of *L. monocytogenes* cultures to CHMM with lactate compared to those exposed to CHMM without the antimicrobial resulted in a different response depending on *L. monocytogenes* strain. While the presence of lactate in the CHMM resulted in 104 DEGs in CTC1034, no DEGs were found in EGDe (Figure 2; Supplementary Table S3). A similar pattern was obtained when analyzing the effect of the application of both stresses, lactate and HPP, on *L. monocytogenes* compared to control conditions, resulting in 286 DEGs for the CTC1034 and only 1 DEGs for the EGDe strain (Figure 2; Supplementary Tables S6 and S10). Therefore, these results suggest that the response to stress is highly dependent on the particularities of the *L. monocytogenes* strain. In the study of the transcriptional response of two *L. monocytogenes* strains due to exposure to organic acids (lactate and diacetate) reported by Stasiewicz et al. [33], large differences on the number of transcribed genes were found and only a minor fraction of the differentially transcribed genes were shared between the two strains.

Additionally, it was interesting to observe that DEGs found for EDGe in the pairwise comparison of pressurized samples with and without the presence of lactate (Supplementary Table S8) were the same or involved in the same metabolic pathways as those DEGs found in non-pressurized cultures of CTC1034 in response to lactate stress (Supplementary Table S3). The different pairwise comparisons between the stressing conditions involving lactate also support this hypothesis (Supplementary Tables S4, S5, S8 and S9). These results would lead to the hypothesis that both *L. monocytogenes* strains employ similar molecular mechanisms in response to the lactate stress, although they seem to be activated in a different magnitude and/or time frame.

On the other hand, the application of the HPP resulted in 386 and 120 DEGs for the CTC1034 and EGDe strains, respectively, when compared to control conditions, i.e., *L. monocytogenes* cultures exposed to CHMM without lactate (Figure 2; Supplementary Tables S2 and S7).

The pathway enrichment analysis (performed by GAGE) of the KEGG genes of CTC1034 strains showed an enrichment of several pathways in CHMM subjected to HPP (with and without lactate) compared with the control CHMM (without HPP nor lactate), including Flagellar assembly (ko02040), Fructose and mannose metabolism (ko00051), Phosphotransferase system (ko02060), Biosynthesis of amino acids (ko01230) and Phenylalanine, and tyrosine and tryptophan biosynthesis (ko00400). Moreover, an enrichment of the flagellar assembly (ko02040) and a reduction in glycolysis/gluconeogenesis (ko00010) in CHMM supplemented with lactate without HPP was observed when compared with CHMM. Regarding EGDe, an enrichment in cysteine and methionine metabolism (ko00270), peptidoglycan biosynthesis (ko00550), fatty acid metabolism (ko01212), biosynthesis of amino acids (ko01230) and citrate cycle (ko00020), and a downregulation of the flagellar assembly (ko02040) and phosphotransferase system (PTS) (ko02060) were observed in CHMM subjected to HPP if compared with non-pressurized CHMM (data not shown).

#### 3.2.3. Effect of Lactate Exposure on *L. monocytogenes*

**Figure 3:**
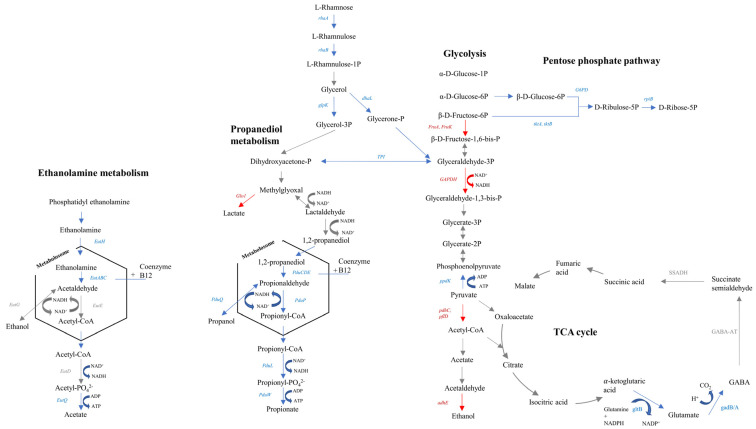
Predicted carbon flux in *L. monocytogenes* CTC1034 and EGDe when exposed to lactate. Blue, red, and grey arrows and text indicate genes that were upregulated, downregulated, or were not differentially expressed, respectively. Genes and proteins: *EutH*, ethanolamine transporter; *EutA*, ethanolamine transporter protein EutA; *EutB*, ethanolamine ammonia-lyase large subunit; *EutC*, ethanolamine ammonia-lyase small subunit; *EutG*, alcohol dehydrogenase; *EutE*, aldehyde dehydrogenase; *EutD*, phosphotransacetylase; *EutQ*, ethanolamine utilization protein EutQ; *Glo1*, lactoylglutathione lyase; *PduC*, propanediol dehydratase large subunit; *PduD*, propanediol dehydratase medium subunit; *PduE*, propanediol dehydratase small subunit; *PduP*, propionaldehyde dehydrogenase; *PduQ*, 1-propanol dehydrogenase; *PduL*, phosphate propanoyltransferase; *PduW*, propionate kinase; *TPI*, triosephosphate isomerase; *FruA*, fructose PTS system EIIBC; *FruK*, 1-phosphofructokinase; *GAPDH*, glyceraldehyde 3-phosphate dehydrogenase; *ppdK*, pyruvate orthophosphate dikinase; *pdhC*, pyruvate dehydrogenase E2 component; *pflD*, formate C-acetyltransferase; *adhE*, acetaldehyde dehydrogenase/alcohol dehydrogenase; *tktA*, *tktB*, transkelotase; *G6PD*, glucose-6-phosphate 1-dehydrogenase; *RpiB*, ribose 5-phosphate isomerase B; *DhaL*, phosphoenolpyruvate-glycerone phosphotransferase subunit DhaL; *GlpK*, glycerol kinase; *RhaB*, rhamnulokinase; *RhaA*, L-rhamnose isomerase; *gltB*, glutamate synthase; *gadB/A*, glutamate descarboxylase; *GABA-AT*, GABA aminotransferase; *SSADH*, succinate semialdehyde dehydrogenase

Some studies support that in order to counteract the intracellular osmotic pressure caused by an increased amount of lactate, bacteria (i) reduce intracellular pools of anions and (ii) shift the flux in the central carbon metabolism [34]. The results from the present transcriptomic analysis reveal that *L. monocytogenes* could use both strategies to overcome the stress suffered by its exposure to lactate. Regarding the possible effect of lactate on the central carbon metabolism of the pathogen, the results of the present study show that genes involved in the pentose phosphate pathway coupled with oxidative reactions to produce reducing equivalents (*rpiB*, *tktA*, *tktB*, *G6PD*) were upregulated. Additionally, a downshift was observed in the conversion of pyruvate to acetyl-CoA and ethanol, as indicated by the downregulation of genes such as *pdhC, plfD,* and *adhE*. In line with the output of the pathway enrichment analysis described above, these transcriptomic results suggest that in presence of lactate, *L. monocytogenes* redistributed its metabolic carbon flux from the glycolytic pathway to oxidative reactions producing reducing equivalents (Figure 3).

Genes of other metabolic pathways that are source of reducing equivalents were also upregulated (Figure 3). In this framework, genes involved in the synthesis of cobalamin and corrinoid cofactors and B12 cofactor (adenosylcobalamin) (*CbiK-CbiX, CbiL, CobI, CbiH, CobJ, CbiF, CobM, CbiD, CbiT, CbiC, CobH, CbiA, CobB, CbiB, CobC, CobD, CobU, CobS, CobV,* and *EutT*), which consist of reductive reactions, were also found to be upregulated (Supplementary Tables S3 and S8). In addition, the higher expression of genes related to the cobalamin and corrinoid pathways is coordinated with the upregulation of the genes involved in the 1,2 propanediol (*PduC*, *PduD*, *PduE*, *PduP*, *PduQ*, *PduL PduW*) and ethanolamine metabolism (*EutH*, *EutA*, *EutB*, *EutC*, *EutQ, EutN, EutJ, EutT, EutL*) found in the presence of lactate (Supplementary Tables S3 and S8), since both pathways are regulated by the cofactor B12 riboswitch in *L. monocytogenes,* the synthesis of the cofactor B12 being required for the metabolism of these pathways [35]. Such coordination is biologically relevant since the B12 cofactor is required in the catabolic pathways of ethanolamine and propanediol degradation. Moreover, genes involved in the catabolism of rhamnose (*rhaA*, *rhaB*, *dhal* and *glpK*) were upregulated, suggesting that it can be used as a carbon source for the 1,2 propanediol pathway [36]. The use of 1,2 propanediol and ethanolamine as a carbon source has been reported to provide a competitive advantage to *L. monocytogenes* under diverse conditions such as when growing in vacuum-packaged smoked salmon [37] or when co-cultured with other bacteria [38]. In the present study the role of 1,2 propanediol and ethanolamine metabolism in the piezo-protective effect of lactate on *L. monocytogenes* could not be directly elucidated, but they are important metabolites that provide a fitness advantage to *L. monocytogenes* [39].

Together with lactate anions, protons are also accumulated inside the cell, with the consequent disruption of bacterial transmembrane potential. In this framework, one of the strategies frequently used by bacteria to restore intracellular pH homeostasis and/or maintain transmembrane potential is the metabolism of glutamate [40,41]. The intracellular decarboxylation of glutamate by a glutamate decarboxylase enzyme to form aminobutyric acid (GABA) results in the consumption of one proton, contributing to restore the intracellular pH [42]. The upregulation of genes involved in the metabolism of glutamate (*gadAB*, *gltBD)* pointed out that *L. monocytogenes* could use this strategy to restore intracellular pH homeostasis disturbed when exposed to lactate (Figure 3; Supplementary Tables S3 and S8).

The enrichment of flagellar assembly pathways and in detail of flagellar genes (*FlhA*, *FlhF*, *FliC*, *FliE*, *FliF*, *FliG*, *FliH*, *FliI*, *FliR*, *FliP*, *FlgB*, *FlgC*, *FlgD*, *FlgE*, *FlgG*, *FlgK,* and *FlgL*) found in the presence of lactate (Supplementary Tables S3 and S8) could indicate that the electrochemical potential of protons across the cytoplasmic membrane could also contribute to fuel the flagellar motor of the pathogen [43] and/or that the unfavorable environment faced by *L. monocytogenes* would promote the pathogen to elicit the chemotactic response and to move to a more favorable environment [44].

The activation of all the strategies to counteract the osmotic pressure and membrane potential changes due to lactate would result in less efficient pathways for ATP production and in a higher energy expenditure, leading to the limitation of growth in the presence of lactate [45,46,47]. A decrease of metabolic energy generation due to the increase in external lactate concentration was described in *Streptococcus cremoris* [48].

**Figure 4:**
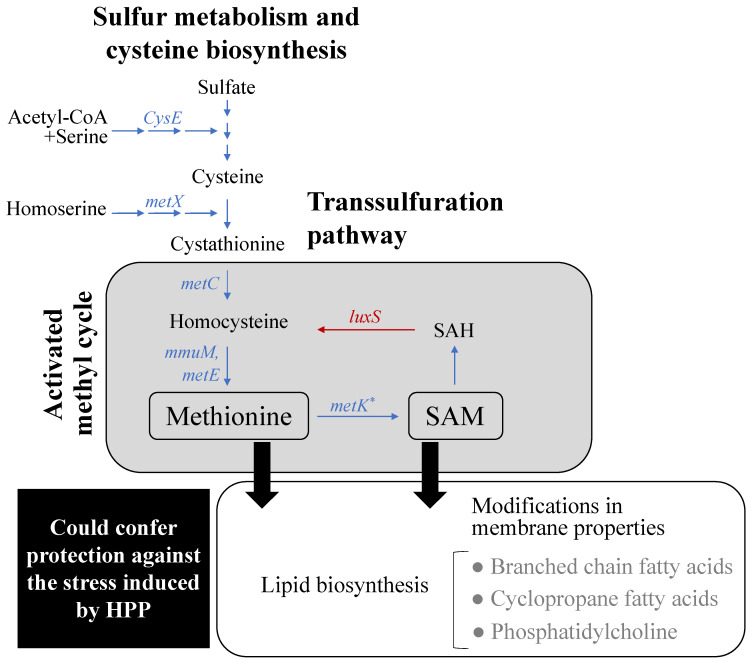
Predicted activation of the methyl cycle in *L. monocytogenes* CTC1034 and EGDe strains when exposed to lactate and its potential role on the piezo-protective effect exerted by lactate on *L. monocytogenes* stress induced by HPP. Blue and red arrows and text indicate genes that were upregulated and downregulated, respectively. Genes and proteins: *CysE*, serine O-acetyltransferase; *metX*, homoserine O-acetyltransferase; *metC*, cysteine-S-conjugate beta-lyase; *mmuM* and *metE*, homocysteine S-methyltransferases; *metK*, S-adenosylmethionine synthetase; *luxS*, S-ribosylhomocysteine lyase; *SAM*, S-adenosyl-methionine; *SAH*, S-adenosyl-homocysteine.

In addition to the up/downregulation of molecular mechanisms involved in restoring osmotic pressure and membrane potential, it is worth to highlight that in the presence of lactate, *L. monocytogenes* specifically upregulated genes involved in the methionine synthesis (Figure 4), in particular a higher expression of the methyltransferases *mmuM* in CTC1034 (Supplementary Table S3) and *MetE* in pressurized EGDe (Supplementary Table S8) was found. Both enzymes are responsible for converting homocysteine to methionine, thus suggesting that in the presence of lactate *L. monocytogenes* promoted the oxidation of homocysteine to methionine, avoiding the accumulation of the toxic metabolite homocysteine and increasing the amount of intracellular methionine. In accordance with this, genes associated with the sulfur metabolism (*metC*, *metX*, *cysE* or *cysO*) involved in the methionine synthesis were also found to be upregulated by the exposure of *L. monocytogenes* to lactate (Figure 4; Supplementary Tables S3 and S8). In previous studies dealing with the transcriptome analysis of *L. monocytogenes* cells exposed to lactate, the upregulation of the methionine biosynthesis was not reported [33,49]. However, in those experiments *L. monocytogenes* was exposed to lactate for a much longer time, i.e., 8 h at 7 °C and 48 h at 15 °C, than the exposure time used in the present study (<2 h at 10 °C). It can be hypothesized that the upregulation of the methionine synthesis would only occur in the early exposure of the pathogen to lactate as a first step of the overall mechanism to overcome the stress suffered by the presence of lactate. In addition to the time-related factor, other potential reasons leading to different results include the pathogen strains, the concentration and the type of salt (sodium vs. potassium), and the incubation temperature or the matrix composition (culture medium) used for the experiment.

Among all the multiple factors that can determine the expression of genes involved in the methionine synthesis, the observed upregulation of this metabolic pathway by *L. monocytogenes* in the presence of lactate could be relevant in relation to the piezo-resistance mechanisms since another organic acid such as acetate has been shown to specifically inhibit the synthesis of methionine in *Escherichia coli*, favoring the accumulation of the toxic compound homocysteine and consequently limiting or even inhibiting the growth of the pathogen [50]. Moreover, Roe et al. [50] reported that the addition of methionine in the medium containing acetate restores *E. coli* growth to 80% of that observed in medium without acetate, indicating that the inhibition of the methionine biosynthesis is one of the main factors responsible for the growth depletion of *E. coli* cultured in the presence of acetate. Supporting these results, Pinhal et al. [51] reported that the uncoupling effect of acetate or the perturbation of the anion composition of the cell played only a limited role (20%) in the *E. coli* growth depletion, suggesting that other molecular mechanisms, such as the inhibition of the methionine synthesis, could have a more prominent role on the bacterial growth-inhibitory effect.

Methionine can be converted to S-adenosyl-L-methionine (SAM), which represents a methyl group donor for many fundamental cellular processes, such as cellular signaling and epigenetic regulations that promote cellular anabolism and proliferation in bacteria and yeasts [52,53]. Specifically, SAM is involved in the methylation of proteins, RNAs, biotin, polyamines, and lipids [53,54]. In the present study, the *metK* gene responsible for the conversion of methionine to SAM was found to be upregulated in the *L. monocytogenes* CTC1034 strain when it was exposed to lactate, suggesting a higher production of SAM. Moreover, an increased intracellular concentration of methionine was also reported to contribute to the antioxidant defense in bacteria [55], although its role in the piezo-protection remains unknown.

#### 3.2.4. Effect of HPP on *L. monocytogenes*

The transcriptomic analysis revealed that both *L. monocytogenes* strains upregulated genes involved in DNA repair mechanisms such as *RadA*, *phrB*, *uvrB*, *adaB*, and lipid and peptidoglycan biosynthetic pathways (*glmS, murF, murG, murC,* or *fabH*), among others (Supplementary Tables S2 and S7), presumably as a consequence of the stress induced by the application of the HPP to *L. monocytogenes*. In case of flagella assemblage (*FlhA*, *FlhF*, *FliC*, *FliE*, *FliF*, *FliG*, *FliH*, *FliI*, *FliR*, *FliP*, *FlgB*, *FlgC*, *FlgD*, *FlgE*, *FlgG*, *FlgK* and *FlgL*) and chemotaxis (*MotA*, *CheA*, *CheR*, *CheY*, *FliG*, *FliM,* and *FliN/FliY*), an upregulation of genes involved in these pathways was found in CTC1034 (Supplementary Table S2), while a downregulation was observed in EGDe (Supplementary Table S7). These differences could be related to the particularities of each *L. monocytogenes* strain but also to the higher severity of the HPP injury in the EGDe strain compared to CTC1034, leading to a higher inactivation extent (Figure 1). An important parameter influencing motility of *L. monocytogenes* is temperature; *L. monocytogenes* cells are motile at temperatures below 30 °C but not at human body temperature (37 °C) [56]. Additionally, flagella, as cell surface appendices, are considered putative virulence factors. In the current study, the temperature for the experiments could partially explain the upregulation of the flagella genes in CTC1034. In addition to this, we may deduce that these genes would be downregulated when *L. monocytogenes* is under stress (for example desiccation) [57]. It is therefore puzzling that HPP resulted in an upregulation in CTC1034, and at this point we cannot provide a biological explanation. Nevertheless, this observation is particularly relevant since it suggests that cells of *L. monocytogenes* surviving the HPP treatment would be prepared to colonize the human body [58]. On the other hand, HPP was found to downregulate genes involved in the septal ring (*ftsA, ftsW*, *ftsQ, mreB*). These results were in line with those reported by Bowman et al. [59] regarding the response of *L. monocytogenes* pressurized at 400–600 MPa for 5 min in tryptone soy yeast extract (TSYE) broth.

As a response to HPP, *L. monocytogenes* CTC1034 and EGDe upregulated genes involved in the methionine biosynthesis (*luxS, mmuM, msrB*), suggesting an enhanced methionine production/availability (Supplementary Tables S2 and S8), which also agrees with the enrichment gene analysis for EGDe (see Section 3.2.2). The upregulation of these genes pointed out that, as stated due to the exposure to lactate (Section 3.2.3), the application of HPP would result in a higher generation of SAM in *L. monocytogenes*, which could affect cellular processes throughout its role in the methyl cycle [60]. These results are in accordance with those reported by Bravim et al. [61], where it was found an upregulation of the sulfur metabolism genes involved in the activation of the methionine biosynthesis when *Saccharomyces cerevisiae* was submitted to an HPP of 50 MPa for 30 min.

Considering the metabolic pathways in which methionine and SAM are involved, methionine could increase *L. monocytogenes* resistance to HPP for its role as an endogenous antioxidant in cells [62] and for its involvement in lipid biosynthesis [63]. Since the HPP affects the bacterial membrane properties [64,65,66], the involvement of methionine in lipid biosynthesis could play a role in the HPP resistance (Figure 4). In this regard, according to the results of the fatty acid profile of *L. monocytogenes* CTC1034 (Table 1) compared with the control conditions when the pathogen was exposed to lactate and/or HPP stresses, cells tended to increase, although not significantly, the level of total branched-chain fatty acids (BCFAs, specifically *iso* and/or *anteiso* conformations of C13, C14, C15, C16, C17). This finding agrees with the fact that in *L. monocytogenes* BCFAs contribute to membrane fluidity and resistance against environmental stresses [67].

SAM was reported to be required for the synthesis of phosphatidylcholine from phosphatidylethanolamine [68] and to have a role in transferring a methylene group to mature phospholipids that lead to the formation of cyclopropane fatty acids (CFAs), a major component of the phospholipids of the bacterial membrane bilayers [69]. A higher proportion of CFAs in the membrane bilayer of *Escherichia coli* has been shown to increase the resistance of the pathogen submitted to HPP of 500 MPa for 5 to 30 min [70]. Since the pressure resistance of *E. coli* is reported to be related to an altered membrane functionality and with the resistance of this pathogen to oxidative stress [71], it was suggested by Chen et al. [70] that CFAs could contribute to pressure resistance by increasing the resistance of membrane lipids to the oxidative stress derived from the application of the HPP. Therefore, the results of the present study point out that the exposure of *L. monocytogenes* cells to lactate prior the HPP would upregulate the methionine biosynthesis pathway, thus contributing to enhance the resistance against HPP by changes in the lipidic membrane functionality.

The higher expression of the methionine biosynthesis pathway by *L. monocytogenes* exposed to lactate and the inhibition of the biosynthesis of this amino acid by acetate reported for *E. coli* [50] could be the reason why the piezo-protective effect on *L. monocytogenes* treated at 400 MPa for 10 min was only seen for cooked ham formulated with lactate and not with diacetate [10]. Further studies regarding *L. monocytogenes* membrane functionality (membrane composition, fluidity, and integrity) as a function of the exposure of lactate and the application of the HPP need to be conducted to experimentally to confirm the role of the membrane properties on the piezo-protective effect exerted by lactate on HPP inactivation of *L. monocytogenes*.

The increased expression of the methionine pathway by *L. monocytogenes* CTC1034 under HPP stress could explain, at least partially, the piezo-stimulation effect (which was enhanced by the presence of lactate) in the growth rate of *L. monocytogenes* CTC1034 cells surviving a HPP at 600 MPa for 3 min observed by Bover-Cid et al. [11]. Since methionine is a key amino acid involved in enabling cell proliferation as precursor of anabolic pathways [72], the upregulation of the methionine biosynthesis due to lactate and HPP stresses could help *L. monocytogenes* cells to repair cellular membrane and enhance their subsequent proliferation. Nevertheless, further studies should be conducted to complement and support this.

## 4. Conclusions

New insights are provided regarding the molecular mechanisms underlying the protective effect of lactate on *L. monocytogenes* submitted to HPP. The short exposure of *L. monocytogenes* cells to lactate promoted a shift in the pathogen’s central metabolism, favoring the propanediol and ethanolamine pathways together with the synthesis of the B12 cofactor, which could confer a competitive advantage for *L. monocytogenes* to overcome the stress suffered by HPP. Changes to the central metabolism, together with responses involving the modification of the intracellular pool of anions or pH homeostasis such as glutamate metabolism or enrichment of flagellar assembly pathways could constitute mechanisms responsible for the piezo-protective effect of lactate. The upregulation of the methionine synthesis pathway after exposure to lactate could also be relevant in relation to the piezo-resistance mechanisms through changes in the properties of the cytoplasmic membrane and its ability to cope with pressure stress. Further studies regarding the *L. monocytogenes* membrane functionality (membrane composition, fluidity, and integrity) as a function of the exposure of lactate and the application of the HPP need to be conducted to experimentally confirm the role of the membrane properties on the piezo-protection and piezo-stimulation effect exerted by lactate on HPP inactivation of *L. monocytogenes*.

## Figures and Tables

**Table 1:** Fatty acid profile (mean % ± standard deviation) of *L. monocytogenes* CTC1034 after exposure of cells to lactate, after the application of the HPP (400 MPa for 10 min), and after the application of both stresses compared to control conditions (exposed to CHMM without lactate).

Fatty Acid	Condition			
	Control	Lactate	HPP	Lactate + HPP
C10:0	0.02 ± 0.03	0.09 ± 0.12	0.03 ± 0.04	0.08 ± 0.01
C12:0	1.03 ± 0.32	0.87 ± 0.07	0.73 ± 0.08	0.77 ± 0.03
C13 *iso*	0.12 ± 0.01	0.12 ± 0.10	0.06 ± 0.00	0.06 ± 0.03
C13 *anteiso*	0.23 ± 0.08	0.27 ± 0.06	0.26 ± 0.07	0.26 ± 0.06
C14 *iso*	1.26 ± 0.01	1.22 ± 0.05	1.22 ± 0.15	1.21 ± 0.16
C14	4.59 ± 0.40	3.99 ± 0.61	4.04 ± 0.77	3.99 ± 0.89
C15 *iso*	14.05 ± 0.83	15.45 ± 0.09	14.31 ± 0.28	14.59 ± 0.18
C15 *anteiso*	39.72 ± 3.06	41.78 ± 1.28	41.24 ± 0.31	41.14 ± 0.45
C15	0.42 ± 0.18	0.49 ± 0.06	0.51 ± 0.05	0.51 ± 0.12
C16 *iso*	3.13 ± 0.03	3.26 ± 0.35	3.60 ± 0.58	3.40 ± 0.21
C16	5.90 ± 2.51	4.17 ± 0.38	4.29 ± 0.60	4.03 ± 0.15
C16:1	2.62 ± 0.00	2.44 ± 1.19	2.79 ± 0.86	2.63 ± 0.58
C17 *iso*	4.63 ± 0.35	4.82 ± 0.05	5.23 ± 0.07	5.06 ± 0.20
C17 *anteiso*	16.75 ± 1.39	17.28 ± 0.60	17.91 ± 0.73	18.10 ± 0.53
C18	1.72 ± 0.71	1.11 ± 0.11	1.12 ± 0.13	1.38 ± 0.05
C18:1 cis9	3.31 ± 1.28	2.33 ± 0.10	2.40 ± 0.21	2.39 ± 0.17
C18:1 cis11	0.02 ± 0.03	0.04 ± 0.05	0.01 ± 0.02	0.00 ± 0.00
C19:0	0.11 ± 0.04	0.08 ± 0.01	0.09 ± 0.01	0.26 ± 0.10
C18:2	0.36 ± 0.17	0.20 ± 0.09	0.17 ± 0.14	0.16 ± 0.10
BCFA ^a^	79.89 ± 5.67	84.20 ± 1.57	83.82 ± 0.59	83.82 ± 0.24
*iso* BCFA	23.19 ± 1.15	24.88 ± 0.36	24.41 ± 0.52	24.31 ± 0.37
*anteiso* BCFA	56.70 ± 4.53	59.32 ± 1.93	59.41 ± 1.12	59.50 ± 0.13
*iso*/*anteiso*	0.41 ± 0.02	0.42 ± 0.02	0.41 ± 0.02	0.41 ± 0.01
C13 BCFA	0.35 ± 0.07	0.39 ± 0.04	0.32 ± 0.08	0.32 ± 0.02
C15 BCFA	53.77 ± 3.89	57.23 ± 1.37	55.55 ± 0.59	55.73 ± 0.63
C17 BCFA	21.38 ± 1.74	22.10 ± 0.65	23.14 ± 0.66	23.16 ± 0.73
C15 BCFA/C17 BCFA	2.51 ± 0.02	2.59 ± 0.01	2.40 ± 0.05	2.41 ± 0.10
C15 *anteiso*/C17 *anteiso*	2.37 ± 0.01	2.42 ± 0.01	2.31 ± 0.08	2.27 ± 0.09

^a^—Branched-chain fatty acids.

## Data Availability

WGS and Metatranscriptomic raw sequence reads were deposited at the Sequence Read Archive of the National Center for Biotechnology Information (Bioproject accession number: PRJNA692371 and PRJNA692360, for *L. monocytogenes* CTC1034 and EGDe, respectively).

## Supplementary Materials

The following are available online at https://www.mdpi.com/article/10.3390/biom11050677/s1, Table S1: Number of raw and clean reads from the transcriptomic analysis of both *L. monocytogenes* strains CTC1034 and EGDe in CHMM without and with lactate and/or without and with HPP of 400 MPa for 10 min, Table S2: List of KEGG Orthology (KO) genes differentially (FDR < 0.05) expressed in the *L. monocytogenes* strain CTC1034 in samples without lactate pressurized and non-pressurized. Positive Log2 fold change indicates genes more abundant in pressurized samples, Table S3: List of KEGG Orthology (KO) genes differentially (FDR < 0.05) expressed in the *L. monocytogenes* CTC1034 strain in non-pressurized samples without and with lactate. Positive Log2 fold change indicates genes more abundant in samples with lactate, Table S4: List of KEGG Orthology (KO) genes differentially (FDR < 0.05) expressed in the *L. monocytogenes* strain CTC1034 in pressurized samples without and with lactate. Negative Log2 fold change indicates genes less abundant in samples with lactate, Table S5: List of KEGG Orthology (KO) genes differentially (FDR < 0.05) expressed in the *L. monocytogenes* strain CTC1034 in samples with lactate non-pressurized and pressurized. Positive Log2 fold change indicates genes more abundant in pressurized samples, Table S6: List of KEGG Orthology (KO) genes differentially (FDR < 0.05) expressed in CTC1034 *L. monocytogenes* strain throughout the comparison of control samples (non-exposed to lactate and non-pressurized) to samples exposed to lactate and pressurized. Positive Log2 fold change indicates genes more abundant in samples exposed to lactate and pressurized, Table S7: List of KEGG Orthology (KO) genes differentially (FDR < 0.05) expressed in the *L. monocytogenes* strain EGDe in samples without lactate pressurized and non-pressurized. Positive Log2 fold change indicates genes more abundant in pressurized samples, Table S8: List of KEGG Orthology (KO) genes differentially (FDR < 0.05) expressed in the *L. monocytogenes* strain EGDe in pressurized samples without and with lactate. Positive Log2 fold change indicates genes more abundant in samples with lactate, Table S9: List of KEGG Orthology (KO) genes differentially (FDR < 0.05) expressed in the *L. monocytogenes* strain EGDe in samples with lactate non-pressurized and pressurized. Positive Log2 fold change indicates genes more abundant in pressurized samples, Table S10: List of KEGG Orthology (KO) genes differentially (FDR < 0.05) expressed in EGDe *L. monocytogenes* strain throughout the comparison of control samples (non-exposed to lactate and non-pressurized) to samples exposed to lactate and pressurized. Positive Log2 fold change indicates genes more abundant in samples exposed to lactate and pressurized.
