# Supplementary material for: Unravelling the Molecular Mechanisms Underlying the Protective Effect of Lactate on the High-Pressure Resistance of Listeria monocytogenes
Source: Biomolecules. 2021 Apr 30;11(5):677. doi: 10.3390/biom11050677 (PMC8147161; doi:10.3390/biom11050677)
Supplement: Supplementary file 1 [file biomolecules-11-00677-s001.zip › biomolecules-1111984-proof-suppl/supplementary table 1.pdf]

Table S1. Number of raw and clean reads from the transcriptomic analysis of both *L. monocytogenes* strains CTC1034 and EGDe in CHMM without and with lactate and/or without and with HPP of 400 MPa for 10 min.

**Statitistics of the RNAseq**							
Sample ID	strain	Lactate (%)	HPP	N° Raw reads	Mbp	N° of clean reads	Mbp
L01	CTC1034	0	NO	15151946	15.15	7419173	7.42
L02	CTC1034	0	YES	18458119	18.46	8684077	8.68
L03	CTC1034	2.8	NO	15054772	15.05	7309514	7.31
L04	CTC1034	2.8	YES	16937935	16.94	2240700	2.24
L05	CTC1034	0	NO	15463670	15.46	7476954	7.48
L06	CTC1034	0	YES	17855188	17.86	8584056	8.58
L07	CTC1034	2.8	NO	15812961	15.81	7192035	7.19
L08	CTC1034	2.8	YES	18967570	18.97	8894415	8.89
L09	CTC1034	0	NO	12918689	12.92	6129884	6.13
L10	CTC1034	0	YES	14012795	14.01	6361482	6.36
L11	CTC1034	2.8	NO	17901907	17.90	8380534	8.38
L12	CTC1034	2.8	YES	17588988	17.59	8385232	8.39
L13	EGDE	0	NO	15620477	15.62	7182132	7.18
L14	EGDE	0	YES	17601934	17.60	8190118	8.19
L15	EGDE	2.8	NO	16011871	16.01	2689152	2.69
L16	EGDE	2.8	YES	16101546	16.10	7131864	7.13
L18	EGDE	0	YES	16825622	16.83	7758071	7.76
L19	EGDE	2.8	NO	14571795	14.57	6784088	6.78
L20	EGDE	2.8	YES	14885726	14.89	2297371	2.30
L21	EGDE	0	NO	17957598	17.96	5530105	5.53
L22	EGDE	0	YES	18917319	18.92	4902398	4.90
L23	EGDE	2.8	NO	14853183	14.85	6920220	6.92
L24	EGDE	2.8	YES	15657958	15.66	5988036	5.99
