# Supplementary material for: Unravelling the Molecular Mechanisms Underlying the Protective Effect of Lactate on the High-Pressure Resistance of Listeria monocytogenes
Source: Biomolecules. 2021 Apr 30;11(5):677. doi: 10.3390/biom11050677 (PMC8147161; doi:10.3390/biom11050677)
Supplement: Supplementary file 1 [file biomolecules-11-00677-s001.zip › biomolecules-1111984-proof-suppl/supplementary table 10.pdf]

Table S10. List of KEGG Orthology (KO) genes differentially (FDR<0.05) expressed in EGDe *L. monocytogenes* strain throughout the comparison of control samples (non-exposed to lactate and non-pressurized) to samples exposed to lactate and pressurized. Positive Log2 fold change indicate genes more abundant in samples exposed to lactate and pressurized.

Log2 Fold Change	FDR	KEGG annotation at level 1	KEGG annotation at level 2	KEGG pathway	KEGG Orthology (KO) genes
2.164	3.40E-03	Unclassified	Unclassified: metabolism	Enzymes with EC numbers	K01531 - P-type Mg2+ transporter [EC:7.2.2.14], mgtA, mgtB
