# Supplementary material for: Unravelling the Molecular Mechanisms Underlying the Protective Effect of Lactate on the High-Pressure Resistance of Listeria monocytogenes
Source: Biomolecules. 2021 Apr 30;11(5):677. doi: 10.3390/biom11050677 (PMC8147161; doi:10.3390/biom11050677)
Supplement: Supplementary file 1 [file biomolecules-11-00677-s001.zip › biomolecules-1111984-proof-suppl/supplementary table 2.pdf]

Table S2. List of KEGG Orthology (KO) genes differentially (FDR<0.05) expressed in the *L. monocytogenes* strain CTC1034 in samples without lactate pressurized and non-pressurized. Positive Log2 fold change indicate genes more abundant in pressurized samples.

Log2 Fold Change	FDR	KEGG annotation at level 1	KEGG annotation at level 2	KEGG pathway	KEGG Orthology (KO) genes
5.602	3.18E-04	Environmental Information Processing	Signal Transduction	Two-component system	K07646 - two-component system, OmpR family, sensor histidine kinase KdpD [EC:2.7.13.3]
5.346	4.54E-07	Metabolism	Amino Acid Metabolism	Cysteine and methionine metabolism	K07173 - S-ribosylhomocysteine lyase luxS [EC:4.4.1.21]
5.344	9.38E-06	Unclassified	Protein families: genetic information processing	Transcription factors	K13530 - AraC family transcriptional regulator, regulatory protein of adaptative response / methylphosphotriester-DNA alkyltransferase methyltransferase AdaA
5.344	6.74E-05	Unclassified	Unclassified: metabolism	Enzymes with EC numbers	K07047 - N-substituted formamide deformylase nfdA
5.321	2.37E-09	Metabolism	Carbohydrate Metabolism	pyruvate metabolism	K00925 - acetate kinase ackA [EC:2.7.2.1]
5.166	1.07E-10	Metabolism	Carbohydrate Metabolism	Starch and sucrose metabolism	K00690 - sucrose phosphorylase [EC:2.4.1.7]
5.151	1.49E-05	Metabolism	Energy Metabolism	Oxidative phosphorylation	K02113 - F-type H+-transporting ATPase subunit delta atpH [EC:3.6.3.14]
4.971	1.68E-07	Metabolism	Amino Acid Metabolism	Arginine and proline metabolism	K00818 - acetylornithine aminotransferase argD [EC:2.6.1.11]
4.896	2.41E-05	Unclassified	Unclassified: metabolism	Amino acid metabolism	K06997 - PLP dependent protein, yggS, PROSC
4.864	1.03E-05	Metabolism	Amino Acid Metabolism	Histidine metabolism	K01496 - phosphoribosyl-AMP cyclohydrolase hisI [EC:3.5.4.19]
4.850	1.23E-09	Unclassified	Protein families: genetic information processing	Ribosome biogenesis	K06941 - 23S rRNA (adenine2503-C2)-methyltransferase rlmN
4.754	1.64E-07	Metabolism	Carbohydrate Metabolism	Glyoxylate and dicarboxylate metabolism	K00865 - glycerate kinase glxK, garK [EC:2.7.1.31]
4.742	4.24E-11	Metabolism	Metabolism of Cofactors and Vitamins	Porphyrin and chlorophyll metabolism	K00231 - oxygen-dependent protoporphyrinogen oxidase hemY [EC:1.3.3.4]
4.725	7.98E-09	Metabolism	Carbohydrate Metabolism	Pentose phosphate pathway	K01808 - ribose 5-phosphate isomerase B rpiB [EC:5.3.1.6]
4.692	1.89E-05	Genetic Information Processing	Translation	Aminoacyl-tRNA biosynthesis	K04567 - lysyl-tRNA synthetase, class II lysS [EC:6.1.1.6]
4.674	8.47E-09	Metabolism	Metabolism of Terpenoids and Polyketides	Terpenoid backbone biosynthesis	K00938 - phosphomevalonate kinase mvaK2 [EC:2.7.4.2]
4.666	4.58E-12	Metabolism	Amino Acid Metabolism	Arginine and proline metabolism	K10536 - agmatine deiminase aguA [EC:3.5.3.12]
4.575	4.64E-07	Unclassified	Protein families: genetic information processing	Chaperons and folding catalysts	K03686 - molecular chaperone DnaJ
4.571	4.54E-08	Metabolism	Metabolism of Cofactors and Vitamins	One carbon pool by folate	K01934 - 5-formyltetrahydrofolate cyclo-ligase MTHFS [EC:6.3.3.2]
4.564	2.16E-09	Metabolism	Amino acid metabolism	Lysine biosynthesis	K01929 - UDP-N-acetylmuramoyl-tripeptide--D-alanyl-D-alanine ligase MurF

4.501	3.02E-06	Metabolism	Metabolism of Other Amino Acids	D-Glutamine and D-glutamate metabolism	K01924 - UDP-N-acetylmuramate--alanine ligase MurC
4.445	5.00E-07	Unclassified	Unclassified: metabolism	Enzymes with EC numbers	K07107 - acyl-CoA thioester hydrolase ybgC [EC:3.1.2.-]
4.445	3.80E-08	Unclassified	Protein families: genetic information processing	Chaperons and folding catalysts	k03687 - molecular chaperone GrpE
4.379	1.11E-06	Environmental Information Processing	Membrane transport	ABC transporters	K02000 - glycine betaine/proline transport system ATP-binding protein proV [EC:3.6.3.32]
4.368	8.79E-08	Unclassified	Protein families: genetic information processing	Chromosome and associated proteins	K09772 - cell division inhibitor SepF
4.274	8.24E-07	Environmental Information Processing	Membrane transport	ABC transporters	K17320 - putative aldouronate transport system permease protein lplC
4.261	8.47E-09	Environmental Information Processing	Signal Transduction	Two-component system	K11617 - two-component system, NarL family, sensor histidine kinase LiaS [EC:2.7.13.3]
4.255	1.77E-05	Unclassified	Protein families: genetic information processing	Chromosome and associated proteins	K03466 - DNA segregation ATPase ftsK/SpoIIIE, S-DNA-T family
4.197	2.89E-06	Unclassified	Protein families: genetic information processing	Transcription factors	K03402 - transcriptional regulator of arginine metabolism argR, ahrC
4.182	8.90E-07	Unclassified	Protein families: signaling and cellular processes	Transporters	K15738 - ABC transport system ATP-binding/permease protein uup
4.113	3.04E-08	Unclassified	Protein families: genetic information processing	Ribosome biogenesis	K02834 - ribosome-binding factor A rbfA
4.102	1.03E-06	Genetic Information Processing	Translation	ribosome	K02994 - small subunit ribosomal protein S8 rpsH
4.068	1.78E-11	Metabolism	Energy Metabolism	Oxidative phosphorylation	K02111 - F-type H+-transporting ATPase subunit alpha ATPF1A, atpA [EC:3.6.3.14]
4.060	4.87E-09	Metabolism	Energy Metabolism	Oxidative phosphorylation	K02828 - cytochrome aa3-600 menaquinol oxidase subunit III qoxC [EC:1.10.3.12]
4.010	4.29E-10	Metabolism	Amino Acid Metabolism	Arginine and proline metabolism	K00657 - diamine N-acetyltransferase speG, SAT [EC:2.3.1.57]
4.005	3.81E-09	Environmental Information Processing	Membrane transport	ABC transporters	K11705 - iron/zinc/copper transport system permease protein mtsC
3.978	9.60E-08	Unclassified	Protein families: genetic information processing	Ribosome biogenesis	K03790 - [ribosomal protein S5]-alanine N-acetyltransferase rimJ
3.973	1.05E-06	Metabolism	Nucleotide Metabolism	purine metabolism	K00759 - adenine phosphoribosyltransferase apt [EC:2.4.2.7]

3.952	5.36E-10	Organismal Systems	Environmental Adaptation	Plant-pathogen interaction	K02358 - elongation factor Tu, tuf, TUFM
3.917	6.47E-08	Unclassified	Unclassified: metabolism	Amino acid metabolism	K04028 - ethanolamine utilization protein EutN
3.911	3.01E-07	Environmental Information Processing	membrane transport	ABC transporters	K11050 - multidrug/hemolysin transport system ATP-binding protein ABC-2.CYL.A, cylA
3.906	6.27E-09	Unclassified	Protein families: genetic information processing	DNA replication proteins	K03168 - DNA topoisomerase I topA
3.894	8.34E-06	Metabolism	Amino Acid Metabolism	Valine, leucine and isoleucine degradation	K00020 - 3-hydroxyisobutyrate dehydrogenase mmsB [EC:1.1.1.31]
3.889	1.03E-06	Environmental Information Processing	Signal Transduction	Two-component system	K02556 - chemotaxis protein MotA
3.874	1.07E-10	Environmental Information Processing	Membrane transport	ABC transporters	K02013 - iron complex transport system ATP-binding protein ABC.FEV.A [EC:3.6.3.34]
3.866	3.56E-09	Unclassified	Protein families: genetic information processing	Ribosome biogenesis	K03215 - RNA methyltransferase, TrmH family, rumA
3.823	2.03E-10	Unclassified	Unclassified: metabolism	Enzymes with EC numbers	K07305 - peptide-methionine (R)-S-oxide reductase msrB
3.811	6.27E-09	Unclassified	-	-	K09769 - uncharacterized protein
3.809	6.26E-06	Unclassified	Protein families: metabolism	Peptidases and inhibitors	K01271 - Xaa-Pro dipeptidase PepQ
3.809	1.29E-09	Genetic Information Processing	Translation	ribosome	K02931 - large subunit ribosomal protein L5, RP-L5, MRPL5
3.809	1.21E-08	Environmental Information Processing	Membrane transport	ABC transporters	K02036 - phosphate transport system ATP-binding protein pstB [EC:3.6.3.27]
3.808	1.15E-04	Metabolism	Metabolism of Cofactors and Vitamins	Folate biosynthesis	K03638 - molybdopterin adenylyltransferase moaB [EC:2.7.7.75]
3.754	1.10E-09	Genetic Information Processing	Translation	ribosome	K02887 - large subunit ribosomal protein L20, RP-L20, MRPL20, rplT
3.745	6.23E-11	Genetic Information Processing	Replication and Repair	Nucleotide excision repair	K03702 - excinuclease ABC subunit B uvrB
3.739	9.78E-06	Metabolism	Metabolism of Terpenoids and Polyketides	Terpenoid backbone biosynthesis	K03527 - 4-hydroxy-3-methylbut-2-enyl diphosphate reductase ispH, lytB [EC:1.17.1.2]
3.733	4.14E-09	Metabolism	Carbohydrate Metabolism	Inositol phosphate metabolism	K03336 - 3D-(3,5/4)-trihydroxycyclohexane-1,2-dione hydrolase iolD [EC:3.7.1.-]
3.713	3.07E-07	Unclassified	Protein families: metabolism	Peptidases and inhibitors	K08600 - sortase B srtB
3.712	3.04E-08	Environmental Information Processing	Signal Transduction	Two-component system	K03739 - membrane protein involved in D-alanine export DltB
3.693	4.10E-10	Unclassified	Protein families: genetic information processing	Chaperons and folding catalysts	K04078 - chaperonin GroES

3.690	3.53E-09	Genetic Information Processing	Translation	ribosome	K02933 - large subunit ribosomal protein L6, RP-L6, MRPL6, rplF
3.680	8.12E-09	Cellular Processes	Cell Motility	Flagellar assembly	K02411 - flagellar assembly protein FliH
3.658	3.79E-08	Genetic Information Processing	Folding, Sorting and Degradation	Protein export	K03070 - preprotein translocase subunit SecA
3.607	5.80E-06	Metabolism	Carbohydrate Metabolism	glycolysis / Gluconeogenesis	K00161 - pyruvate dehydrogenase E1 component subunit alpha pdhA [EC:1.2.4.1]
3.599	2.15E-10	Environmental Information Processing	Membrane transport	ABC transporters	K15583 - oligopeptide transport system ATP-binding protein oppD
3.588	2.95E-08	Unclassified	Protein families: genetic information processing	Transfer RNA biogenesis	K07560 - D-aminoacyl-tRNA deacylase dtd
3.549	1.06E-07	Metabolism	Lipid Metabolism	Fatty acid biosynthesis	K00208 - enoyl-[acyl-carrier protein] reductase I FabI [EC:1.3.1.9]
3.507	6.23E-11	Cellular Processes	Cell Motility	Flagellar assembly	K02390 - flagellar hook protein FlgE
3.492	6.23E-07	Unclassified	Protein families: genetic information processing	Translation factors	K02357 - elongation factor Ts, tsf, TSFM
3.483	6.76E-11	Metabolism	Carbohydrate Metabolism	Glycolysis / Gluconeogenesis	K01835 - phosphoglucomutase pgm [EC:5.4.2.2]
3.469	4.05E-05	Metabolism	Metabolism of Cofactors and Vitamins	Thiamine metabolism	K00878 - hydroxyethylthiazole kinase thiM [EC:2.7.1.50]
3.435	5.87E-11	Metabolism	Metabolism of Cofactors and Vitamins	Thiamine metabolism	K06949 - ribosome biogenesis GTPase/thiamine phosphate phosphatase rsgA, rsgC
3.424	2.81E-08	Unclassified	-	-	K08972 - putative membrane protein (poorly characterized)
3.420	9.33E-07	Metabolism	Amino Acid Metabolism	Lysine biosynthesis	K01714 - dihydrodipicolinate synthase dapA [EC:4.2.1.52]
3.414	6.33E-08	Environmental Information Processing	Membrane transport	ABC transporters	K15770 - putative arabinogalactan oligomer transport system substrate-binding protein ganO, cycB
3.409	1.90E-07	Unclassified	Unclassified: metabolism	Others	K06999 - phospholipase/carboxylesterase
3.397	9.22E-09	Metabolism	Metabolism of Cofactors and Vitamins	Porphyrin and chlorophyll metabolism	K01845 - glutamate-1-semialdehyde 2,1-aminomutase hemL [EC:5.4.3.8]
3.334	4.38E-09	Metabolism	Lipid Metabolism	Glycerolipid metabolism	K00901 - diacylglycerol kinase DgkA
3.331	1.05E-05	Environmental Information Processing	Signal Transduction	Two-component system	K14205 - phosphatidylglycerol lysyltransferase fmtC, mprF [EC:2.3.2.3]
3.326	1.35E-08	Unclassified	Protein families: metabolism	Peptidases and inhibitors	K01417 - extracellular elastinolytic metalloproteinase MEP
3.319	7.39E-06	Genetic Information Processing	Translation	RNA transport	K00784 - ribonuclease Z rnz [EC:3.1.26.11]
3.304	5.40E-06	Environmental Information Processing	Membrane transport	ABC transporters	K17319 - putative aldouronate transport system permease protein lplB
3.301	3.01E-06	Metabolism	Metabolism of Other Amino Acids	D-Alanine metabolism	K03367 - D-alanine--poly(phosphoribitol) ligase subunit 1 DltA

3.292	6.80E-07	Metabolism	Carbohydrate Metabolism	glycolysis / Gluconeogenesis	K01803 - triosephosphate isomerase (TIM) tpiA, TPI [EC:5.3.1.1]
3.285	1.41E-08	Unclassified	Protein families: signaling and cellular processes	Transporters	K02029 - polar amino acid transport system permease protein ABC.PA.P
3.262	3.71E-06	Metabolism	Metabolism of Other Amino Acids	D-Alanine metabolism	K14188 - D-alanine--poly(phosphoribitol) DltC
2.997	3.16E-04	Metabolism	Amino Acid Metabolism	Phenylalanine, tyrosine and tryptophan biosynthesis	K01657 - anthranilate synthase component I trpE [EC:4.1.3.27]
2.961	7.59E-05	Cellular Processes	Cellular community - prokaryotes	Quorum sensing	K02035 - peptide/nickel transport system substrate-binding protein, ABC.PE.S
2.960	5.87E-11	Unclassified	Protein families: metabolism	Protein kinases	K08884 - serine/threonine protein kinase, bacterial
2.884	2.39E-04	Organismal Systems	Digestive system	Mineral absorption.	K07213 - copper chaperone ATOX1, ATX1, copZ, golB
2.850	5.38E-05	Unclassified	-	-	K06973 - uncharacterized protein
2.849	1.67E-04	Metabolism	Amino Acid Metabolism	Histidine metabolism	K00817 - histidinol-phosphate aminotransferase hisC [EC:2.6.1.9]
2.818	5.71E-07	Metabolism	Metabolism of Cofactors and Vitamins	Folate biosynthesis	K03342 - para-aminobenzoate synthetase / 4-amino-4-deoxychorismate lyase pabBC [EC:2.6.1.85 4.1.3.38]
2.814	1.90E-07	Genetic Information Processing	Translation	Aminoacyl-tRNA biosynthesis	K01869 - leucyl-tRNA synthetase leuS [EC:6.1.1.4]
2.799	1.10E-04	Unclassified	Protein families: genetic information processing	Transcription machinery	K17763 - rsbT co-antagonist protein RsbR
2.781	1.99E-08	Metabolism	Lipid Metabolism	Glycerolipid metabolism	K08591 - acyl phosphate:glycerol-3-phosphate acyltransferase PlsY
2.729	1.63E-07	Unclassified	-	-	K07170 - L-methionine (R)-S-oxide reductase
2.709	6.25E-08	Environmental Information Processing	Membrane transport	ABC transporters	K16013 - ATP-binding cassette, subfamily C, bacterial CydD
2.695	7.20E-03	Genetic Information Processing	Folding, Sorting and Degradation	RNA degradation	K04043 - molecular chaperone DnaK
2.687	1.01E-08	Metabolism	Amino Acid Metabolism	Arginine and proline metabolism	K00620 - glutamate N-acetyltransferase / amino-acid N-acetyltransferase argJ [EC:2.3.1.35 2.3.1.1]
2.667	8.57E-07	Metabolism	Carbohydrate Metabolism	Glyoxylate and dicarboxylate metabolism	K01915 - glutamine synthetase glnA [EC:6.3.1.2]
2.590	4.59E-16	Metabolism	Lipid Metabolism	Glycerolipid metabolism	K03621 - phosphate acyltransferase, PlsX
2.580	4.27E-05	Metabolism	Amino Acid Metabolism	Alanine, aspartate and glutamate metabolism	K00259 - alanine dehydrogenase ald [EC:1.4.1.1]
2.569	4.22E-06	Unclassified	Protein families: signaling and cellular processes	Transporters	K07238 - zinc transporter, ZIP family, TC.ZIP, zupT, ZRT3, ZIP2
2.543	1.93E-05	Metabolism	Energy Metabolism	Nitrogen metabolism	K00262 - glutamate dehydrogenase (NADP+) gdhA[EC:1.4.1.4]

2.541	1.27E-04	Genetic Information Processing	Translation	Aminoacyl-tRNA biosynthesis	K09698 - nondiscriminating glutamyl-tRNA synthetase gltX [EC:6.1.1.24]
2.538	5.89E-07	Unclassified	Protein families: genetic information processing	DNA replication proteins	K02469 - DNA gyrase subunit A gyrA
2.526	9.22E-09	Environmental Information Processing	Membrane transport	ABC transporters	K02016 - iron complex transport system substrate-binding protein ABC.FEV.S
2.483	2.74E-09	Metabolism	Glycan Biosynthesis and Metabolism	Peptidoglycan biosynthesis	K08724 - S-adenosylmethionine:tRNA ribosyltransferase-isomerase PbpB
2.454	7.98E-07	Metabolism	Nucleotide Metabolism	purine metabolism	K03060 - DNA-directed RNA polymerase subunit omega rpoZ [EC:2.7.7.6]
2.448	3.17E-03	Unclassified	Protein families: genetic information processing	Transcription factors	K17472 - Rrf2 family transcriptional regulator, cysteine metabolism repressor cymR
2.422	1.52E-06	Unclassified	Protein families: signaling and cellular processes	Transporters	K01992 - ABC-2 type transport system permease protein ABC-2.P
2.345	8.06E-06	Metabolism	Carbohydrate Metabolism	Inositol phosphate metabolism	K03337 - 5-deoxy-glucuronate isomerase iolB [EC:5.3.1.-]
2.342	3.29E-04	Metabolism	Metabolism of Cofactors and Vitamins	Porphyrin and chlorophyll metabolism	K06042 - precorrin-8X methylmutase cobH-cbiC [EC:5.4.1.2]
2.328	4.49E-07	Metabolism	Carbohydrate Metabolism	glycolysis / Gluconeogenesis	K00627 - pyruvate dehydrogenase E2 component (dihydrolipoamide acetyltransferase) aceF, pdhC [EC:2.3.1.12]
2.323	7.36E-03	Unclassified	Protein families: genetic information processing	Transcription factors	K10778 - AraC family transcriptional regulator, regulatory protein of adaptative response / methylated-DNA-[protein]-cysteine methyltransferase ada
2.241	1.63E-03	Unclassified	Protein families: signaling and cellular processes	Transporters	K09013 - Fe-S cluster assembly ATP-binding protein sufC
2.227	3.30E-06	Cellular Processes	Cell Motility	Flagellar assembly	K02412 - flagellum-specific ATP synthase fliI [EC:3.6.3.14]
2.221	7.64E-05	Unclassified	-	-	K07584 - uncharacterized protein, ysxB
2.215	8.12E-03	Unclassified	Protein families: genetic information processing	Ribosome biogenesis	K06180 - 23S rRNA pseudouridine1911/1915/1917 synthase rluD [EC:5.4.99.23]
2.212	8.39E-03	Unclassified	Protein families: genetic information processing	Transcription factors	K03708 - transcriptional regulator of stress and heat shock response ctsR
2.196	1.87E-09	Unclassified	-	-	K03744 - LemA protein
2.149	1.71E-04	Unclassified	Protein families: genetic	DNA repair and recombination proteins	K04485 - DNA repair protein RadA/Sms

			information processing		
2.134	5.88E-06	Metabolism	Glycan Biosynthesis and Metabolism	Peptidoglycan biosynthesis	K02563 - UDP-N-acetylglucosamine--N-acetylmuramyl-(pentapeptide) pyrophosphoryl-undecaprenol N-acetylglucosamine transferase MurG [EC:2.4.1.227]
2.128	2.86E-04	Unclassified	Protein families: signaling and cellular processes	Two-component system	K07183 - two-component system, response regulator / RNA-binding antiterminator nasT
2.113	7.39E-06	Unclassified	Protein families: genetic information processing	DNA replication proteins	K03169 - DNA topoisomerase III topB
2.096	8.24E-07	Genetic Information Processing	Replication and Repair	Nucleotide excision repair	K03701 - excinuclease ABC subunit A uvrA
2.064	5.22E-04	Metabolism	Carbohydrate Metabolism	Fructose and mannose metabolism	K01813 - L-rhamnose isomerase rhaA [EC:5.3.1.14]
2.058	7.26E-05	Metabolism	Carbohydrate Metabolism	Galactose metabolism	K01182 - oligo-1,6-glucosidase IMA, malL [EC:3.2.1.10]
2.041	8.13E-03	Metabolism	Metabolism of Cofactors and Vitamins	Folate biosynthesis	K03637 - molybdenum cofactor biosynthesis protein C, moaC, CNX3
2.036	7.34E-05	Metabolism	Carbohydrate Metabolism	Starch and sucrose metabolism	K05349 - beta-glucosidase bglX [EC:3.2.1.21]
2.009	7.65E-05	Metabolism	Carbohydrate Metabolism	Fructose and mannose metabolism	K01840 - phosphomannomutase manB [EC:5.4.2.8]
2.008	1.95E-04	Metabolism	Carbohydrate Metabolism	Fructose and mannose metabolism	K02795 - PTS system, mannose-specific IIC component, PTS-Man-EIIC, manY
2.007	3.17E-04	Unclassified	Protein families: genetic information processing	Translation factors	K01056 - peptidyl-tRNA hydrolase, PTH1 family, pth, spoVC
1.984	4.26E-04	Genetic Information Processing	Replication and Repair	Homologous recombination	K03551 - holliday junction DNA helicase RuvB [EC:3.6.4.12]
1.944	1.92E-04	Unclassified	Protein families: genetic information processing	DNA repair and recombination proteins	K03547 - DNA repair protein SbcD/Mre11
1.916	1.20E-04	Environmental Information Processing	Signal Transduction	Two-component system	K02406 - flagellin fliC
1.901	6.40E-05	Unclassified	-	-	K06962 - uncharacterized protein
1.863	9.78E-06	Metabolism	Amino Acid Metabolism	Valine, leucine and isoleucine degradation	K09699 - 2-oxoisovalerate dehydrogenase E2 component (dihydrolipoyl transacylase) DBT, bkdB [EC:2.3.1.168]
1.860	2.18E-06	Genetic Information Processing	Replication and Repair	Base excision repair	K03648 - uracil-DNA glycosylase UNG [EC:3.2.2.27]
1.847	3.30E-04	Cellular Processes	Cell Motility	Flagellar assembly	K02409 - flagellar M-ring protein FliF
1.767	2.05E-04	Unclassified	Protein families: signaling and cellular processes	Transporters	K11203 - PTS system, fructose-specific IIC-like component, PTS-Fru2-EIIC
1.762	3.17E-04	Unclassified	Unclassified: signaling and	Cell growth	K06412 - stage V sporulation protein G, spoVG

			cellular processes		
1.704	6.09E-04	Unclassified	-	-	K08981 - putative membrane protein
1.690	4.05E-04	Unclassified	Protein families: metabolism	Protein kinases	K00936 - two-component system, sensor histidine kinase PdtaS
1.667	7.61E-04	Unclassified	Unclassified: metabolism	Enzymes with EC numbers	K08317 - uncharacterized oxidoreductase ybdH
1.648	9.59E-05	Unclassified	Protein families: signaling and cellular processes	Transporters	K08161 - MFS transporter, DHA1 family, multidrug resistance protein mdtG
1.637	1.71E-03	Metabolism	Amino Acid Metabolism	Phenylalanine, tyrosine and tryptophan biosynthesis	K00014 - shikimate dehydrogenase aroE [EC:1.1.1.25]
1.618	9.78E-04	Metabolism	Lipid Metabolism	Fatty acid biosynthesis	K09458 - 3-oxoacyl-[acyl-carrier-protein] synthase II fabF, OXSM, CEM1 [EC:2.3.1.179]
1.606	1.09E-05	Unclassified	-	-	K09762 - uncharacterized protein
1.604	4.76E-03	Environmental Information Processing	Signal Transduction	Two-component system	K11618 - two-component system, NarL family, response regulator LiaR
1.590	2.14E-04	Environmental Information Processing	Membrane transport	ABC transporters	K09815 - zinc transport system substrate-binding protein znuA
1.559	5.49E-04	Metabolism	Nucleotide Metabolism	purine metabolism	K00962 - polyribonucleotide nucleotidyltransferase pnp, PNPT1 [EC:2.7.7.8]
1.558	2.47E-02	Metabolism	Nucleotide Metabolism	Pyrimidine metabolism	K00384 - thioredoxin reductase (NADPH) trxB [EC:1.8.1.9]
1.549	1.00E-06	Metabolism	Metabolism of Cofactors and Vitamins	Porphyrin and chlorophyll metabolism	K04720 - threonine-phosphate decarboxylase cobD [EC:4.1.1.81]
1.523	9.09E-03	Unclassified	Protein families: signaling and cellular processes	Transporters	K03442 - small conductance mechanosensitive channel mscS
1.508	3.74E-04	Unclassified	Unclassified: metabolism	Enzymes with EC numbers	K04844 - hypothetical glycosyl hydrolase ycjT
1.502	8.54E-03	Environmental Information Processing	Signal Transduction	Two-component system	K07720 - two-component system, response regulator YesN
1.478	3.15E-04	Metabolism	Metabolism of Other Amino Acids	D-Alanine metabolism	K01921 - D-alanine-D-alanine ligase ddl [EC:6.3.2.4]
1.460	9.87E-05	Metabolism	Carbohydrate Metabolism	Fructose and mannose metabolism	K02770 - PTS system, fructose-specific IIC component, PTS-Fru-EIIC, fruA
1.459	6.80E-03	Unclassified	Protein families: metabolism	Peptidases and inhibitors	K01419 - ATP-dependent HslUV protease, peptidase subunit HslV, ClpQ
1.455	9.44E-04	Metabolism	Metabolism of Cofactors and Vitamins	Nicotinate and nicotinamide metabolism	K03742 - nicotinamide-nucleotide amidase pncC
1.393	4.11E-04	Unclassified	Unclassified: metabolism	Enzymes with EC numbers	K21285 - teichoic acid glycerol-phosphate primase TagB
1.350	3.19E-02	Metabolism	Nucleotide Metabolism	Pyrimidine metabolism	K17828 - dihydroorotate dehydrogenase (NAD+) catalytic subunit pyrDI
1.345	2.62E-03	Metabolism	Metabolism of Cofactors and Vitamins	Porphyrin and chlorophyll metabolism	K02231 - adenosylcobinamide kinase / adenosylcobinamide-phosphate guanylyltransferase cobP, cobU [EC:2.7.1.156 2.7.7.62]
1.325	3.55E-04	Unclassified	Protein families:	Transporters	K08170 - MFS transporter, DHA2 family, multidrug resistance protein, norB, norC

			signaling and cellular processes		
1.322	4.97E-03	Metabolism	Nucleotide Metabolism	purine metabolism	K01952 - phosphoribosylformylglycinamidine synthase purL [EC:6.3.5.3]
1.262	9.87E-03	Metabolism	Metabolism of Cofactors and Vitamins	Porphyrin and chlorophyll metabolism	K02188 - cobalt-precorrin-5B (C1)-methyltransferase cbiD [EC:2.1.1.195]
1.249	8.84E-06	Metabolism	Carbohydrate Metabolism	Fructose and mannose metabolism	K01628 - L-fuculose-phosphate aldolase fucA [EC:4.1.2.17]
1.237	1.49E-05	Unclassified	Protein families: genetic information processing	Ribosome biogenesis	K07566 - L-threonylcarbamoyladenylate synthase, tsaC, rimN, SUA5
1.201	1.23E-02	Metabolism	Energy Metabolism	Oxidative phosphorylation	K02115 - F-type H+-transporting ATPase subunit gamma ATPF1G, atpG [EC:3.6.3.14]
1.170	1.40E-02	Metabolism	Carbohydrate Metabolism	C5-Branched dibasic acid metabolism	K01703 - 3-isopropylmalate/(R)-2-methylmalate dehydratase large subunit leuC [EC:4.2.1.33 4.2.1.35]
1.169	1.58E-03	Unclassified	-	-	K06198 - competence protein CoiA
1.168	1.51E-03	Metabolism	Carbohydrate Metabolism	glycolysis / Gluconeogenesis	K01624 - fructose-bisphosphate aldolase, class II, fbaA [EC:4.1.2.13]
1.165	1.03E-02	Metabolism	Metabolism of Cofactors and Vitamins	Nicotinate and nicotinamide metabolism	K01916 - NAD+ synthase nadE [EC:6.3.1.5]
1.163	4.45E-03	Unclassified	Protein families: genetic information processing	Chaperons and folding catalysts	K07533 - foldase protein PrsA
1.156	2.21E-02	Metabolism	Carbohydrate Metabolism	pyruvate metabolism	K00656 - formate C-acetyltransferase pflD [EC:2.3.1.54]
1.086	6.90E-03	Unclassified	-	-	K09764 - uncharacterized protein
1.083	4.76E-03	Genetic Information Processing	Translation	ribosome	K02897 - large subunit ribosomal protein L25
1.061	2.24E-03	Unclassified	Protein families: genetic information processing	DNA repair and recombination proteins	K01669 - deoxyribodipyrimidine photo-lyase phrB
1.052	1.05E-02	Environmental Information Processing	Membrane transport	ABC transporters	K02007 - cobalt/nickel transport system permease protein cbiM
1.052	5.72E-03	Environmental Information Processing	Membrane transport	ABC transporters	K18104 - ATP-binding cassette, subfamily B, bacterial AbcA/BmrA
1.047	3.09E-03	Genetic Information Processing	Translation	RNA transport	K00974 - tRNA nucleotidyltransferase (CCA-adding enzyme) [EC:2.7.7.72 3.1.3.- 3.1.4.-]
1.032	8.16E-03	Unclassified	-	-	K09935 - uncharacterized protein
1.017	2.87E-02	Unclassified	Protein families: genetic information processing	Translation factors	K02493 - release factor glutamine methyltransferase, hemK, prmC
1.011	2.33E-02	Metabolism	Nucleotide Metabolism	purine metabolism	K01951 - GMP synthase (glutamine-hydrolysing) guaA [EC:6.3.5.2]
0.987	2.13E-03	Environmental Information Processing	Signal Transduction	Two-component system	K03740-D-alanine transfer protein dltD
0.983	5.12E-04	Unclassified	Protein families:	Transporters	K02057 - simple sugar transport system permease protein ABC.SS.P

			signaling and cellular processes		
0.929	4.22E-05	Metabolism	Metabolism of Cofactors and Vitamins	Pantothenate and CoA biosynthesis	K00997 - holo-[acyl-carrier protein] synthase acpS [EC:2.7.8.7]
0.924	5.00E-05	Unclassified	Protein families: signaling and cellular processes	Transporters	K02025 - multiple sugar transport system permease protein ABC.MS.P
0.905	7.88E-03	Metabolism	Carbohydrate Metabolism	glycolysis / Gluconeogenesis	K00134 - glyceraldehyde 3-phosphate dehydrogenase GAPDH, gapA [EC:1.2.1.12]
0.878	3.77E-03	Metabolism	Energy Metabolism	Nitrogen metabolism	K00265 - glutamate synthase (NADPH/NADH) large chain gltB [EC:1.4.1.13 1.4.1.14]
0.858	9.87E-03	Genetic Information Processing	Replication and Repair	Base excision repair	K03652 - DNA-3-methyladenine glycosylase MPG [EC:3.2.2.21]
0.843	5.95E-03	Metabolism	Amino Acid Metabolism	Cysteine and methionine metabolism	K00547 - homocysteine S-methyltransferase mmuM [EC:2.1.1.10]
0.834	1.34E-02	Metabolism	Carbohydrate Metabolism	Fructose and mannose metabolism	K00882 - 1-phosphofructokinase fruK [EC:2.7.1.56]
0.832	1.29E-02	Unclassified	Protein families: signaling and cellular processes	Transporters	K11741 - quaternary ammonium compound-resistance protein SugE
0.817	8.68E-05	Unclassified	Protein families: genetic information processing	Membrane trafficking	K06902 - MFS transporter, UMF1 family
0.805	2.41E-02	Unclassified	Protein families: genetic information processing	Chromosome and associated proteins	K04047 - starvation-inducible DNA-binding protein dps
0.799	1.27E-02	Unclassified	Unclassified: metabolism	Enzymes with EC numbers	K07313 - serine/threonine protein phosphatase 1, pphA
0.779	7.18E-03	Environmental Information Processing	Membrane transport	ABC transporters	K02008 - cobalt/nickel transport system permease protein cbiQ
0.760	4.20E-02	Metabolism	Carbohydrate Metabolism	glycolysis / Gluconeogenesis	K00382 - dihydrolipoamide dehydrogenase, DLD, lpd, pdhD
0.755	2.69E-02	Unclassified	Protein families: genetic information processing	Transcription machinery	K11762 - rsbT antagonist protein RsbS
0.739	1.58E-02	Unclassified	Unclassified: signaling and cellular processes	Transporters	K03324 - phosphate:Na+ symporter, yjbB
0.720	4.15E-02	Cellular Processes	Cell Motility	Bacterial chemotaxis	K02416 - flagellar motor switch protein FliM
0.710	4.87E-04	Cellular Processes	Cell Growth and Death	Cell cycle - Caulobacter	K03544 - ATP-dependent Clp protease ATP-binding subunit ClpX
0.678	3.88E-02	Metabolism	Nucleotide Metabolism	purine metabolism	K01588 - 5-(carboxyamino)imidazole ribonucleotide mutase purE [EC:5.4.99.18]
0.651	2.80E-02	Unclassified	Unclassified: metabolism	Enzymes with EC numbers	K03820 apolipoprotein N-acyltransferase Lnt

0.583	1.54E-02	Environmental Information Processing	Signal Transduction	Two-component system	K00575 - chemotaxis protein methyltransferase CheR [EC:2.1.1.80]
0.570	1.63E-02	Metabolism	Metabolism of Other Amino Acids	Phosphonate and phosphinate metabolism	K06193 - phosphonoacetate hydrolase phnA [EC:3.11.1.2]
0.561	6.28E-03	Unclassified	Protein families: genetic information processing	Transfer RNA biogenesis	K00773 - queuine tRNA-ribosyltransferase tgt, QTRT1 [EC:2.4.2.29]
0.558	1.23E-02	Genetic Information Processing	Folding, Sorting and Degradation	Protein export	K03101 - signal peptidase II lspA [EC:3.4.23.36]
0.505	3.99E-02	Metabolism	Amino Acid Metabolism	Phenylalanine, tyrosine and tryptophan biosynthesis	K01736 - chorismate synthase aroC [EC:4.2.3.5]
0.416	1.14E-02	Metabolism	Carbohydrate Metabolism	Fructose and mannose metabolism	K02798 - PTS system, mannitol-specific IIA component, PTS-Mtl-EIIA, mtlA, cmtB [EC:2.7.1.69]
0.274	3.56E-02	Metabolism	Metabolism of Terpenoids and Polyketides	Terpenoid backbone biosynthesis	K00991 - 2-C-methyl-D-erythritol 4-phosphate cytidylyltransferase ispD [EC:2.7.7.60]
-0.375	1.89E-02	Metabolism	Metabolism of Other Amino Acids	beta-Alanine metabolism	K01918 - pantoate--beta-alanine ligase panC [EC:6.3.2.1]
-0.390	2.40E-02	Metabolism	Carbohydrate Metabolism	Fructose and mannose metabolism	K02796 - PTS system, mannose-specific IID component, PTS-Man-EIID, manZ
-0.418	7.36E-03	Metabolism	Energy Metabolism	Oxidative phosphorylation	K02827 - cytochrome aa3-600 menaquinol oxidase subunit I qoxB [EC:1.10.3.12]
-0.424	4.46E-02	Genetic Information Processing	Replication and Repair	Mismatch repair	K03572 - DNA mismatch repair protein MutL
-0.434	4.69E-02	Metabolism	Metabolism of Cofactors and Vitamins	Porphyrin and chlorophyll metabolism	K01772 - ferrochelatase hemH [EC:4.99.1.1]
-0.460	2.64E-02	Metabolism	Amino Acid Metabolism	Lysine biosynthesis	K05822 - tetrahydrodipicolinate N-acetyltransferase dapH, dapD [EC:2.3.1.89]
-0.463	4.67E-02	Metabolism	Energy Metabolism	Oxidative phosphorylation	K02829 - cytochrome aa3-600 menaquinol oxidase subunit IV qoxD [EC:1.10.3.12]
-0.464	4.72E-02	Metabolism	Metabolism of Cofactors and Vitamins	Porphyrin and chlorophyll metabolism	K16651 - L-threonine kinase pduX
-0.468	1.83E-02	Unclassified	Protein families: genetic information processing	Transcription factors	K03719 - Lrp/AsnC family transcriptional regulator, leucine-responsive regulatory protein
-0.488	1.19E-02	Unclassified	Protein families: genetic information processing	Transfer RNA biogenesis	K03216 - tRNA (cytidine/uridine-2'-O-)-methyltransferase, cspR, trmL
-0.498	1.11E-02	Genetic Information Processing	Translation	Aminoacyl-tRNA biosynthesis	K01893 - asparaginyl-tRNA synthetase NARS, asnS [EC:6.1.1.22]
-0.498	5.69E-03	Unclassified	Unclassified: metabolism	Glycan metabolism	K07027 - glycosyltransferase 2 family protein
-0.502	1.05E-02	Metabolism	Carbohydrate Metabolism	pyruvate metabolism	K01734 - methylglyoxal synthase mgsA [EC:4.2.3.3]
-0.504	4.76E-02	Metabolism	Carbohydrate Metabolism	Fructose and mannose metabolism	K02769 - PTS system, fructose-specific IIB component [EC:2.7.1.69], PTS-Fru-EIIB, fruA

-0.504	2.49E-02	Cellular Processes	Cell Motility	Flagellar assembly	K02400 - flagellar biosynthesis protein FlhA
-0.510	1.24E-02	Genetic Information Processing	Translation	ribosome	K02935 - large subunit ribosomal protein L7/L12
-0.512	4.32E-02	Metabolism	Metabolism of Cofactors and Vitamins	Nicotinate and nicotinamide metabolism	K03517 - quinolinate synthase nadA [EC:2.5.1.72]
-0.519	1.61E-03	Unclassified	-	-	K06889 - uncharacterized protein
-0.523	9.76E-03	Unclassified	Protein families: genetic information processing	Transfer RNA biogenesis	K03439 - tRNA (guanine-N7-)-methyltransferase, trmB, METTL1, TRM8
-0.547	4.68E-03	Genetic Information Processing	Translation	ribosome	K02874 - large subunit ribosomal protein L14
-0.549	3.27E-02	Genetic Information Processing	Translation	ribosome	K02909 - large subunit ribosomal protein L31
-0.554	1.18E-03	Environmental Information Processing	Membrane transport	ABC transporters	K16785 - energy-coupling factor transport system permease protein ecfT
-0.580	7.10E-03	Unclassified	Protein families: metabolism	Protein kinases	K04757 - serine/threonine-protein kinase RsbW
-0.580	4.35E-03	Metabolism	Carbohydrate Metabolism	Ascorbate and aldarate metabolism	K03475 - PTS system, ascorbate-specific IIC component, PTS-Ula-EIIC, ulaA, sgaT
-0.588	4.33E-02	Metabolism	Carbohydrate Metabolism	Amino sugar and nucleotide sugar metabolism	K02564 - glucosamine-6-phosphate deaminase nagB [EC:3.5.99.6]
-0.602	3.32E-03	Unclassified	Unclassified: signaling and cellular processes	Transport	K03321 - sulfate permease, SulP family
-0.606	1.14E-02	Unclassified	Protein families: genetic information processing	Ribosome biogenesis	K07056 - 16S rRNA (cytidine1402-2'-O)-methyltransferase, rsmI
-0.606	7.04E-03	Cellular Processes	Cell Motility	Bacterial chemotaxis	K02417 - flagellar motor switch protein FliN/FliY
-0.611	3.88E-02	Unclassified	Protein families: genetic information processing	Transfer RNA biogenesis	K06173 - tRNA pseudouridine38-40 synthase
-0.614	9.10E-03	Unclassified	-	-	K07058 - membrane protein
-0.628	3.59E-04	Unclassified	-	-	K03095 - SprT-like protein, sprL
-0.643	3.03E-04	Metabolism	Energy Metabolism	Sulfur metabolism	K00641 - homoserine O-acetyltransferase metX [EC:2.3.1.31]
-0.645	2.33E-02	Metabolism	Lipid Metabolism	Glycerophospholipid metabolism	K06131 - cardiolipin synthase clsA_B [EC:2.7.8.-]
-0.646	9.41E-03	Genetic Information Processing	Translation	Aminoacyl-tRNA biosynthesis	K01883 - cysteinyl-tRNA synthetase cysS [EC:6.1.1.16]
-0.655	3.37E-03	Unclassified	Protein families: genetic information processing	Ribosome biogenesis	K03789 - [ribosomal protein S18]-alanine N-acetyltransferase rimI

-0.667	9.26E-03	Genetic Information Processing	Replication and Repair	Homologous recombination	K03581 - exodeoxyribonuclease V alpha subunit recD [EC:3.1.11.5]
-0.668	1.08E-02	Metabolism	Metabolism of Cofactors and Vitamins	Nicotinate and nicotinamide metabolism	K00858 - NAD+ kinase ppnk, NADK [EC:2.7.1.23]
-0.674	1.96E-02	Unclassified	-	-	K03545 - trigger factor tig
-0.681	6.90E-04	Metabolism	Carbohydrate Metabolism	Amino sugar and nucleotide sugar metabolism	K00790 - UDP-N-acetylglucosamine 1-carboxyvinyltransferase MurA
-0.684	9.65E-03	Metabolism	Carbohydrate Metabolism	Starch and sucrose metabolism	K07024 - sucrose-6-phosphatase, SPP
-0.686	4.07E-03	Unclassified	Protein families: signaling and cellular processes	Transporters	K05567 - multicomponent Na+:H+ antiporter subunit C, mnhC, mrpC
-0.688	8.16E-03	Unclassified	-	-	K07052 - uncharacterized protein
-0.691	2.31E-03	Metabolism	Metabolism of Cofactors and Vitamins	Porphyrin and chlorophyll metabolism	K05895 - precorrin-6X reductase cobK-cbiJ [EC:1.3.1.54]
-0.692	5.70E-04	Unclassified	Protein families: signaling and cellular processes	Transporters	K08227 - MFS transporter, LPLT family, lysophospholipid transporter
-0.693	1.91E-03	Metabolism	Carbohydrate Metabolism	glycolysis / Gluconeogenesis	K15634 - probable phosphoglycerate mutase gpmB [EC:5.4.2.1]
-0.701	4.76E-02	Environmental Information Processing	Membrane transport	ABC transporters	K11704 - iron/zinc/copper transport system substrate-binding protein mtsA
-0.710	1.06E-06	Genetic Information Processing	Folding, Sorting and Degradation	Protein processing in endoplasmic reticulum	K04079 - molecular chaperone HtpG
-0.711	2.69E-02	Metabolism	Lipid Metabolism	Fatty acid biosynthesis	K00645 - [acyl-carrier-protein] S-malonyltransferase FabD
-0.717	2.14E-02	Unclassified	-	-	K07048 - phosphotriesterase-related protein PTER, php
-0.736	2.85E-03	Unclassified	Protein families: genetic information processing	Transcription machinery	K05518 - phosphoserine phosphatase RsbX
-0.736	2.21E-05	Unclassified	Protein families: genetic information processing	Ribosome biogenesis	K06183 - 16S rRNA pseudouridine516 synthase, rsuA
-0.740	1.82E-03	Metabolism	Metabolism of Cofactors and Vitamins	Porphyrin and chlorophyll metabolism	K04032 - ethanolamine utilization cobalamin adenosyltransferase eutT [EC:2.5.1.17]
-0.742	1.18E-04	Metabolism	Nucleotide Metabolism	Pyrimidine metabolism	K00761 - uracil phosphoribosyltransferase upp, UPRT [EC:2.4.2.9]
-0.745	2.83E-02	Unclassified	Protein families: genetic information processing	Transcription factors	K07738 - transcriptional repressor NrdR
-0.745	2.55E-06	Metabolism	Metabolism of Cofactors and Vitamins	Porphyrin and chlorophyll metabolism	K02190 - sirohydrochlorin cobaltochelatase cbiK[EC:4.99.1.3]
-0.753	1.53E-03	Environmental Information Processing	Membrane transport	ABC transporters	K15580 - oligopeptide transport system substrate-binding protein oppA, mppA

-0.757	4.06E-04	Unclassified	Unclassified: metabolism	Enzymes with EC numbers	K09773 - [pyruvate, water dikinase]-phosphate phosphotransferase / [pyruvate, water dikinase] kinase ppsR
-0.759	5.69E-03	Unclassified	Protein families: signaling and cellular processes	Transporters	K03449 - MFS transporter, CP family, cyanate transporter, MFS.CP
-0.762	1.71E-02	Metabolism	Metabolism of Terpenoids and Polyketides	Terpenoid backbone biosynthesis	K01770 - 2-C-methyl-D-erythritol 2,4-cyclodiphosphate synthase ispF [EC:4.6.1.12]
-0.773	1.48E-02	Metabolism	Carbohydrate Metabolism	Fructose and mannose metabolism	K02793 - PTS system, mannose-specific IIA component [EC:2.7.1.69], PTS-Man-EIIA, manX
-0.774	3.46E-04	Unclassified	Protein families: signaling and cellular processes	Prokaryotic defense system	K07171 - mRNA interferase MazF, ndoA, chpA
-0.780	3.78E-04	Environmental Information Processing	Membrane transport	Phosphotransferase system (PTS)	K11189 - phosphocarrier protein PTS-HPR
-0.789	5.97E-06	Cellular Processes	Cell Growth and Death	Cell cycle - Caulobacter	K03590 - cell division protein ftsA
-0.791	1.71E-04	Unclassified	-	-	K09167 - uncharacterized protein
-0.794	8.33E-04	Unclassified	-	-	K07742 - uncharacterized protein ylxR
-0.800	8.11E-03	Metabolism	Metabolism of Cofactors and Vitamins	Pantothenate and CoA biosynthesis	K13038 - phosphopantothenoylcysteine decarboxylase / phosphopantothenate--cysteine ligase coaBC[EC:4.1.1.36 6.3.2.5]
-0.808	3.84E-04	Metabolism	Metabolism of Terpenoids and Polyketides	Terpenoid backbone biosynthesis	K03526 - (E)-4-hydroxy-3-methylbut-2-enyl-diphosphate synthase gcpE, ispG [EC:1.17.7.1]
-0.819	2.99E-02	Genetic Information Processing	Translation	Aminoacyl-tRNA biosynthesis	K01870 - isoleucyl-tRNA synthetase ileS [EC:6.1.1.5]
-0.824	2.27E-03	Unclassified	Protein families: metabolism	Peptidases and inhibitors	K03797 - carboxyl-terminal processing protease prc, ctpA
-0.828	8.62E-04	Genetic Information Processing	Folding, Sorting and Degradation	RNA degradation	K12574 - ribonuclease J [EC:3.1.-.-]
-0.830	7.57E-11	Metabolism	Metabolism of Other Amino Acids	D-Glutamine and D-glutamate metabolism	K01925 - UDP-N-acetylmuramoylalanine--D-glutamate ligase, MurD
-0.831	5.98E-03	Metabolism	Carbohydrate Metabolism	Amino sugar and nucleotide sugar metabolism	K12410 - NAD-dependent deacetylase npdA [EC:3.5.1.-]
-0.837	2.21E-03	Genetic Information Processing	Translation	Aminoacyl-tRNA biosynthesis	K01879 - glycyl-tRNA synthetase beta chain glyS [EC:6.1.1.14]
-0.863	6.34E-04	Unclassified	Unclassified: metabolism	Enzymes with EC numbers	K01269 - aminopeptidase
-0.883	4.02E-03	Genetic Information Processing	Translation	ribosome	K02911 - large subunit ribosomal protein L32
-0.884	2.12E-05	Metabolism	Energy Metabolism	Methane metabolism	K00831 - phosphoserine aminotransferase serC [EC:2.6.1.52]
-0.892	1.78E-03	Metabolism	Energy Metabolism	Oxidative phosphorylation	K02259 - cytochrome c oxidase assembly protein subunit 15, COX15, ctaA
-0.897	2.69E-05	Metabolism	Nucleotide Metabolism	purine metabolism	K02338 - DNA polymerase III subunit beta dnaN [EC:2.7.7.7]
-0.907	5.96E-03	Unclassified	-	-	K09770 - uncharacterized protein

-0.912	1.30E-06	Metabolism	Metabolism of Terpenoids and Polyketides	Terpenoid backbone biosynthesis	K00054 - hydroxymethylglutaryl-CoA reductase mvaA [EC:1.1.1.88]
-0.918	5.95E-03	Unclassified	Protein families: genetic information processing	Transfer RNA biogenesis	K03495 - tRNA uridine 5-carboxymethylaminomethyl modification enzyme gidA, mnmG, MT01
-0.926	1.77E-05	Genetic Information Processing	Folding, Sorting and Degradation	Protein export	K03106 - signal recognition particle subunit SRP54, Ffh
-0.928	2.26E-03	Genetic Information Processing	Translation	ribosome	K02986 - small subunit ribosomal protein S4
-0.931	1.59E-03	Unclassified	-	-	K09157 - uncharacterized protein
-0.935	6.50E-03	Unclassified	Protein families: metabolism	Peptidoglycan biosynthesis and degradation proteins	K01448 - N-acetylmuramoyl-L-alanine amidase amiABC
-0.937	4.32E-02	Environmental Information Processing	Membrane transport	ABC transporters	K11051 - multidrug/hemolysin transport system permease protein cylB
-0.942	3.17E-04	Metabolism	Amino Acid Metabolism	Phenylalanine, tyrosine and tryptophan biosynthesis	K01658 - anthranilate synthase component II trpG [EC:4.1.3.27]
-0.946	1.17E-03	Metabolism	Metabolism of Cofactors and Vitamins	Porphyrin and chlorophyll metabolism	K03394 - precorrin-2/cobalt-factor-2 C20-methyltransferase cobI-cbiL [EC:2.1.1.130 2.1.1.151]
-0.951	2.34E-05	Unclassified	Protein families: genetic information processing	Translation factors	K02835 - peptide chain release factor 1, prfA, MTRF1, MRF1
-0.955	1.35E-04	Cellular Processes	Cell Motility	Flagellar assembly	K02419 - flagellar biosynthetic protein FliP
-0.958	1.28E-03	Metabolism	Energy Metabolism	Nitrogen metabolism	K01953 - asparagine synthase (glutamine-hydrolysing) asnB [EC:6.3.5.4]
-0.959	1.01E-06	Metabolism	Carbohydrate Metabolism	glycolysis / Gluconeogenesis	K02777 - PTS system, glucose-specific IIA component PTS-Glc-EIIA, crr [EC:2.7.1.69]
-0.960	8.29E-05	Metabolism	Amino Acid Metabolism	Arginine and proline metabolism	K00145 - N-acetyl-gamma-glutamyl-phosphate reductase argC [EC:1.2.1.38]
-0.965	3.97E-04	Unclassified	Protein families: metabolism	Peptidoglycan biosynthesis and degradation proteins	K06078 -murein lipoprotein Lpp
-0.966	1.93E-12	Metabolism	Metabolism of Other Amino Acids	D-Alanine metabolism	K01775 - alanine racemase alr [EC:5.1.1.1]
-0.968	7.22E-03	Unclassified	Protein families: genetic information processing	Ribosome biogenesis	K09761 - 16S rRNA (uracil1498-N3)-methyltransferase, rsmE
-0.970	1.09E-07	Genetic Information Processing	Replication and Repair	Mismatch repair	K03602 - exodeoxyribonuclease VII small subunit xseB [EC:3.1.11.6]
-0.975	1.35E-04	Cellular Processes	Cellular community - prokaryotes	Quorum sensing	K09936 - bacterial/archaeal transporter family-2 protein/quorum sensing
-0.976	2.25E-09	Metabolism	Amino Acid Metabolism	glycine, serine and threonine metabolism	K00872 - homoserine kinase thrB1 [EC:2.7.1.39]
-1.011	3.18E-04	Metabolism	Nucleotide Metabolism	Pyrimidine metabolism	K00943 - dTMP kinase tmk, DTYMK [EC:2.7.4.9]

-1.013	3.32E-03	Metabolism	Lipid Metabolism	Glycerolipid metabolism	K13921 - 1-propanol dehydrogenase PduQ
-1.013	1.77E-03	Metabolism	Carbohydrate Metabolism	glycolysis / Gluconeogenesis	K00162 - pyruvate dehydrogenase E1 component beta subunit pdhB
-1.017	2.35E-04	Environmental Information Processing	Membrane transport	ABC transporters	K02006 - cobalt/nickel transport system ATP-binding protein cbiO
-1.018	1.95E-04	Metabolism	Metabolism of Cofactors and Vitamins	Porphyrin and chlorophyll metabolism	K02227 - adenosylcobinamide-phosphate synthase cbiB, cobD [EC:6.3.1.10]
-1.020	2.64E-04	Metabolism	Amino Acid Metabolism	glycine, serine and threonine metabolism	K01754 - threonine dehydratase ilvA, tdcB [EC:4.3.1.19]
-1.021	4.01E-04	Metabolism	Nucleotide Metabolism	purine metabolism	K01939 - adenylosuccinate synthase purA, ADSS [EC:6.3.4.4]
-1.024	4.79E-03	Environmental Information Processing	Signal Transduction	Two-component system	K07651 - two-component system, OmpR family, sensor histidine kinase ResE [EC:2.7.13.3]
-1.029	7.02E-03	Unclassified	Unclassified: signaling and cellular processes	Transport	K03316 - monovalent cation:H+ antiporter, CPA1 family, TC,CPA1
-1.031	4.29E-02	Metabolism	Carbohydrate Metabolism	Pentose phosphate pathway	K01807 - ribose 5-phosphate isomerase A [EC:5.3.1.6], rpiA
-1.044	3.57E-05	Metabolism	Carbohydrate Metabolism	Pentose phosphate pathway	K00615 - transketolase tktA, tktB [EC:2.2.1.1]
-1.055	7.43E-05	Metabolism	Glycan Biosynthesis and Metabolism	Peptidoglycan biosynthesis	K05364 - penicillin-binding protein A, PbpA
-1.059	1.62E-04	Metabolism	Lipid Metabolism	Fatty acid biosynthesis	K02078 - acyl carrier protein AcpP
-1.060	1.02E-06	Unclassified	Protein families: genetic information processing	Transcription factors	K13653 - AraC family transcriptional regulator
-1.062	7.85E-04	Unclassified	Protein families: genetic information processing	Transfer RNA biogenesis	K00989 - ribonuclease PH [EC:2.7.7.56]
-1.067	1.05E-07	Unclassified	Protein families: signaling and cellular processes	Secretion system	K02236 - leader peptidase (prepilin peptidase) / N-methyltransferase comC
-1.076	5.80E-06	Unclassified	Unclassified: metabolism	Amino acid metabolism	K04026 - ethanolamine utilization protein EutL
-1.076	1.65E-08	Environmental Information Processing	Membrane transport	ABC transporters	K02009 - cobalt transport protein cbiN
-1.079	3.03E-04	Metabolism	Carbohydrate Metabolism	glycolysis / Gluconeogenesis	K15633 - 2,3-bisphosphoglycerate-independent phosphoglycerate mutase gpmI [EC:5.4.2.1]
-1.081	3.28E-05	Metabolism	Carbohydrate Metabolism	Starch and sucrose metabolism	K02759 - PTS system, cellobiose-specific IIA component; PTS-Cel-EIIA, celC, chbA
-1.091	3.98E-03	Environmental Information Processing	Membrane transport	ABC transporters	K02073 - D-methionine transport system substrate-binding protein metQ
-1.108	6.01E-07	Unclassified	Unclassified: metabolism	Enzymes with EC numbers	K01090 - protein phosphatase
-1.109	4.68E-04	Environmental Information Processing	Membrane transport	ABC transporters	K02015 - iron complex transport system permease protein ABC.FEV.P

-1.110	4.31E-02	Cellular Processes	Cell Growth and Death	Cell cycle - Caulobacter	K03588 - cell division protein ftsW
-1.117	1.87E-04	Metabolism	Lipid Metabolism	Glycerolipid metabolism	K00655 - 1-acyl-sn-glycerol-3-phosphate acyltransferase plsC [EC:2.3.1.51]
-1.122	2.95E-04	Genetic Information Processing	Folding, Sorting and Degradation	Protein export	K03100 - signal peptidase I lepB [EC:3.4.21.89]
-1.136	2.76E-11	Metabolism	Lipid Metabolism	Fatty acid biosynthesis	K10780 - enoyl-[acyl carrier protein] reductase III fabL [EC:1.3.1.-]
-1.137	6.65E-07	Unclassified	Unclassified: metabolism	Amino acid metabolism	K04030 - ethanolamine utilization protein EutQ
-1.145	1.51E-06	Unclassified	Unclassified: signaling and cellular processes	Transport	K03294 - basic amino acid/polyamine antiporter, APA family TC.APA
-1.148	2.30E-06	Metabolism	Nucleotide Metabolism	purine metabolism	K02341 - DNA polymerase III subunit delta holB [EC:2.7.7.7]
-1.164	1.13E-08	Metabolism	Lipid Metabolism	Glycerophospholipid metabolism	K03736 - ethanolamine ammonia-lyase small subunit eutC [EC:4.3.1.7]
-1.165	6.72E-04	Unclassified	-	-	K06994 - putative drug exporter of the RND superfamily
-1.182	6.27E-09	Environmental Information Processing	Signal Transduction	Two-component system	K11622 - lia operon protein LiaF
-1.190	1.40E-02	Unclassified	Unclassified: signaling and cellular processes	Signaling proteins	K06217 - phosphate starvation-inducible protein PhoH and related proteins
-1.195	2.17E-05	Environmental Information Processing	Membrane transport	ABC transporters	K16012 - ATP-binding cassette, subfamily C, CydC
-1.197	4.38E-06	Unclassified	Protein families: signaling and cellular processes	Transporters	K03282 - large conductance mechanosensitive channel MscL
-1.207	4.63E-04	Genetic Information Processing	Translation	Aminoacyl-tRNA biosynthesis	K01890 - phenylalanyl-tRNA synthetase beta chain FARSB, pheT [EC:6.1.1.20]
-1.208	1.81E-06	Metabolism	Amino Acid Metabolism	Histidine metabolism	K01814 - phosphoribosylformimino-5-aminoimidazole carboxamide ribotide isomerase hisA [EC:5.3.1.16]
-1.210	9.52E-06	Metabolism	Carbohydrate Metabolism	pyruvate metabolism	K00027 - malate dehydrogenase (oxaloacetate-decarboxylating) ME2, sfcA, maeA [EC:1.1.1.38]
-1.216	6.02E-08	Unclassified	Protein families: genetic information processing	Transfer RNA biogenesis	K00554 - tRNA (guanine37-N1)-methyltransferase, trmD
-1.225	7.25E-08	Metabolism	Energy Metabolism	Nitrogen metabolism	K00605 - aminomethyltransferase gcvT [EC:2.1.2.10]
-1.226	3.80E-13	Metabolism	Amino Acid Metabolism	Phenylalanine, tyrosine and tryptophan biosynthesis	K06208 - chorismate mutase aroH [EC:5.4.99.5]
-1.228	3.58E-05	Metabolism	Amino Acid Metabolism	glycine, serine and threonine metabolism	K00928 - aspartate kinase lysC [EC:2.7.2.4]
-1.230	4.45E-06	Unclassified	Unclassified: signaling and cellular processes	Transport	K03306 - inorganic phosphate transporter, PiT family TC.PIT
-1.236	3.69E-04	Metabolism	Energy Metabolism	Oxidative phosphorylation	K02826 - cytochrome aa3-600 menaquinol oxidase subunit II qoxA [EC:1.10.3.12]

-1.239	3.41E-04	Metabolism	Metabolism of Other Amino Acids	beta-Alanine metabolism	K01579 - aspartate 1-decarboxylase panD [EC:4.1.1.11]
-1.265	8.86E-06	Metabolism	Carbohydrate Metabolism	pyruvate metabolism	K01649 - 2-isopropylmalate synthase leuA [EC:2.3.3.13]
-1.266	6.28E-16	Environmental Information Processing	Membrane transport	Phosphotransferase system (PTS)	K02757 - PTS system, beta-glucosides-specific IIC component, PTS-Bgl-EIIC, bglF, bglP
-1.272	6.90E-04	Metabolism	Amino Acid Metabolism	Phenylalanine, tyrosine and tryptophan biosynthesis	K00800 - 3-phosphoshikimate 1-carboxyvinyltransferase aroA [EC:2.5.1.19]
-1.272	2.25E-05	Metabolism	Carbohydrate Metabolism	glycolysis / Gluconeogenesis	K00016 - L-lactate dehydrogenase ldh [EC:1.1.1.27]
-1.279	1.06E-10	Metabolism	Metabolism of Cofactors and Vitamins	Folate biosynthesis	K00950 - 2-amino-4-hydroxy-6-hydroxymethyldihydropteridine diphosphokinase folK [EC:2.7.6.3]
-1.279	1.45E-07	Metabolism	Nucleotide Metabolism	purine metabolism	K02340 - DNA polymerase III subunit delta holA [EC:2.7.7.7]
-1.282	2.74E-05	Unclassified	Protein families: genetic information processing	Ribosome biogenesis	K09748 - ribosome maturation factor RimP
-1.287	1.90E-07	Unclassified	Protein families: genetic information processing	Ribosome biogenesis	K00563 - 23S rRNA (guanine745-N1)-methyltransferase rlmA1
-1.296	1.68E-07	Unclassified	Protein families: signaling and cellular processes	Exosome	K02503 - histidine triad (HIT) family protein HINT1, hinT, hit
-1.303	3.09E-02	Environmental Information Processing	Membrane transport	ABC transporters	K02001 - glycine betaine/proline transport system permease protein proW
-1.306	3.76E-03	Unclassified	Protein families: genetic information processing	Chaperons and folding catalysts	K04083 - molecular chaperone Hsp33
-1.311	2.78E-10	Metabolism	Metabolism of Terpenoids and Polyketides	Terpenoid backbone biosynthesis	K00805 - heptaprenyl diphosphate synthase hepST [EC:2.5.1.30]
-1.319	3.44E-02	Unclassified	Protein families: metabolism	Peptidases and inhibitors	K01299 - carboxypeptidase Taq
-1.321	1.34E-05	Environmental Information Processing	Membrane transport	ABC transporters	K02002 - glycine betaine/proline transport system substrate-binding protein proX
-1.331	7.03E-08	Unclassified	Protein families: signaling and cellular processes	Transporters	K02027 - multiple sugar transport system substrate-binding protein ABC.MS.S
-1.335	6.26E-06	Unclassified	-	-	K09704 - uncharacterized protein
-1.336	4.34E-08	Metabolism	Energy Metabolism	Oxidative phosphorylation	K02112 - F-type H+-transporting ATPase subunit beta atpD [EC:3.6.3.14]
-1.341	1.01E-04	Unclassified	Protein families: signaling and	Transporters	K06901 - putative MFS transporter, AGZA family, xanthine/uracil permease pbuG

			cellular processes		
-1.349	1.84E-03	Cellular Processes	Cell Growth and Death	Cell cycle - Caulobacter	K11749 - regulator of sigma E protease rseP [EC:3.4.24.-]
-1.350	5.77E-08	Unclassified	Protein families: genetic information processing	Translation factors	K02838 - ribosome recycling factor frr, MRRF
-1.355	1.33E-09	Genetic Information Processing	Translation	ribosome	K02982 - small subunit ribosomal protein S3, RP-S3, rpsC
-1.358	3.46E-06	Unclassified	Protein families: genetic information processing	Transcription factors	K02538 - activator of the mannose operon, transcriptional antiterminator ManR
-1.365	1.77E-09	Metabolism	Glycan Biosynthesis and Metabolism	Peptidoglycan biosynthesis	K02545 - penicillin-binding protein 2 prime MecA
-1.367	2.14E-07	Metabolism	Lipid Metabolism	Fatty acid biosynthesis	K02371 - enoyl-[acyl carrier protein] reductase II FabK [EC:1.3.1.-]
-1.369	2.86E-03	Metabolism	Metabolism of Cofactors and Vitamins	Porphyrin and chlorophyll metabolism	K01599 - uroporphyrinogen decarboxylase hemE [EC:4.1.1.37]
-1.374	3.79E-08	Unclassified	Protein families: genetic information processing	Ribosome biogenesis	K03501 - 16S rRNA (guanine527-N7)-methyltransferase rsmG, gidB
-1.376	2.00E-02	Unclassified	-	-	K08998 - uncharacterized protein
-1.385	2.81E-05	Genetic Information Processing	Folding, Sorting and Degradation	Sulfur relay system	K11996 - adenylyltransferase and sulfurtransferase MOCS3, UBA4
-1.390	4.35E-03	Metabolism	Metabolism of Cofactors and Vitamins	Ubiquinone and other terpenoid-quinone biosynthesis	K01911 - O-succinylbenzoic acid--CoA ligase menE [EC:6.2.1.26]
-1.396	3.87E-05	Metabolism	Lipid Metabolism	Glycerolipid metabolism	K13919 - propanediol dehydratase medium subunit pduD [EC:4.2.1.28]
-1.418	4.81E-10	Metabolism	Metabolism of Cofactors and Vitamins	Porphyrin and chlorophyll metabolism	K01749 - hydroxymethylbilane synthase hemC [EC:2.5.1.61]
-1.418	8.10E-03	Unclassified	Protein families: genetic information processing	Ribosome biogenesis	K03595 - GTPase era, ERAL1
-1.426	5.23E-04	Unclassified	Protein families: signaling and cellular processes	Transporters	K05020 - glycine betaine transporter opuD, betL
-1.446	6.76E-11	Unclassified	Protein families: genetic information processing	Transcription factors	K07726 - putative transcriptional regulator
-1.449	2.92E-08	Metabolism	Nucleotide Metabolism	purine metabolism	K00526 - ribonucleoside-diphosphate reductase beta chain nrdB, nrdF [EC:1.17.4.1]
-1.452	8.06E-10	Unclassified	-	-	K09117 - uncharacterized protein

-1.473	6.47E-03	Genetic Information Processing	Replication and Repair	Base excision repair	K01246 - DNA-3-methyladenine glycosylase I tag[EC:3.2.2.20]
-1.484	3.39E-09	Metabolism	Nucleotide Metabolism	Pyrimidine metabolism	K00945 - cytidylate kinase cmk [EC:2.7.4.14]
-1.490	1.71E-07	Genetic Information Processing	Folding, Sorting and Degradation	Protein export	K12257 - SecD/SecF fusion protein
-1.505	8.46E-08	Unclassified	Protein families: genetic information processing	Ribosome biogenesis	K14540 - ribosome biogenesis GTPase A rbgA
-1.510	4.86E-10	Unclassified	Protein families: genetic information processing	Ribosome biogenesis	K00783 - 23S rRNA (pseudouridine1915-N3)-methyltransferase, rlmH
-1.511	1.96E-08	Unclassified	Protein families: genetic information processing	Messenger RNA biogenesis	K03698 - 3'-5' exoribonuclease cbf [EC:3.1.-.-]
-1.516	3.79E-08	Genetic Information Processing	Translation	ribosome	K02907 - large subunit ribosomal protein L30
-1.519	1.09E-07	Unclassified	Protein families: signaling and cellular processes	Secretion system	K02247 - competence protein ComGE
-1.522	7.43E-06	Genetic Information Processing	Replication and Repair	DNA replication	K03111 - single-strand DNA-binding protein ssb
-1.529	8.24E-07	Cellular Processes	Cell Growth and Death	Cell cycle - Caulobacter	K03589 - cell division protein ftsQ
-1.553	2.44E-04	Metabolism	Metabolism of Other Amino Acids	Selenocompound metabolism	K01874 - methionyl-tRNA synthetase MARS, metG [EC:6.1.1.10]
-1.559	1.07E-10	Metabolism	Nucleotide Metabolism	purine metabolism	K00527 - ribonucleoside-triphosphate reductase rtpR [EC:1.17.4.2]
-1.559	1.83E-03	Metabolism	Metabolism of Cofactors and Vitamins	Porphyrin and chlorophyll metabolism	K05936 - precorrin-4 C11-methyltransferase cobM, cbiF [EC:2.1.1.133]
-1.581	1.26E-06	Metabolism	Metabolism of Cofactors and Vitamins	Pantothenate and CoA biosynthesis	K03525 - type III pantothenate kinase coaX [EC:2.7.1.33]
-1.612	1.59E-04	Unclassified	Protein families: genetic information processing	Transfer RNA biogenesis	K03650 - tRNA modification GTPase mnmE, trmE, MSS1
-1.618	1.23E-04	Metabolism	Metabolism of Cofactors and Vitamins	Folate biosynthesis	K03635 - molybdopterin synthase catalytic subunit [EC:2.-.-.-]
-1.622	2.19E-08	Unclassified	Protein families: metabolism	Peptidases and inhibitors	K04773 - protease IV appA
-1.634	5.11E-06	Metabolism	Glycan Biosynthesis and Metabolism	Peptidoglycan biosynthesis	K07260 - D-alanyl-D-alanine carboxypeptidase VanY [EC:3.4.16.4]
-1.642	7.65E-05	Metabolism	Nucleotide Metabolism	purine metabolism	K01129 - dGTPase dgt [EC:3.1.5.1]

-1.644	2.18E-10	Unclassified	Unclassified: metabolism	Amino acid metabolism	K04023 - ethanolamine transporter eutH
-1.647	7.26E-05	Unclassified	Protein families: genetic information processing	DNA replication proteins	K02622 - topoisomerase IV subunit B parE
-1.657	9.01E-03	Metabolism	Amino Acid Metabolism	Phenylalanine metabolism	K00285 - D-amino-acid dehydrogenase dadA [EC:1.4.99.1]
-1.665	3.28E-08	Unclassified	Protein families: metabolism	Peptidases and inhibitors	K05995 - dipeptidase E pepE
-1.678	2.32E-25	Unclassified	Unclassified: metabolism	Amino acid metabolism	K04024 - ethanolamine utilization protein EutJ
-1.721	1.74E-10	Unclassified	Unclassified: signaling and cellular processes	Cell growth	K06346 - spoIIIJ-associated protein jag
-1.723	4.06E-18	Unclassified	-	-	K07045 - uncharacterized protein
-1.748	9.33E-07	Unclassified	-	-	K09014 - Fe-S cluster assembly protein SufB
-1.783	6.80E-10	Environmental Information Processing	Membrane transport	ABC transporters	K18891 - ATP-binding cassette, subfamily B, multidrug efflux pump patA, rscA, lmrC, satA
-1.874	7.75E-08	Metabolism	Carbohydrate Metabolism	Pentose and glucuronate interconversions	K00848 - rhamnulokinase rhaB [EC:2.7.1.5]
-1.881	1.44E-07	Unclassified	Unclassified: genetic information processing	Translation	K07571 - S1 RNA binding domain protein
-1.889	3.99E-07	Cellular Processes	Cell Growth and Death	Cell cycle - Caulobacter	K01358 - ATP-dependent Clp protease, protease subunit clpP [EC:3.4.21.92]
-1.947	1.06E-10	Unclassified	Protein families: signaling and cellular processes	Transporters	K10974 - cytosine permease codB
-1.950	2.88E-10	Metabolism	Metabolism of Other Amino Acids	Glutathione metabolism	K01919 - glutamate--cysteine ligase gshA [EC:6.3.2.2]
-1.989	1.74E-10	Metabolism	Amino Acid Metabolism	Cysteine and methionine metabolism	K00789 - S-adenosylmethionine synthetase metK [EC:2.5.1.6]
-1.997	2.76E-11	Metabolism	Lipid Metabolism	Fatty acid biosynthesis	K00059 - 3-oxoacyl-[acyl-carrier protein] reductase fabG [EC:1.1.1.100]
-2.005	2.07E-09	Environmental Information Processing	Signal Transduction	Two-component system	K01548 - K+-transporting ATPase ATPase C chain kdpC [EC:3.6.3.12]
-2.011	2.37E-09	Unclassified	Protein families: genetic information processing	Transfer RNA biogenesis	K07568 - S-adenosylmethionine:tRNA ribosyltransferase-isomerase queA
-2.068	4.46E-06	Unclassified	Protein families: signaling and cellular processes	Transporters	K02028 - polar amino acid transport system ATP-binding protein ABC.PA.A
-2.115	2.02E-06	Metabolism	Carbohydrate Metabolism	Starch and sucrose metabolism	K05350 - beta-glucosidase bglB [EC:3.2.1.21]
