# Supplementary material for: Unravelling the Molecular Mechanisms Underlying the Protective Effect of Lactate on the High-Pressure Resistance of Listeria monocytogenes
Source: Biomolecules. 2021 Apr 30;11(5):677. doi: 10.3390/biom11050677 (PMC8147161; doi:10.3390/biom11050677)
Supplement: Supplementary file 1 [file biomolecules-11-00677-s001.zip › biomolecules-1111984-proof-suppl/supplementary table 3.pdf]

Table S3. List of KEGG Orthology (KO) genes differentially (FDR<0.05) expressed in the *L. monocytogenes* CTC1034 strain in non-pressurized samples without and with lactate. Positive Log2 fold change indicate genes more abundant in samples with lactate.

Log2 Fold Change	FDR	KEGG annotation at level 1	KEGG annotation at level 2	KEGG pathway	KEGG Orthology (KO) genes
4.652	1.36E-02	Cellular Processes	Cell Motility	Flagellar assembly	K02400 - flagellar biosynthesis protein FlhA
4.394	1.46E-02	Environmental Information Processing	Signal Transduction	Two-component system	K02556 - chemotaxis protein MotA
4.358	4.19E-04	Environmental Information Processing	Signal Transduction	Two-component system	K00575 - chemotaxis protein methyltransferase CheR [EC:2.1.1.80]
4.268	1.70E-03	Metabolism	Carbohydrate Metabolism	Fructose and mannose metabolism	K02794 - PTS system, mannose-specific IIB component [EC:2.7.1.69], PTS-Man-EIIB, manX
4.225	5.76E-03	Metabolism	Metabolism of Cofactors and Vitamins	Thiamine metabolism	K03707 - thiaminase (transcriptional activator TenA) [EC:3.5.99.2]
4.207	5.76E-03	Metabolism	Carbohydrate Metabolism	Pentose and glucuronate interconversions	K00848 - rhamnulokinase rhaB [EC:2.7.1.5]
4.146	1.38E-02	Cellular Processes	Cell Motility	Flagellar assembly	K02389 - flagellar basal-body rod modification protein FlgD
4.106	1.29E-03	Metabolism	Metabolism of Cofactors and Vitamins	Thiamine metabolism	K00878 - hydroxyethylthiazole kinase thiM [EC:2.7.1.50]
4.099	4.78E-03	Brite Hierarchies	Protein families: signaling and cellular processes	Transporters	K11203 - PTS system, fructose-specific IIC-like component PTS-Fru2-EIIC
4.047	1.48E-02	Brite Hierarchies	Protein families: signaling and cellular processes	Bacterial motility proteins	K02404 - flagellar biosynthesis protein FlhF
4.001	1.84E-04	Metabolism	Energy Metabolism	Sulfur metabolism	K01760 - cystathionine beta-lyase metC [EC:4.4.1.8]
3.966	3.40E-03	Environmental Information Processing	Signal Transduction	Two-component system	K03407 - two-component system, chemotaxis family, sensor kinase CheA [EC:2.7.13.3]
3.869	1.76E-03	Metabolism	Metabolism of Cofactors and Vitamins	Porphyrin and chlorophyll metabolism	K02227 - adenosylcobinamide-phosphate synthase cbiB, cobD [EC:6.3.1.10]
3.856	3.89E-04	Metabolism	Carbohydrate Metabolism	Amino sugar and nucleotide sugar metabolism	K01183 - chitinase [EC:3.2.1.14]
3.816	4.16E-03	Cellular Processes	Cell Motility	Flagellar assembly	K02396 - flagellar hook-associated protein 1 FlgK
3.745	8.23E-04	Metabolism	Amino Acid Metabolism	Phenylalanine, tyrosine and tryptophan biosynthesis	K01657 - anthranilate synthase component I trpE [EC:4.1.3.27]
3.715	2.70E-02	Cellular Processes	Cell Motility	Flagellar assembly	K02392 - flagellar basal-body rod protein FlgG
3.647	1.35E-02	Cellular Processes	Cell Motility	Flagellar assembly	K02390 - flagellar hook protein FlgE
3.644	5.51E-03	Environmental Information Processing	Membrane Transport	ABC transporters	K17319 - putative aldouronate transport system permease protein lplB

3.619	1.53E-02	Environmental Information Processing	Signal Transduction	Two-component system	K03413 - two-component system, chemotaxis family, response regulator CheY
3.610	1.03E-02	Cellular Processes	Cell Motility	Bacterial chemotaxis	K02417 - flagellar motor switch protein FliN/FliY
3.573	2.17E-02	Cellular Processes	Cell Motility	Flagellar assembly	K02421 - flagellar biosynthetic protein FliR
3.568	8.30E-03	Metabolism	Carbohydrate Metabolism	Fructose and mannose metabolism	K02783 - PTS system, glucitol/sorbitol-specific IIC component, PTS-Gut-EIIC, srlA
3.548	5.34E-03	Environmental Information Processing	Membrane Transport	ABC transporters	K17320 - putative aldouronate transport system permease protein lplC
3.537	5.34E-03	Metabolism	Metabolism of Cofactors and Vitamins	Porphyrin and chlorophyll metabolism	K02191 - cobalt-precorrin-7 (C15)-methyltransferase cbiT [EC:2.1.1.196]
3.531	4.66E-03	Metabolism	Metabolism of Cofactors and Vitamins	Porphyrin and chlorophyll metabolism	K02226 - alpha-ribazole phosphatase cobC, phpB [EC:3.1.3.73]
3.526	4.69E-03	Metabolism	Carbohydrate Metabolism	Inositol phosphate metabolism	K03337 - 5-deoxy-glucuronate isomerase iolB [EC:5.3.1.-]
3.465	1.88E-02	Cellular Processes	Cell Motility	Flagellar assembly	K02388 - flagellar basal-body rod protein FlgC
3.337	3.13E-03	Environmental Information Processing	Signal Transduction	Two-component system	K07720 - two-component system, response regulator YesN
3.331	2.80E-02	Cellular Processes	Cell Motility	Bacterial chemotaxis	K02416 - flagellar motor switch protein FliM
3.310	1.13E-03	Metabolism	Amino Acid Metabolism	Arginine and proline metabolism	K00818 - acetylornithine aminotransferase argD [EC:2.6.1.11]
3.267	2.19E-02	Cellular Processes	Cell Motility	Flagellar assembly	K02409 - flagellar M-ring protein FliF
3.266	5.20E-03	Metabolism	Carbohydrate Metabolism	Fructose and mannose metabolism	K01813 - L-rhamnose isomerase rhaA [EC:5.3.1.14]
3.245	2.60E-03	Brite Hierarchies	Protein families: signaling and cellular processes	Transporters	K10974 - cytosine permease codB
3.183	1.29E-03	Brite Hierarchies	Protein families: signaling and cellular processes	Transporters	K08151 - MFS transporter, DHA1 family, tetracycline resistance protein tetA
3.159	1.57E-03	Unclassified	-	-	K09704 - uncharacterized protein
3.141	7.84E-03	Metabolism	Metabolism of Cofactors and Vitamins	Porphyrin and chlorophyll metabolism	K05936 - precorrin-4 C11-methyltransferase cobM, cbiF [EC:2.1.1.133]
3.131	5.61E-04	Brite Hierarchies	Protein families: signaling and cellular processes	Transporters	K02440 - glycerol uptake facilitator protein GLPF
3.127	1.81E-04	Metabolism	Carbohydrate Metabolism	Pentose phosphate pathway	K00615 - transketolase tktA, tktB [EC:2.2.1.1]
3.101	1.17E-02	Environmental Information Processing	Membrane Transport	ABC transporters	K15771 - putative arabinogalactan oligomer transport system permease protein ganP
3.088	2.70E-02	Cellular Processes	Cell Motility	Bacterial chemotaxis	K02410 - flagellar motor switch protein FliG
3.071	3.67E-03	Metabolism	Energy Metabolism	Oxidative phosphorylation	K02111 - F-type H+-transporting ATPase subunit alpha [EC:3.6.3.14]
3.071	1.04E-02	Environmental Information Processing	Membrane Transport	ABC transporters	K02007 - cobalt/nickel transport system permease protein cbiM

3.059	4.09E-03	Metabolism	Amino Acid Metabolism	Arginine and proline metabolism	K10536 - agmatine deiminase aguA [EC:3.5.3.12]
3.052	4.45E-02	Cellular Processes	Cell Motility	Flagellar assembly	K02408 - flagellar hook-basal body complex protein FliE
3.032	1.57E-03	Metabolism	Amino Acid Metabolism	Phenylalanine, tyrosine and tryptophan biosynthesis	K00766 - anthranilate phosphoribosyltransferase trpD [EC:2.4.2.18]
3.021	1.57E-03	Unclassified	-	-	K09703 - uncharacterized protein
3.018	3.69E-02	Cellular Processes	Cell Motility	Flagellar assembly	K02387 - flagellar basal-body rod protein FlgB
3.013	4.29E-02	Cellular Processes	Cell Motility	Flagellar assembly	K02411 - flagellar assembly protein FliH
2.979	2.74E-03	Metabolism	Carbohydrate Metabolism	Fructose and mannose metabolism	K00847 - fructokinase scrK [EC:2.7.1.4]
2.971	3.64E-03	Metabolism	Amino Acid Metabolism	Arginine and proline metabolism	K00620 - glutamate N-acetyltransferase / amino-acid N-acetyltransferase argJ [EC:2.3.1.35 2.3.1.1]
2.960	1.26E-02	Environmental Information Processing	Membrane Transport	ABC transporters	K15770 - putative arabinogalactan oligomer transport system substrate-binding protein cycB, ganO
2.959	1.85E-02	Metabolism	Metabolism of Cofactors and Vitamins	Porphyrin and chlorophyll metabolism	K02233 - adenosylcobinamide-GDP ribazoletransferase cobS, cobV [EC:2.7.8.26]
2.955	1.77E-02	Cellular Processes	Cell Motility	Flagellar assembly	K02412 - flagellum-specific ATP synthase fliI [EC:3.6.3.14]
2.943	8.88E-04	Metabolism	Nucleotide Metabolism	Pyrimidine metabolism	K02823 - dihydroorotate dehydrogenase electron transfer subunit pyrDII
2.924	3.77E-02	Cellular Processes	Cell Motility	Flagellar assembly	K02397 - flagellar hook-associated protein 3 FlgL
2.913	5.34E-03	Metabolism	Nucleotide Metabolism	Pyrimidine metabolism	K00609 - aspartate carbamoyltransferase catalytic subunit pyrB [EC:2.1.3.2]
2.906	2.46E-03	Metabolism	Lipid Metabolism	Glycerolipid metabolism	K13922 - propionaldehyde dehydrogenase pduP
2.886	9.02E-03	Metabolism	Carbohydrate Metabolism	Inositol phosphate metabolism	K03338 - 5-dehydro-2-deoxygluconokinase iolC [EC:2.7.1.92]
2.879	1.84E-04	Metabolism	Amino Acid Metabolism	Cysteine and methionine metabolism	K00547 - homocysteine S-methyltransferase mmuM [EC:2.1.1.10]
2.864	5.94E-03	Metabolism	Carbohydrate Metabolism	Starch and sucrose metabolism	K05349 - beta-glucosidase bglX [EC:3.2.1.21]
2.861	2.24E-02	Metabolism	Metabolism of Cofactors and Vitamins	Porphyrin and chlorophyll metabolism	K02188 - cobalt-precorrin-5B (C1)-methyltransferase cbiD [EC:2.1.1.195]
2.859	1.83E-02	Environmental Information Processing	Membrane Transport	ABC transporters	K17318 - putative aldouronate transport system substrate-binding protein lplA
2.839	3.64E-03	Metabolism	Amino Acid Metabolism	Phenylalanine, tyrosine and tryptophan biosynthesis	K01817 - phosphoribosylanthranilate isomerase trpF [EC:5.3.1.24]
2.810	4.66E-03	Metabolism	Nucleotide Metabolism	Pyrimidine metabolism	K01956 - carbamoyl-phosphate synthase small subunit carA [EC:6.3.5.5]
2.769	1.26E-02	Environmental Information Processing	Membrane Transport	ABC transporters	K02009 - cobalt transport protein cbiN
2.730	1.53E-02	Metabolism	Carbohydrate Metabolism	Galactose metabolism	K02774 - PTS system, galactitol-specific IIB component [EC:2.7.1.69], PTS-Gat-EIIB, gatB, sgcB
2.715	1.53E-02	Environmental Information Processing	Membrane Transport	ABC transporters	K16958 - L-cystine transport system permease protein tcyL
2.691	4.81E-03	Metabolism	Amino Acid Metabolism	Glycine, serine and threonine metabolism	K01695 - tryptophan synthase alpha chain trpA [EC:4.2.1.20]

2.637	3.66E-04	Metabolism	Carbohydrate Metabolism	Starch and sucrose metabolism	K05350 - beta-glucosidase bglB [EC:3.2.1.21]
2.632	7.13E-05	Metabolism	Lipid Metabolism	Glycerolipid metabolism	K00864 - glycerol kinase glpK [EC:2.7.1.30]
2.624	1.53E-02	Metabolism	Lipid Metabolism	Glycerolipid metabolism	K13919 - propanediol dehydratase medium subunit pduD [EC:4.2.1.28]
2.619	1.53E-03	Metabolism	Lipid Metabolism	Glycerolipid metabolism	K13921 - 1-propanol dehydrogenase pduQ
2.610	1.84E-02	Metabolism	Amino Acid Metabolism	Arginine and proline metabolism	K00145 - N-acetyl-gamma-glutamyl-phosphate reductase argC [EC:1.2.1.38]
2.609	1.02E-03	Metabolism	Nucleotide Metabolism	Pyrimidine metabolism	K17828 - dihydroorotate dehydrogenase (NAD+) catalytic subunit pyrDI
2.606	2.54E-03	Brite Hierarchies	Protein families: signaling and cellular processes	Transporters	K02027 - sugar transport system substrate-binding protein ABC.MS.S
2.545	2.48E-03	Environmental Information Processing	Membrane Transport	ABC transporters	K02038 - phosphate transport system permease protein pstA
2.540	1.85E-03	Metabolism	Carbohydrate Metabolism	Inositol phosphate metabolism	K03336 - 3D-(3,5/4)-trihydroxycyclohexane-1,2-dione hydrolase iolD [EC:3.7.1.-]
2.540	9.00E-03	Metabolism	Lipid Metabolism	Glycerolipid metabolism	K01699 - propanediol dehydratase large subunit pduC [EC:4.2.1.28]
2.527	2.55E-02	Unclassified	Unclassified: metabolism	Enzymes with EC numbers	K04844 - hypothetical glycosyl hydrolase ycjT [EC:3.2.1.-]
2.518	3.68E-02	Metabolism	Carbohydrate Metabolism	Fructose and mannose metabolism	K02781 - PTS system, glucitol/sorbitol-specific IIA component [EC:2.7.1.69], PTS-Gut-EIIA, srlB
2.488	2.89E-03	Metabolism	Nucleotide Metabolism	Pyrimidine metabolism	K01591 - orotidine-5'-phosphate decarboxylase pyrF [EC:4.1.1.23]
2.471	2.54E-02	Environmental Information Processing	Membrane Transport	ABC transporters	K15772 - putative arabinogalactan oligomer transport system permease protein ganQ
2.409	8.30E-03	Metabolism	Energy Metabolism	Nitrogen metabolism	K00265 - glutamate synthase (NADPH/NADH) large chain gltB [EC:1.4.1.13 1.4.1.14]
2.401	3.77E-02	Metabolism	Metabolism of Cofactors and Vitamins	Porphyrin and chlorophyll metabolism	K06042 - precorrin-8X methylmutase cobH-cbiC [EC:5.4.1.2]
2.391	9.80E-03	Unclassified	Unclassified: metabolism	Amino acid metabolism	K04024 - ethanolamine utilization protein EutJ
2.372	7.84E-03	Metabolism	Carbohydrate Metabolism	Propanoate metabolism	K13923 - phosphotransacylase pduL
2.340	2.63E-02	Metabolism	Metabolism of Cofactors and Vitamins	Porphyrin and chlorophyll metabolism	K03394 - precorrin-2/cobalt-factor-2 C20-methyltransferase cobI-cbiL [EC:2.1.1.130 2.1.1.151]
2.321	2.86E-02	Metabolism	Carbohydrate Metabolism	Fructose and mannose metabolism	K02769 - PTS system, fructose-specific IIB component [EC:2.7.1.69], PTS-Fru-EIIB, fruA
2.309	3.77E-02	Environmental Information Processing	Membrane Transport	ABC transporters	K16959 - L-cystine transport system permease protein, tcyM
2.296	1.35E-02	Metabolism	Metabolism of Cofactors and Vitamins	Thiamine metabolism	K00941 - hydroxymethylpyrimidine/phosphomethylpyrimidine kinase thiD [EC:2.7.1.49 2.7.4.7]
2.258	3.09E-02	Metabolism	Metabolism of Cofactors and Vitamins	Porphyrin and chlorophyll metabolism	K02190 - sirohydrochlorin cobaltochelatase cbiK [EC:4.99.1.3]
2.233	1.83E-02	Metabolism	Carbohydrate Metabolism	Pentose phosphate pathway	K01808 - ribose 5-phosphate isomerase B [EC:5.3.1.6], rpiB
2.230	3.30E-02	Metabolism	Energy Metabolism	Oxidative phosphorylation	K02114 - F-type H+-transporting ATPase subunit epsilon atpC [EC:3.6.3.14]
2.160	4.29E-02	Brite Hierarchies	Protein families: signaling and	Secretion system	K02236 - leader peptidase (prepilin peptidase) / N-methyltransferase comC

			cellular processes		
2.088	3.48E-02	Metabolism	Amino Acid Metabolism	Phenylalanine, tyrosine and tryptophan biosynthesis	K01658 - anthranilate synthase component II trpG [EC:4.1.3.27]
2.027	1.78E-02	Metabolism	Amino Acid Metabolism	Tyrosine metabolism	K00680 - uncharacterized N-acetyltransferase ytml [EC:2.3.1.-]
1.997	1.23E-02	Environmental Information Processing	Membrane Transport	Phosphotransferase system (PTS)	K02761 - PTS system, cellobiose-specific IIC component, PTS-Cel-EIIC, celB, chbC
1.962	3.81E-02	Metabolism	Metabolism of Cofactors and Vitamins	Nicotinate and nicotinamide metabolism	K00767 - nicotinate-nucleotide pyrophosphorylase (carboxylating) nadC [EC:2.4.2.19]
1.953	1.80E-02	Brite Hierarchies	Protein families: genetic information processing	Transcription factors	K03481 - RpiR family transcriptional regulator, glv operon transcriptional regulator glvR
1.953	3.34E-02	Metabolism	Glycan Biosynthesis and Metabolism	Other glycan degradation	K01191 - alpha-mannosidase MAN2C1 [EC:3.2.1.24]
1.910	1.57E-03	Environmental Information Processing	Membrane Transport	ABC transporters	K02016 - iron complex transport system substrate-binding protein
1.863	4.76E-02	Metabolism	Carbohydrate Metabolism	Starch and sucrose metabolism	K00690 - sucrose phosphorylase [EC:2.4.1.7]
1.851	1.83E-02	Metabolism	Metabolism of Cofactors and Vitamins	Thiamine metabolism	K00788 - thiamine-phosphate pyrophosphorylase thiE [EC:2.5.1.3]
1.821	1.97E-02	Cellular Processes	Cellular community - prokaryotes	Quorum sensing	K02034 - peptide/nickel transport system permease protein ABC.PE.P1
1.808	1.53E-02	Metabolism	Energy Metabolism	Sulfur metabolism	K00640 - serine O-acetyltransferase cysE [EC:2.3.1.30]
1.783	2.87E-02	Unclassified	Unclassified: metabolism	Amino acid metabolism	K04028 - ethanolamine utilization protein EutN
1.780	3.81E-02	Metabolism	Metabolism of Cofactors and Vitamins	Porphyrin and chlorophyll metabolism	K13542 - uroporphyrinogen III methyltransferase / synthase cobA-hemD [EC:2.1.1.107 4.2.1.75]
1.758	1.83E-02	Metabolism	Energy Metabolism	Sulfur metabolism	K00641 - homoserine O-acetyltransferase/O-succinyltransferase metX [EC:2.3.1.31 2.3.1.46]
1.736	1.97E-02	Environmental Information Processing	Membrane Transport	ABC transporters	K02036 - phosphate transport system ATP-binding protein pstB [EC:3.6.3.27]
1.657	2.70E-02	Metabolism	Carbohydrate Metabolism	Galactose metabolism	K02775 - PTS system, galactitol-specific IIC component, PTS-Gat-EIIC, gatC, sgcC
1.612	3.69E-02	Environmental Information Processing	Signal Transduction	Two-component system	K04751 - nitrogen regulatory protein P-II 1 glnB
1.609	6.20E-03	Genetic Information Processing	Replication and Repair	Base excision repair	K03648 - uracil-DNA glycosylase UNG [EC:3.2.2.27]
1.516	1.04E-02	Brite Hierarchies	Protein families: signaling and cellular processes	Transporters	K03321 - sulfate permease, SulP family TC.SULP
1.502	1.30E-02	Metabolism	Amino Acid Metabolism	Valine, leucine and isoleucine biosynthesis	K01687 - dihydroxy-acid dehydratase ilvD [EC:4.2.1.9]

1.481	2.17E-02	Metabolism	Metabolism of Cofactors and Vitamins	Porphyrin and chlorophyll metabolism	K16651 - L-threonine kinase pduX [EC:2.7.1.177]
1.442	4.25E-02	Metabolism	Carbohydrate Metabolism	Galactose metabolism	K01182 - oligo-1,6-glucosidase malL [EC:3.2.1.10]
1.376	3.54E-02	Unclassified	Unclassified: metabolism	Enzymes with EC numbers	K07047 - N-substituted formamide deformylase nfdA [EC:3.5.1.91]
1.369	2.47E-02	Metabolism	Nucleotide Metabolism	Purine metabolism	K01588 - 5-(carboxyamino)imidazole ribonucleotide mutase purE [EC:5.4.99.18]
1.352	1.23E-02	Genetic Information Processing	Folding, Sorting and Degradation	RNA degradation	K05592 - ATP-dependent RNA helicase DeaD [EC:3.6.4.13]
1.334	4.93E-02	Brite Hierarchies	Protein families: genetic information processing	Transfer RNA biogenesis	K06925 - tRNA threonylcarbamoyladenosine biosynthesis protein TsaE
1.168	3.95E-03	Cellular Processes	Cellular community - prokaryotes	Biofilm formation	K05946 - N-acetylglucosaminyldiphosphoundecaprenol N-acetyl-beta-D-mannosaminyltransferase tagA[EC:2.4.1.187]
1.108	4.82E-02	Metabolism	Nucleotide Metabolism	Purine metabolism	K23269 - phosphoribosylformylglycinamidine synthase subunit PurL [EC:6.3.5.3]
1.077	1.75E-02	Metabolism	Carbohydrate Metabolism	Pyruvate metabolism	K01006 - pyruvate,orthophosphate dikinase ppdk [EC:2.7.9.1]
1.058	1.25E-02	Metabolism	Lipid Metabolism	Glycerophospholipid metabolism	K03736 - ethanolamine ammonia-lyase small subunit eutC [EC:4.3.1.7]
1.051	2.70E-02	Unclassified	Unclassified: metabolism	Amino acid metabolism	K04026 - ethanolamine utilization protein EutL
1.040	5.20E-03	Metabolism	Metabolism of Cofactors and Vitamins	Pantothenate and CoA biosynthesis	K00077 - 2-dehydropantoate 2-reductase panE [EC:1.1.1.169]
1.009	2.61E-02	Environmental Information Processing	Membrane Transport	ABC transporters	K02000 - glycine betaine/proline transport system ATP-binding protein proV [EC:3.6.3.32]
0.902	1.03E-02	Brite Hierarchies	Protein families: genetic information processing	Transfer RNA biogenesis	K03177 - tRNA pseudouridine55 synthase [EC:5.4.99.25], truB, PUS4, TRUB1
0.843	3.03E-02	Metabolism	Carbohydrate Metabolism	Glycolysis / Gluconeogenesis	K01803 - triosephosphate isomerase (TIM) [EC:5.3.1.1], TPI, tpiA
0.747	7.64E-03	Metabolism	Metabolism of Cofactors and Vitamins	Ubiquinone and other terpenoid-quinone biosynthesis	K02549 - O-succinylbenzoate synthase menC [EC:4.2.1.113]
0.677	1.96E-02	Brite Hierarchies	Protein families: signaling and cellular processes	Transporters	K05020 - glycine betaine transporter opuD, betL
0.620	4.50E-02	Metabolism	Amino Acid Metabolism	Cysteine and methionine metabolism	K00789 - S-adenosylmethionine synthetase metK [EC:2.5.1.6]
0.571	4.65E-02	Brite Hierarchies	Protein families: signaling and cellular processes	Two-component system	K07183 - two-component system, response regulator / RNA-binding antiterminator nasT
0.515	4.75E-02	Brite Hierarchies	Protein families: signaling and cellular processes	Transporters	K03449 - MFS transporter, CP family, cyanate transporter MFS.CP
0.488	1.75E-02	Brite Hierarchies	Protein families:	Transporters	K01990 - ABC-2 type transport system ATP-binding protein

			signaling and cellular processes		
-0.276	3.73E-02	Unclassified	-	-	K07098 - uncharacterized protein
-0.377	1.88E-02	Metabolism	Metabolism of Cofactors and Vitamins	Thiamine metabolism	K04487 - cysteine desulfurase iscS [EC:2.8.1.7]
-0.411	2.23E-02	Unclassified	-	-	K07058 - membrane protein
-0.423	3.34E-02	Metabolism	Carbohydrate Metabolism	Butanoate metabolism	K00929 - butyrate kinase buk [EC:2.7.2.7]
-0.451	3.09E-02	Metabolism	Metabolism of Cofactors and Vitamins	Folate biosynthesis	K03752 - molybdenum cofactor guanylyltransferase mobA [EC:2.7.7.77]
-0.451	2.83E-02	Environmental Information Processing	Membrane Transport	ABC transporters	K16785 - energy-coupling factor transport system permease protein ecfT
-0.489	1.23E-02	Metabolism	Nucleotide Metabolism	Purine metabolism	K00951 - GTP pyrophosphokinase relA [EC:2.7.6.5]
-0.490	2.27E-02	Unclassified	-	-	K07023 - putative hydrolases of HD superfamily
-0.500	2.70E-02	Metabolism	Nucleotide Metabolism	Purine metabolism	K03784 - purine-nucleoside phosphorylase deoD [EC:2.4.2.1]
-0.515	2.70E-02	Brite Hierarchies	Protein families: metabolism	Peptidases and inhibitors	K01262 - Xaa-Pro aminopeptidase pepP [EC:3.4.11.9]
-0.546	1.36E-02	Brite Hierarchies	Protein families: genetic information processing	Transcription factors	K07738 - transcriptional repressor NrdR
-0.550	1.13E-02	Metabolism	Carbohydrate Metabolism	Pyruvate metabolism	K01759 - lactoylglutathione lyase gloA, GLO1 [EC:4.4.1.5]
-0.556	3.79E-02	Unclassified	-	-	K09963 - uncharacterized protein
-0.563	1.88E-02	Genetic Information Processing	Replication and Repair	Nucleotide excision repair	K03703 - excinuclease ABC subunit C, uvrC
-0.572	1.85E-02	Unclassified	Unclassified: metabolism	Enzymes with EC numbers	K00540 - F420H(2)-dependent quinone reductase fqr [EC:1.1.98.-]
-0.595	4.91E-02	Genetic Information Processing	Folding, Sorting and Degradation	Protein export	K12257 - SecD/SecF fusion protein
-0.597	2.05E-02	Genetic Information Processing	Folding, Sorting and Degradation	Protein export	K03070 - preprotein translocase subunit SecA
-0.601	3.64E-03	Brite Hierarchies	Protein families: genetic information processing	DNA replication proteins	K03346 - replication initiation and membrane attachment protein dnaB
-0.618	1.33E-03	Brite Hierarchies	Protein families: genetic information processing	Transfer RNA biogenesis	K07560 - D-aminoacyl-tRNA deacylase dtd
-0.643	6.23E-03	Metabolism	Nucleotide Metabolism	Purine metabolism	K02335 - DNA polymerase I polA [EC:2.7.7.7]
-0.648	2.84E-02	Metabolism	Nucleotide Metabolism	Purine metabolism	K00940 - nucleoside-diphosphate kinase ndk [EC:2.7.4.6]
-0.691	2.19E-02	Metabolism	Metabolism of Cofactors and Vitamins	Porphyrin and chlorophyll metabolism	K01772 - ferrochelatase hemH, FECH [EC:4.99.1.1]
-0.704	5.20E-03	Metabolism	Lipid Metabolism	Fatty acid biosynthesis	K00059 - 3-oxoacyl-[acyl-carrier protein] reductase FabG [EC:1.1.1.100]

-0.705	1.33E-03	Metabolism	Amino Acid Metabolism	Glycine, serine and threonine metabolism	K00283 - glycine dehydrogenase subunit 2 gcvPB [EC:1.4.4.2]
-0.720	3.40E-03	Brite Hierarchies	Protein families: signaling and cellular processes	Transporters	K06901 - putative MFS transporter, AGZA family, xanthine/uracil permease pbuG
-0.723	2.54E-03	Genetic Information Processing	Replication and Repair	Base excision repair	K01142 - exodeoxyribonuclease III xthA [EC:3.1.11.2]
-0.724	6.84E-04	Metabolism	Amino Acid Metabolism	Glycine, serine and threonine metabolism	K00282 - glycine dehydrogenase subunit 1 gcvPA [EC:1.4.4.2]
-0.728	1.57E-03	Brite Hierarchies	Protein families: metabolism	Protein kinases	K08884 - serine/threonine protein kinase, bacterial
-0.737	4.09E-02	Brite Hierarchies	Protein families: genetic information processing	DNA replication proteins	K03168 - DNA topoisomerase I topA [EC:5.6.2.1]
-0.745	2.63E-02	Genetic Information Processing	Folding, Sorting and Degradation	Protein processing in endoplasmic reticulum	K04079 - molecular chaperone HtpG
-0.754	2.25E-02	Metabolism	Metabolism of Cofactors and Vitamins	Pantothenate and CoA biosynthesis	K03525 - type III pantothenate kinase coaX [EC:2.7.1.33]
-0.768	1.94E-02	Unclassified	-	-	K07089 - uncharacterized protein
-0.784	3.67E-03	Brite Hierarchies	Protein families: metabolism	Peptidases and inhibitors	K01297 - muramoyltetrapeptide carboxypeptidase ldcA [EC:3.4.17.13]
-0.788	5.07E-03	Unclassified	-	-	K07045 - uncharacterized protein
-0.794	3.24E-03	Brite Hierarchies	Protein families: genetic information processing	Transcription factors	K03402 - transcriptional regulator of arginine metabolism argR, ahrC
-0.804	1.82E-02	Brite Hierarchies	Protein families: metabolism	Peptidases and inhibitors	K07284 - sortase A srtA [EC:3.4.22.70]
-0.810	1.30E-03	Metabolism	Metabolism of Terpenoids and Polyketides	Zeatin biosynthesis	K00791 - tRNA dimethylallyltransferase miaA [EC:2.5.1.75]
-0.812	7.84E-03	Genetic Information Processing	Replication and Repair	Homologous recombination	K03655 - ATP-dependent DNA helicase RecG [EC:3.6.4.12]
-0.827	1.60E-02	Metabolism	Glycan Biosynthesis and Metabolism	Peptidoglycan biosynthesis	K07260 - D-alanyl-D-alanine carboxypeptidase VanY [EC:3.4.16.4]
-0.841	2.54E-03	Unclassified	-	-	K05937 - uncharacterized protein
-0.855	1.02E-03	Metabolism	Metabolism of Cofactors and Vitamins	Riboflavin metabolism	K11753 - riboflavin kinase / FMN adenylyltransferase ribF [EC:2.7.1.26 2.7.7.2]
-0.867	1.23E-02	Unclassified	-	-	K06994 - putative drug exporter of the RND superfamily
-0.878	6.84E-04	Metabolism	Energy Metabolism	Oxidative phosphorylation	K02113 - F-type H+-transporting ATPase subunit delta atpH [EC:3.6.3.14]
-0.891	1.52E-02	Metabolism	Amino Acid Metabolism	Cysteine and methionine metabolism	K07173 - S-ribosylhomocysteine lyase luxS [EC:4.4.1.21]

-0.901	4.66E-03	Brite Hierarchies	Protein families: genetic information processing	Transcription factors	K10778 - AraC family transcriptional regulator, regulatory protein of adaptative response / methylated-DNA-[protein]-cysteine methyltransferase [EC:2.1.1.63], ada
-0.913	2.19E-02	Metabolism	Carbohydrate Metabolism	Pentose phosphate pathway	K00036 - glucose-6-phosphate 1-dehydrogenase [EC:1.1.1.49] G6PD, zwf
-0.915	3.66E-04	Unclassified	-	-	K07052 - uncharacterized protein
-0.918	1.57E-03	Metabolism	Metabolism of Cofactors and Vitamins	Thiamine metabolism	K06949 - ribosome biogenesis GTPase / thiamine phosphate phosphatase rsgA, engC
-0.919	8.78E-03	Metabolism	Lipid Metabolism	Glycerolipid metabolism	K08591 - acyl phosphate:glycerol-3-phosphate acyltransferase PlsY [EC:2.3.1.275]
-0.922	2.54E-03	Brite Hierarchies	Protein families: genetic information processing	Transfer RNA biogenesis	K03216 - tRNA (cytidine/uridine-2'-O-)-methyltransferase [EC:2.1.1.207] trmL, cspR
-0.931	1.69E-02	Brite Hierarchies	Protein families: genetic information processing	Messenger RNA biogenesis	K03698 - 3'-5' exoribonuclease cbf [EC:3.1.-.-]
-0.948	4.82E-02	Unclassified	-	-	K08986 - putative membrane protein ycgQ
-0.958	4.99E-06	Brite Hierarchies	Protein families: metabolism	Peptidases and inhibitors	K01273 - membrane dipeptidase DPEP
-0.959	1.11E-02	Metabolism	Amino Acid Metabolism	Valine, leucine and isoleucine degradation	K00167 - 2-oxoisovalerate dehydrogenase E1 component, beta subunit bkdA2 [EC:1.2.4.4]
-0.961	2.63E-02	Brite Hierarchies	Protein families: signaling and cellular processes	Transporters	K03284 - magnesium transporter corA
-0.962	2.42E-02	Environmental Information Processing	Membrane Transport	ABC transporters	K09693 - teichoic acid transport system ATP-binding protein tagH [EC:3.6.3.40]
-0.976	1.44E-02	Metabolism	Metabolism of Cofactors and Vitamins	Folate biosynthesis	K00950 - 2-amino-4-hydroxy-6-hydroxymethyldihydropteridine diphosphokinase folK [EC:2.7.6.3]
-0.979	1.51E-02	Metabolism	Amino Acid Metabolism	Valine, leucine and isoleucine degradation	K00166 - 2-oxoisovalerate dehydrogenase E1 component, alpha subunit bkdA1 [EC:1.2.4.4]
-0.983	2.02E-02	Metabolism	Carbohydrate Metabolism	Glycolysis / Gluconeogenesis	K02777 - PTS system, glucose-specific IIA component [EC:2.7.1.69], PTS-Glc-EIIA, crr
-0.985	3.30E-02	Metabolism	Energy Metabolism	Oxidative phosphorylation	K02108 - F-type H+-transporting ATPase subunit a [EC:3.6.3.14], atpB
-0.986	1.88E-02	Environmental Information Processing	Membrane Transport	ABC transporters	K18891 - ATP-binding cassette, subfamily B, multidrug efflux pump patA, rscA, lmrC, satA
-0.989	1.83E-02	Brite Hierarchies	Protein families: metabolism	Peptidoglycan biosynthesis and degradation proteins	K06078 - murein lipoprotein lpp
-0.996	3.89E-04	Brite Hierarchies	Protein families: metabolism	Enzymes with EC numbers	K01269 - aminopeptidase
-0.997	1.02E-03	Brite Hierarchies	Protein families: signaling and cellular processes	Transporters	K03282 - large conductance mechanosensitive channel mscL

-1.007	9.56E-03	Brite Hierarchies	Protein families: genetic information processing	DNA repair and recombination proteins	K03502 - DNA polymerase V umuC
-1.014	6.87E-03	Brite Hierarchies	Protein families: metabolism	Enzymes with EC numbers	K07305 - peptide-methionine (R)-S-oxide reductase msrB[EC:1.8.4.12]
-1.019	1.48E-02	Metabolism	Amino Acid Metabolism	Valine, leucine and isoleucine degradation	K09699 - 2-oxoisovalerate dehydrogenase E2 component (dihydrolipoyl transacylase) bkdB [EC:2.3.1.168]
-1.029	2.81E-02	Brite Hierarchies	Protein families: metabolism	Protein kinases	K04757 - serine/threonine-protein kinase RsbW [EC:2.7.11.1]
-1.032	1.36E-02	Metabolism	Carbohydrate Metabolism	Glycolysis / Gluconeogenesis	K00627 - pyruvate dehydrogenase E2 component (dihydrolipoamide acetyltransferase) [EC:2.3.1.12], aceF, pdhC, DLAT
-1.035	2.70E-02	Metabolism	Carbohydrate Metabolism	Pyruvate metabolism	K00656 - formate C-acetyltransferase pflD [EC:2.3.1.54]
-1.041	2.43E-02	Brite Hierarchies	Protein families: genetic information processing	Transcription factors	K01926 - redox-sensing transcriptional repressor rex
-1.043	6.84E-04	Metabolism	Nucleotide Metabolism	Pyrimidine metabolism	K00756 - pyrimidine-nucleoside phosphorylase pdp [EC:2.4.2.2]
-1.107	6.01E-06	Metabolism	Metabolism of Cofactors and Vitamins	Vitamin B6 metabolism	K08681 - glutamine amidotransferase pdxT, pdx2 [EC:2.6.-.-]
-1.112	2.54E-03	Metabolism	Carbohydrate Metabolism	Amino sugar and nucleotide sugar metabolism	K00820 - glucosamine--fructose-6-phosphate aminotransferase (isomerizing) glmS, GFPT [EC:2.6.1.16]
-1.113	1.05E-02	Brite Hierarchies	Protein families: genetic information processing	Chromosome and associated proteins	K03569 - rod shape-determining protein MreB and related proteins
-1.141	1.57E-03	Metabolism	Metabolism of Cofactors and Vitamins	Folate biosynthesis	K01633 - dihydroneopterin aldolase folB [EC:4.1.2.25]
-1.170	1.01E-02	Environmental Information Processing	Membrane Transport	ABC transporters	K18892 - ATP-binding cassette, subfamily B, multidrug efflux pump, patB, rscB, lmrC, satB
-1.199	1.57E-03	Metabolism	Energy Metabolism	Oxidative phosphorylation	K02112 - F-type H+-transporting ATPase subunit beta atpD [EC:3.6.3.14]
-1.222	1.20E-02	Metabolism	Carbohydrate Metabolism	Fructose and mannose metabolism	K02795 - PTS system, mannose-specific IIC component, PTS-Man-EIIC, manY
-1.291	3.64E-03	Metabolism	Carbohydrate Metabolism	Glycolysis / Gluconeogenesis	K00382 - dihydrolipoamide dehydrogenase DLD, lpd, pdhD [EC:1.8.1.4]
-1.296	4.68E-02	Metabolism	Carbohydrate Metabolism	Glycolysis / Gluconeogenesis	K00134 - glyceraldehyde 3-phosphate dehydrogenase GAPDH, gapA [EC:1.2.1.12]
-1.299	5.34E-03	Metabolism	Energy Metabolism	Oxidative phosphorylation	K02115 - F-type H+-transporting ATPase subunit gamma atpG [EC:3.6.3.14]
-1.301	1.29E-03	Metabolism	Amino Acid Metabolism	Lysine degradation	K00824 - D-alanine transaminase dat [EC:2.6.1.21]
-1.309	3.89E-04	Metabolism	Carbohydrate Metabolism	Pentose phosphate pathway	K01839 - phosphopentomutase deoB [EC:5.4.2.7]
-1.327	1.18E-06	Genetic Information Processing	Replication and Repair	DNA replication	K03469 - ribonuclease HI [EC:3.1.26.4]
-1.412	1.09E-02	Metabolism	Metabolism of other amino acids	D-Alanine metabolism	K03367 - D-alanine--poly(phosphoribitol) ligase subunit 1 DltA [EC:6.1.1.13]

-1.460	1.29E-04	Brite Hierarchies	Protein families: metabolism	Peptidases and inhibitors	K01372 - bleomycin hydrolase pepC
-1.466	1.62E-02	Metabolism	Metabolism of other amino acids	D-Alanine metabolism	K14188 - D-alanine--poly(phosphoribitol) ligase subunit 2 DltC [EC:6.1.1.13]
-1.483	1.48E-02	Environmental Information Processing	Signal Transduction	Two-component system	K03740 - D-alanine transfer protein DltD
-1.542	4.99E-02	Environmental Information Processing	Signal Transduction	Two-component system	K03739 - membrane protein involved in D-alanine export DltB
-1.632	4.41E-03	Metabolism	Lipid Metabolism	Fatty acid biosynthesis	K02078 - acyl carrier protein acpP
-1.914	2.44E-08	Metabolism	Carbohydrate Metabolism	Fructose and mannose metabolism	K02770 - PTS system, fructose-specific IIC component fruA
-1.980	1.84E-04	Metabolism	Carbohydrate Metabolism	Glycolysis / Gluconeogenesis	K04072 - acetaldehyde dehydrogenase / alcohol dehydrogenase adhe [EC:1.2.1.10 1.1.1.1]
-2.104	1.18E-06	Metabolism	Carbohydrate Metabolism	Fructose and mannose metabolism	K00882 - 1-phosphofructokinase fruK [EC:2.7.1.56]
