# Supplementary material for: Unravelling the Molecular Mechanisms Underlying the Protective Effect of Lactate on the High-Pressure Resistance of Listeria monocytogenes
Source: Biomolecules. 2021 Apr 30;11(5):677. doi: 10.3390/biom11050677 (PMC8147161; doi:10.3390/biom11050677)
Supplement: Supplementary file 1 [file biomolecules-11-00677-s001.zip › biomolecules-1111984-proof-suppl/supplementary table 4.pdf]

Table S4. List of KEGG Orthology (KO) genes differentially (FDR<0.05) expressed in the *L. monocytogenes* strain CTC1034 in pressurized samples without and with lactate. Negative Log2 fold change indicate genes less abundant in samples with lactate.

Log2 Fold Change	FDR	KEGG annotation at level 1	KEGG annotation at level 2	KEGG pathway	KEGG Orthology (KO) genes
- 1.668	1.47E-03	Metabolism	Carbohydrate Metabolism	Fructose and mannose metabolism	K00882 - 1-phosphofructokinase fruK [EC:2.7.1.56]
- 1.352	1.47E-03	Metabolism	Carbohydrate Metabolism	Fructose and mannose metabolism	K02770 - PTS system, fructose-specific IIC component, PTS-Fru-EIIC, fruA
- 1.100	1.48E-03	Metabolism	Nucleotide Metabolism	Pyrimidine metabolism	K00756 - pyrimidine-nucleoside phosphorylase pdp [EC:2.4.2.2]
