# Supplementary material for: Unravelling the Molecular Mechanisms Underlying the Protective Effect of Lactate on the High-Pressure Resistance of Listeria monocytogenes
Source: Biomolecules. 2021 Apr 30;11(5):677. doi: 10.3390/biom11050677 (PMC8147161; doi:10.3390/biom11050677)
Supplement: Supplementary file 1 [file biomolecules-11-00677-s001.zip › biomolecules-1111984-proof-suppl/supplementary table 5.pdf]

Table S5. List of KEGG Orthology (KO) genes differentially (FDR<0.05) expressed in the *L. monocytogenes* strain CTC1034 in samples with lactate non-pressurized and pressurized. Positive Log2 fold change indicate genes more abundant in pressurized samples.

Log2 Fold Change	FDR	KEGG annotation at level 1	KEGG annotation at level 2	KEGG pathway	KEGG Orthology (KO) genes
0.784	3.12E-02	Environmental Information Processing	Signal Transduction	Two-component system	K07646 - two-component system, OmpR family, sensor histidine kinase KdpD [EC:2.7.13.3]
0.725	3.96E-03	Metabolism	Carbohydrate Metabolism	Amino sugar and nucleotide sugar metabolism	K01443 - N-acetylglucosamine-6-phosphate deacetylase nagA [EC:3.5.1.25]
0.647	1.94E-02	Unclassified	Protein families: signaling and cellular processes	Transporters	K01990 - ABC-2 type transport system ATP-binding protein, ABC-2.A
-0.554	2.23E-02	Unclassified	Protein families: metabolism	Peptidases and inhibitors	K03797 - carboxyl-terminal processing protease ctpA, prc
-0.608	2.23E-02	Unclassified	-	-	K07058 - membrane protein
-0.917	1.94E-02	Unclassified	Protein families: signaling and cellular processes	Transporters	K06901 - putative MFS transporter, AGZA family, xanthine/uracil permease pbuG
-0.937	2.58E-02	Metabolism	Metabolism of Cofactors and Vitamins	Riboflavin metabolism	K11753 - riboflavin kinase / FMN adenylyltransferase ribF [EC:2.7.1.26 2.7.7.2]
-1.007	1.06E-02	Metabolism	Metabolism of Cofactors and Vitamins	Porphyrin and chlorophyll metabolism	K01599 - uroporphyrinogen decarboxylase HemE [EC:4.1.1.37]
-1.143	3.85E-03	Metabolism	Metabolism of Cofactors and Vitamins	Porphyrin and chlorophyll metabolism	K01772 - ferrochelatase HemH [EC:4.99.1.1]
-1.277	2.24E-02	Environmental Information Processing	Membrane Transport	ABC transporters	K09693 - teichoic acid transport system ATP-binding protein tagH [EC:3.6.3.40]
-1.767	2.92E-02	Genetic Information Processing	Translation	Ribosome	K02914 - large subunit ribosomal protein L34
