# Supplementary material for: Unravelling the Molecular Mechanisms Underlying the Protective Effect of Lactate on the High-Pressure Resistance of Listeria monocytogenes
Source: Biomolecules. 2021 Apr 30;11(5):677. doi: 10.3390/biom11050677 (PMC8147161; doi:10.3390/biom11050677)
Supplement: Supplementary file 1 [file biomolecules-11-00677-s001.zip › biomolecules-1111984-proof-suppl/supplementary table 6.pdf]

Table S6. List of KEGG Orthology (KO) genes differentially (FDR<0.05) expressed in CTC1034 *L. monocytogenes* strain throughout the comparison of control samples (non-exposed to lactate and non-pressurized) to samples exposed to lactate and pressurized. Positive Log2 fold change indicate genes more abundant in samples exposed to lactate and pressurized.

Log2 Fold Change	FDR	KEGG annotation at level 1	KEGG annotation at level 2	KEGG pathway	KEGG Orthology (KO) genes
3.951	9.73E-04	Cellular Processes	Cell Motility	Flagellar assembly	K02421 - flagellar biosynthetic protein FliR
3.660	3.63E-03	Metabolism	Energy Metabolism	Sulfur metabolism	K01760 - cystathionine beta-lyase [EC:4.4.1.8]
3.652	8.20E-03	Metabolism	Carbohydrate Metabolism	Fructose and mannose metabolism	K02794 - PTS system, mannose-specific IIB component [EC:2.7.1.69]
3.582	1.59E-03	Environmental Information Processing	Membrane Transport	ABC transporters	K17320 - putative aldouronate transport system permease protein, lplC
3.582	3.21E-02	Cellular Processes	Cell Motility	Flagellar assembly	K02400 - flagellar biosynthesis protein FlhA
3.503	1.08E-03	Environmental Information Processing	Membrane Transport	ABC transporters	K15771 - putative arabinogalactan oligomer transport system permease protein
3.498	1.31E-03	Environmental Information Processing	Signal Transduction	Two-component system	K07720 - two-component system, response regulator YesN
3.472	3.67E-03	Environmental Information Processing	Membrane Transport	ABC transporters	K17319 - putative aldourunate transport system permease protein, lplB
3.445	5.40E-03	Unclassified	Protein families: signaling and cellular processes	Bacterial motility proteins	K02404 - flagellar biosynthesis protein FlhF
3.436	4.31E-03	Metabolism	Amino Acid Metabolism	Phenylalanine, tyrosine and tryptophan biosynthesis	K01657 - anthranilate synthase component I [EC:4.1.3.27]
3.397	7.44E-03	Environmental Information Processing	Signal Transduction	Two-component system	K03413 - two-component system, chemotaxis family, response regulator CheY
3.386	8.20E-03	Unclassified	Protein families: signaling and cellular processes	Transporters	K11203 - fructose-like PTS system EIIC or EIIBC or EIIABC component
3.384	7.81E-03	Metabolism	Carbohydrate Metabolism	Pentose and glucuronate interconversions	K00848 - rhamnulokinase [EC:2.7.1.5]
3.360	1.86E-03	Metabolism	Carbohydrate Metabolism	Fructose and mannose metabolism	K01813 - L-rhamnose isomerase [EC:5.3.1.14]
3.355	3.58E-03	Metabolism	Carbohydrate Metabolism	Inositol phosphate metabolism	K03337 - 5-deoxy-glucuronate isomerase [EC:5.3.1.-]
3.340	1.65E-03	Metabolism	Metabolism of Cofactors and Vitamins	Porphyrin and chlorophyll metabolism	K02233 - adenosylcobinamide-GDP ribazoletransferase [EC:2.7.8.26]
3.332	1.52E-02	Metabolism	Metabolism of Cofactors and Vitamins	Thiamine metabolism	K03707 - thiaminase (transcriptional activator TenA) [EC:3.5.99.2]
3.327	3.58E-03	Metabolism	Carbohydrate Metabolism	Amino sugar and nucleotide sugar metabolism	K01183 - chitinase [EC:3.2.1.14]
3.316	7.67E-04	Environmental Information Processing	Membrane Transport	ABC transporters	K17318 - putatuve aldouronate transport system substrate-binding protein, lplA

3.301	4.01E-03	Metabolism	Carbohydrate Metabolism	Fructose and mannose metabolism	K02783 - PTS system, glucitol/sorbitol-specific IIC component
3.297	1.12E-02	Environmental Information Processing	Signal Transduction	Two-component system	K02556 - chemotaxis protein MotA
3.279	1.14E-02	Cellular Processes	Cell Motility	Flagellar assembly	K02389 - flagellar basal-body rod modification protein FlgD
3.267	4.75E-03	Cellular Processes	Cell Motility	Flagellar assembly	K02396 - flagellar hook-associated protein 1 FlgK
3.247	1.73E-02	Environmental Information Processing	Signal Transduction	Two-component system	K00575 - chemotaxis protein methyltransferase CheR [EC:2.1.1.80]
3.238	8.18E-04	Environmental Information Processing	Membrane Transport	ABC transporters	K15770 - putative arabinogalactan oligomer transport system substrate-binding protein
3.237	2.92E-03	Cellular Processes	Cell Motility	Flagellar assembly	K02411 - flagellar assembly protein FliH
3.237	4.31E-03	Metabolism	Metabolism of Cofactors and Vitamins	Porphyrin and chlorophyll metabolism	K02226 - alpha-ribazole phosphatase [EC:3.1.3.73]
3.230	1.06E-03	Unclassified	Protein families: signaling and cellular processes	Secretion system	K02236 - Leader peptidase (prepilin peptidase) / N-methyltransferase [EC: 3.4.23.43 2.1.1.-], ComC
3.212	2.60E-03	Cellular Processes	Cell Motility	Bacterial chemotaxis	K02410 - flagellar motor switch protein FliG
3.210	6.14E-03	Cellular Processes	Cell Motility	Flagellar assembly	K02390 - flagellar hook protein FlgE
3.205	2.54E-03	Metabolism	Nucleotide Metabolism	Pyrimidine metabolism	K00609 - aspartate carbamoyltransferase catalytic subunit [EC:2.1.3.2]
3.203	3.15E-03	Unclassified	Protein families: signaling and cellular processes	Transporters	K10974 - cytosine permease, codB
3.196	4.33E-03	Cellular Processes	Cell Motility	Bacterial chemotaxis	K02416 - flagellar motor switch protein FliM
3.192	2.71E-03	Metabolism	Amino Acid Metabolism	Phenylalanine, tyrosine and tryptophan biosynthesis	K01817 - phosphoribosylanthranilate isomerase [EC:5.3.1.24]
3.187	7.90E-03	Cellular Processes	Cell Motility	Flagellar assembly	K02392 - flagellar basal-body rod protein FlgG
3.183	5.22E-03	Environmental Information Processing	Signal Transduction	Two-component system	K03407 - two-component system, chemotaxis family, sensor kinase CheA [EC:2.7.13.3]
3.178	5.61E-03	Cellular Processes	Cell Motility	Bacterial chemotaxis	K02417 - flagellar motor switch protein FliN/FliY
3.176	4.11E-03	Cellular Processes	Cell Motility	Flagellar assembly	K02387 - flagellar basal-body rod protein FlgB
3.173	3.58E-03	Cellular Processes	Cell Motility	Flagellar assembly	K02409 - flagellar M-ring protein FliF
3.169	2.16E-03	Cellular Processes	Cell Motility	Flagellar assembly	K02397 - flagellar hook-associated protein 3 FlgL
3.164	2.07E-03	Metabolism	Nucleotide Metabolism	Pyrimidine metabolism	K01956 - carbamoyl-phosphate synthase small subunit [EC:6.3.5.5]
3.153	4.76E-03	Metabolism	Carbohydrate Metabolism	Fructose and mannose metabolism	K02781 - PTS system, glucitol/sorbitol-specific IIA component [EC:2.7.1.69]
3.127	1.10E-03	Environmental Information Processing	Signal Transduction	Two-component system	K01546 - K+-transporting ATPase ATPase A chain [EC:3.6.3.12]
3.119	3.54E-03	Metabolism	Carbohydrate Metabolism	Fructose and mannose metabolism	K00847 - fructokinase [EC:2.7.1.4]

3.075	8.48E-04	Unclassified	Protein families: signaling and cellular processes	Transporters	K02027 - multiple sugar transport system substrate-binding protein
3.070	5.03E-03	Environmental Information Processing	Membrane Transport	ABC transporters	K02007 - cobalt/nickel transport system permease protein
3.058	3.15E-03	Metabolism	Amino Acid Metabolism	Phenylalanine, tyrosine and tryptophan biosynthesis	K00766 - anthranilate phosphoribosyltransferase [EC:2.4.2.18]
3.047	3.63E-03	Metabolism	Carbohydrate Metabolism	Pentose phosphate pathway	K00615 - transketolase [EC:2.2.1.1]
3.043	4.06E-03	Unclassified	Protein families: signaling and cellular processes	Transporters	K08151 - MFS transporter, DHA1 family, tetracycline resistance protein, tetA
3.034	1.86E-03	Metabolism	Amino Acid Metabolism	Glycine, serine and threonine metabolism	K01695 - tryptophan synthase alpha chain [EC:4.2.1.20]
3.022	3.14E-03	Metabolism	Lipid Metabolism	Glycerolipid metabolism	K13922 - propionaldehyde dehydrogenase
3.011	4.56E-03	Metabolism	Lipid Metabolism	Glycerolipid metabolism	K13919 - propanediol dehydratase medium subunit [EC:4.2.1.28]
3.003	1.02E-02	Metabolism	Metabolism of Cofactors and Vitamins	Porphyrin and chlorophyll metabolism	K02191 - cobalt-precorrin-7 (C15)-methyltransferase [EC:2.1.1.196]
3.000	2.21E-02	Metabolism	Metabolism of Cofactors and Vitamins	Thiamine metabolism	K00878 - hydroxyethylthiazole kinase [EC:2.7.1.50]
2.998	1.39E-02	Cellular Processes	Cell Motility	Flagellar assembly	K02388 - flagellar basal-body rod protein FlgC
2.993	2.92E-03	Metabolism	Energy Metabolism	Oxidative phosphorylation	K02111 - F-type H+-transporting ATPase subunit alpha [EC:3.6.3.14]
2.993	5.49E-03	Metabolism	Carbohydrate Metabolism	Galactose metabolism	K02774 - PTS system, galactitol-specific IIB component [EC:2.7.1.69]
2.967	5.03E-03	Unclassified	Protein families: signaling and cellular processes	Transporters	K02440 - glycerol uptake facilitator protein, GLPF
2.954	3.37E-03	Metabolism	Nucleotide Metabolism	Pyrimidine metabolism	K02823 - dihydroorotate dehydrogenase electron transfer subunit, pyrDII
2.921	3.58E-03	Metabolism	Metabolism of Cofactors and Vitamins	Porphyrin and chlorophyll metabolism	K02188 - cobalt-precorrin-5B (C1)-methyltransferase [EC:2.1.1.195]
2.915	1.52E-03	Environmental Information Processing	Membrane Transport	ABC transporters	K15772 - putative arabinogalactan oligomer transport system permease protein
2.910	4.55E-03	Metabolism	Amino Acid Metabolism	Arginine and proline metabolism	K10536 - agmatine deiminase [EC:3.5.3.12]
2.910	1.28E-03	Unclassified	Unclassified: metabolism	Enzymes with EC numbers	K04844 - hypothetical glycosyl hydrolase [EC: 3.2.1.-], ycjT
2.908	2.92E-03	Metabolism	Carbohydrate Metabolism	Starch and sucrose metabolism	K05349 - beta-glucosidase [EC:3.2.1.21]
2.907	3.42E-03	Metabolism	Amino Acid Metabolism	Cysteine and methionine metabolism	K00547 - homocysteine S-methyltransferase [EC:2.1.1.10]
2.907	7.62E-03	Unclassified	Not included in Pathway or Brite	Poorly characterized	K09704 - uncharacterized protein

2.894	1.14E-02	Environmental Information Processing	Membrane Transport	ABC transporters	K02009 - cobalt transport protein
2.887	2.92E-03	Metabolism	Lipid Metabolism	Glycerolipid metabolism	K00864 - glycerol kinase [EC:2.7.1.30]
2.885	4.55E-03	Unclassified	Not included in Pathway or Brite	Poorly characterized	K09703 - uncharacterized protein
2.842	2.71E-03	Metabolism	Carbohydrate Metabolism	Starch and sucrose metabolism	K05350 - beta-glucosidase [EC:3.2.1.21]
2.842	9.28E-04	Metabolism	Energy Metabolism	Nitrogen metabolism	K00265 - glutamate synthase (NADPH/NADH) large chain [EC:1.4.1.13 1.4.1.14]
2.837	7.29E-03	Metabolism	Amino Acid Metabolism	Arginine and proline metabolism	K00818 - acetylornithine aminotransferase [EC:2.6.1.11]
2.805	4.55E-03	Cellular Processes	Cell Motility	Flagellar assembly	K02412 - flagellum-specific ATP synthase [EC:3.6.3.14]
2.796	5.12E-03	Metabolism	Carbohydrate Metabolism	Inositol phosphate metabolism	K03338 - 5-dehydro-2-deoxygluconokinase [EC:2.7.1.92]
2.773	5.03E-03	Metabolism	Metabolism of Cofactors and Vitamins	Porphyrin and chlorophyll metabolism	K02190 - sirohydrochlorin cobaltochelatase [EC:4.99.1.3]
2.765	4.64E-03	Metabolism	Metabolism of Cofactors and Vitamins	Porphyrin and chlorophyll metabolism	K03394 - precorrin-2/cobalt-factor-2 C20-methyltransferase [EC:2.1.1.130 2.1.1.151]
2.749	5.46E-03	Metabolism	Nucleotide Metabolism	Pyrimidine metabolism	K01591 - orotidine-5'-phosphate decarboxylase [EC:4.1.1.23]
2.747	1.95E-02	Metabolism	Metabolism of Cofactors and Vitamins	Porphyrin and chlorophyll metabolism	K02227 - adenosylcobinamide-phosphate synthase [EC:6.3.1.10]
2.743	4.55E-03	Unclassified	Protein families: genetic information processing	Ribosome biogenesis	K00783 - 23S rRNA (pseudouridine1915-N3)-methyltransferase [EC: 2.1.1.177], rlmH
2.735	4.01E-03	Metabolism	Carbohydrate Metabolism	Propanoate metabolism	K13923 - phosphotransacylase
2.725	3.54E-03	Metabolism	Lipid Metabolism	Glycerolipid metabolism	K13921 - 1-propanol dehydrogenase
2.725	4.01E-03	Metabolism	Lipid Metabolism	Glycerolipid metabolism	K01699 - propanediol dehydratase large subunit [EC:4.2.1.28]
2.723	2.92E-03	Environmental Information Processing	Membrane Transport	ABC transporters	K02038 - phosphate transport system permease protein
2.704	3.18E-03	Metabolism	Carbohydrate Metabolism	Fructose and mannose metabolism	K02769 - PTS system, fructose-specific IIB component [EC:2.7.1.69]
2.684	2.31E-03	Unclassified	Unclassified: metabolism	Amino acid metabolism	K04024 - ethanolamine utilization protein EutJ
2.664	4.30E-03	Environmental Information Processing	Membrane Transport	ABC transporters	K16958 - L-cystine transport system permease protein, tcyL
2.653	3.54E-03	Unclassified	Unclassified: genetic information processing	Replication and repair	K03630 - DNA repair protein RadC
2.649	1.14E-02	Metabolism	Metabolism of Cofactors and Vitamins	Porphyrin and chlorophyll metabolism	K05936 - precorrin-4 C11-methyltransferase [EC:2.1.1.133]
2.643	5.55E-03	Metabolism	Nucleotide Metabolism	Pyrimidine metabolism	K17828 - dihydroorotate dehydrogenase (NAD+) catalytic subunit [EC:1.3.1.14], pyrDI
2.629	6.83E-03	Metabolism	Metabolism of Cofactors and Vitamins	Porphyrin and chlorophyll metabolism	K06042 - precorrin-8X methylmutase [EC:5.4.1.2]

2.627	1.59E-03	Environmental Information Processing	Signal Transduction	Two-component system	K04751 - nitrogen regulatory protein P-II 1
2.623	7.59E-04	Environmental Information Processing	Membrane Transport	Phosphotransferase system (PTS)	K02761 - PTS system, cellobiose-specific IIC component
2.611	1.48E-02	Metabolism	Amino Acid Metabolism	Arginine and proline metabolism	K00620 - glutamate N-acetyltransferase / amino-acid N-acetyltransferase [EC:2.3.1.35 2.3.1.1]
2.550	2.34E-03	Unclassified	Protein families: signaling and cellular processes	Transporters	K02025 - multiple sugar transport system permease protein
2.544	5.49E-03	Metabolism	Carbohydrate Metabolism	Inositol phosphate metabolism	K03336 - 3D-(3,5/4)-trihydroxycyclohexane-1,2-dione hydrolase [EC:3.7.1.-]
2.522	6.57E-03	Metabolism	Carbohydrate Metabolism	Pentose phosphate pathway	K01808 - ribose 5-phosphate isomerase B [EC: 5.3.1.6], rpiB
2.441	1.34E-02	Environmental Information Processing	Membrane Transport	ABC transporters	K16959 - L-cystine transport system permease protein, tcyM
2.433	5.46E-03	Metabolism	Metabolism of Cofactors and Vitamins	Porphyrin and chlorophyll metabolism	K05934 - precorrin-3B C17-methyltransferase [EC:2.1.1.131]
2.416	1.21E-02	Metabolism	Metabolism of Cofactors and Vitamins	Thiamine metabolism	K00941 - hydroxymethylpyrimidine/phosphomethylpyrimidine kinase [EC:2.7.1.49 2.7.4.7]
2.407	1.21E-02	Unclassified	Protein families: signaling and cellular processes	Secretion system	K02246 - competence protein ComGD
2.392	2.41E-02	Metabolism	Amino Acid Metabolism	Arginine and proline metabolism	K00145 - N-acetyl-gamma-glutamyl-phosphate reductase [EC:1.2.1.38]
2.390	1.86E-03	Unclassified	Protein families: metabolism	Peptidases and inhibitors	k08600 - sortase B [EC:3.4.22.71], strB
2.389	1.39E-03	Metabolism	Carbohydrate Metabolism	Ascorbate and aldarate metabolism	K02822 - PTS system, ascorbate-specific IIB component [EC:2.7.1.69]
2.380	3.15E-03	Metabolism	Amino Acid Metabolism	Phenylalanine, tyrosine and tryptophan biosynthesis	K01658 - anthranilate synthase component II [EC:4.1.3.27]
2.377	5.76E-04	Unclassified	Protein families: signaling and cellular processes	Transporters	K02026 - multiple sugar transport system permease protein
2.372	1.51E-02	Metabolism	Energy Metabolism	Oxidative phosphorylation	K02114 - F-type H+-transporting ATPase subunit epsilon [EC:3.6.3.14]
2.367	3.91E-03	Metabolism	Metabolism of Cofactors and Vitamins	Nicotinate and nicotinamide metabolism	K00767 - nicotinate-nucleotide pyrophosphorylase (carboxylating) [EC:2.4.2.19]
2.362	9.73E-04	Metabolism	Amino Acid Metabolism	Valine, leucine and isoleucine biosynthesis	K01687 - dihydroxy-acid dehydratase [EC:4.2.1.9]
2.349	3.15E-03	Metabolism	Glycan Biosynthesis and Metabolism	Other glycan degradation	K01191 - alpha-mannosidase [EC:3.2.1.24]
2.331	1.54E-02	Cellular Processes	Cell Motility	Flagellar assembly	K02419 - flagellar biosynthetic protein FliP
2.317	3.54E-03	Metabolism	Carbohydrate Metabolism	Starch and sucrose metabolism	K00690 - sucrose phosphorylase [EC:2.4.1.7]

2.309	1.24E-03	Environmental Information Processing	Membrane Transport	ABC transporters	K11051 - multidrug/hemolysin transport system permease protein
2.308	6.09E-03	Metabolism	Metabolism of Cofactors and Vitamins	Porphyrin and chlorophyll metabolism	K13542 - uroporphyrinogen III methyltransferase / synthase [EC:2.1.1.107 4.2.1.75]
2.285	8.55E-03	Unclassified	Protein families: genetic information processing	Transcription factors	K03481 - RpiR family transcriptional regulator, glv operon transcriptional regulator, glvR
2.282	2.83E-02	Unclassified	Not included in Pathway or Brite	Poorly characterized	K09770 - uncharacterized protein
2.275	1.59E-03	Metabolism	Carbohydrate Metabolism	Galactose metabolism	K02775 - PTS system, galactitol-specific IIC component
2.272	3.37E-02	Unclassified	Protein families: signaling and cellular processes	Secretion system	K02249 - competence protein ComGG
2.266	2.80E-02	Unclassified	Protein families: signaling and cellular processes	Secretion system	K02247 - competence protein ComGE
2.264	8.55E-03	Unclassified	Unclassified: metabolism	Enzymes with EC numbers	K00680 - uncharacterized N-acetyltransferase [EC:2.3.1.-], ytmI
2.261	2.82E-03	Unclassified	Protein families: metabolism	Peptidases and inhibitors	K05995 - dipeptidase E [EC:3.4.13.21]
2.232	5.16E-03	Metabolism	Metabolism of Cofactors and Vitamins	Folate biosynthesis	K08310 - dihydroneopterin triphosphate diphosphatase [EC:3.6.1.67], nudB, ntpA
2.226	2.86E-03	Metabolism	Carbohydrate Metabolism	Galactose metabolism	K01182 - oligo-1,6-glucosidase [EC:3.2.1.10]
2.200	5.16E-03	Unclassified	Unclassified: metabolism	Enzymes with EC numbers	K09935 - N-glycosidase YbiA [EC:3.2.2.-], ybiA
2.194	1.86E-03	Unclassified	Protein families: signaling and cellular processes	Transporters	K08170 - MFS transporter, DHA2 family, multidrug resistance protein, norB, norC
2.186	3.76E-03	Environmental Information Processing	Membrane Transport	ABC transporters	K02016 - iron complex transport system substrate-binding protein
2.175	3.63E-03	Environmental Information Processing	Membrane Transport	ABC transporters	K02036 - phosphate transport system ATP-binding protein [EC:3.6.3.27]
2.168	1.52E-02	Metabolism	Metabolism of Cofactors and Vitamins	Porphyrin and chlorophyll metabolism	K02231 - adenosylcobinamide kinase / adenosylcobinamide-phosphate guanylyltransferase [EC:2.7.1.156 2.7.7.62]
2.168	6.53E-03	Cellular Processes	Cellular community-prokaryotes	Quorum sensing	K02034 - peptide/nickel transport system permease protein
2.133	1.04E-03	Environmental Information Processing	Membrane Transport	Phosphotransferase system (PTS)	K02757 - PTS system, beta-glucosides-specific IIC component
2.123	4.76E-03	Metabolism	Carbohydrate Metabolism	Galactose metabolism	K02773 - PTS system, galactitol-specific IIA component [EC:2.7.1.69]
2.105	7.97E-03	Metabolism	Metabolism of Cofactors and Vitamins	Thiamine metabolism	K00788 - thiamine-phosphate pyrophosphorylase [EC:2.5.1.3]

2.096	4.95E-05	Metabolism	Carbohydrate Metabolism	Fructose and mannose metabolism	K02798 - PTS system, mannitol-specific IIA component cmtB [EC:2.7.1.69]
2.083	8.50E-03	Metabolism	Metabolism of Cofactors and Vitamins	Porphyrin and chlorophyll metabolism	K05895 - precorrin-6X reductase [EC:1.3.1.54]
2.055	2.92E-03	Metabolism	Amino Acid Metabolism	Valine, leucine and isoleucine biosynthesis	K00053 - ketol-acid reductoisomerase [EC:1.1.1.86]
2.052	4.55E-03	Metabolism	Carbohydrate Metabolism	Fructose and mannose metabolism	K02768 - PTS system, fructose-specific IIA component [EC:2.7.1.69]
2.049	8.32E-03	Environmental Information Processing	Membrane Transport	ABC transporters	K02008 - cobalt/nickel transport system permease protein
2.032	6.83E-03	Unclassified	Protein families: signaling and cellular processes	Transporters	K11741 - quaternary ammonium compound-resistance protein SugE
2.019	7.96E-03	Metabolism	Energy Metabolism	Sulfur metabolism	K00641 - homoserine O-acetyltransferase [EC:2.3.1.31]
2.014	6.80E-03	Metabolism	Carbohydrate Metabolism	Fructose and mannose metabolism	K00008 - L-iditol 2-dehydrogenase [EC:1.1.1.14]
1.986	1.53E-02	Metabolism	Metabolism of Cofactors and Vitamins	Porphyrin and chlorophyll metabolism	K02224 - cobyrinic acid a,c-diamide synthase [EC:6.3.5.9 6.3.5.11]
1.972	1.91E-03	Unclassified	Protein families: signaling and cellular processes	Secretion system	K02244 - competence protein ComGB
1.958	9.38E-03	Metabolism	Carbohydrate Metabolism	Starch and sucrose metabolism	K00691 - maltose phosphorylase [EC:2.4.1.8]
1.946	4.17E-03	Metabolism	Carbohydrate Metabolism	C5-Branched dibasic acid metabolism	K01704 - 3-isopropylmalate/(R)-2-methylmalate dehydratase small subunit [EC:4.2.1.33 4.2.1.35]
1.930	4.21E-03	Genetic Information Processing	Replication and Repair	Base excision repair	K03648 - uracil-DNA glycosylase [EC:3.2.2.27]
1.928	2.82E-03	Metabolism	Nucleotide Metabolism	Purine metabolism	K01588 - 5-(carboxyamino)imidazole ribonucleotide mutase [EC:5.4.99.18]
1.915	1.89E-02	Metabolism	Metabolism of Cofactors and Vitamins	Nicotinate and nicotinamide metabolism	K03517 - quinolinate synthase [EC:2.5.1.72]
1.909	9.34E-03	Metabolism	Amino Acid Metabolism	Arginine and proline metabolism	K01470 - creatinine amidohydrolase [EC:3.5.2.10]
1.892	1.51E-02	Unclassified	Unclassified: metabolism	Enzymes with EC numbers	K18673 - beta-glucoside kinase [EC:2.7.1.85], bglK
1.886	5.05E-03	Metabolism	Amino Acid Metabolism	Glycine, serine and threonine metabolism	K01754 - threonine dehydratase [EC:4.3.1.19]
1.874	1.95E-02	Unclassified	Protein families: genetic information processing	Ribosome biogenesis	K00563 - 23S rRNA (guanine745-N1)-methyltransferase [EC:2.1.1.187], rlmA1
1.858	2.68E-02	Metabolism	Energy Metabolism	Sulfur metabolism	K00640 - serine O-acetyltransferase [EC:2.3.1.30]
1.847	4.01E-03	Metabolism	Carbohydrate Metabolism	Ascorbate and aldarate metabolism	K03475 - PTS system, ascorbate-specific IIC component
1.845	3.67E-03	Environmental Information Processing	Membrane Transport	ABC transporters	K02020 - molybdate transport system substrate-binding protein
1.809	4.40E-03	Environmental Information Processing	Membrane Transport	ABC transporters	K11071 - spermidine/putrescine transport system permease protein

1.796	2.17E-02	Unclassified	Not included in Pathway or Brite	Poorly characterized	K07048 - phosphotriesterase-related protein
1.765	6.62E-03	Metabolism	Carbohydrate Metabolism	Fructose and mannose metabolism	K01840 - phosphomannomutase [EC:5.4.2.8]
1.745	1.67E-02	Metabolism	Metabolism of Cofactors and Vitamins	Porphyrin and chlorophyll metabolism	K16651 - L-threonine kinase [EC: 2.7.1.177], pduX
1.735	1.54E-02	Metabolism	Amino Acid Metabolism	Histidine metabolism	K02501 - glutamine amidotransferase [EC:2.4.2.-]
1.720	4.55E-03	Metabolism	Carbohydrate Metabolism	Fructose and mannose metabolism	K01628 - L-fuculose-phosphate aldolase [EC:4.1.2.17]
1.718	4.01E-03	Unclassified	Protein families: genetic information processing	Transcription factors	K02538 - activator of the mannose opeon, transcriptional antiterminator, manR
1.679	1.15E-02	Metabolism	Nucleotide Metabolism	Purine metabolism	K01952 - phosphoribosylformylglycinamidine synthase [EC:6.3.5.3]
1.643	1.14E-02	Environmental Information Processing	Membrane Transport	ABC transporters	K02006 - cobalt/nickel transport system ATP-binding protein
1.640	7.44E-03	Unclassified	Protein families: genetic information processing	DNA repair and recombinationn proteins	K03546 - DNA repair protein SbcC/Rad50
1.631	1.05E-02	Environmental Information Processing	Membrane Transport	ABC transporters	K02015 - iron complex transport system permease protein
1.624	3.54E-03	Environmental Information Processing	Membrane Transport	ABC transporters	K09815 - zinc transport system substrate-binding protein
1.623	1.31E-02	Metabolism	Amino Acid Metabolism	Valine, leucine and isoleucine biosynthesis	K00052 - 3-isopropylmalate dehydrogenase [EC:1.1.1.85]
1.622	2.60E-02	Unclassified	Unclassified: metabolism	Enzymes with EC numbers	K07047 - N-substituted formamide deformylase [EC:3.5.1.91], ndfA
1.622	1.05E-02	Unclassified	Protein families: genetic information processing	Transcription factors	K03491 - lichenan operon transcriptional antiterminator, licR
1.620	2.66E-02	Unclassified	Protein families: signaling and cellular processes	Transporters	K03321 - sulfate permease, SulP family
1.596	3.59E-04	Unclassified	Protein families: genetic information processing	Transcription factors	K09681 - LysR family transcriptional regulator, transcription glutamate synthase operon, gltC
1.590	1.54E-02	Environmental Information Processing	Signal Transduction	Two-component system	K02406 - flagellin, fliC
1.580	1.78E-02	Unclassified	Protein families: genetic information processing	Transfer RNA biogenesis	K06925 - tRNA threonylcarbamoyladenosine biosynthesis protein TsaE
1.575	5.05E-03	Metabolism	Carbohydrate Metabolism	Pyruvate metabolism	K01006 - pyruvate,orthophosphate dikinase [EC:2.7.9.1]

1.557	9.34E-03	Metabolism	Metabolism of Cofactors and Vitamins	Folate biosynthesis	K03635 - molybdopterin synthase catalytic subunit [EC:2.-.-.-]
1.513	1.45E-02	Unclassified	Protein families: genetic information processing	DNA repair and recombinationn proteins	K03547 - DNA repair protein SbcD/Mre11
1.487	4.13E-03	Environmental Information Processing	Signal Transduction	Two-component system	K01548 - K+-transporting ATPase ATPase C chain [EC:3.6.3.12]
1.485	1.50E-02	Environmental Information Processing	Membrane Transport	ABC transporters	K11072 - spermidine/putrescine transport system ATP-binding protein [EC:3.6.3.31]
1.470	1.28E-02	Metabolism	Nucleotide Metabolism	Purine metabolism	K23269 - phosphoribosylformylglycinamidine synthase subunit PurL [EC: 6.3.5.3]
1.469	4.72E-02	Metabolism	Nucleotide Metabolism	Purine metabolism	K11175 - phosphoribosylglycinamide formyltransferase 1 [EC:2.1.2.2]
1.453	9.52E-04	Unclassified	Protein families: metabolism	Peptidases and inhibitors	K04773 - protease IV, sppA
1.446	4.56E-03	Cellular Processes	Cellular community-prokaryotes	Biofilm formation - Vibrio cholerae	K05946 - N-acetylglucosaminyldiphosphoundecaprenol N-acetyl-beta-D-mannosaminyltransferase [EC:2.4.1.187], tagA, tarA
1.434	1.39E-02	Metabolism	Metabolism of Cofactors and Vitamins	Folate biosynthesis	K03342 - para-aminobenzoate synthetase / 4-amino-4-deoxychorismate lyase [EC:2.6.1.85 4.1.3.38]
1.421	2.67E-03	Unclassified	Protein families: genetic information processing	Transcription factors	K07729 - putative transcriptional regulator
1.418	2.80E-02	Metabolism	Amino Acid Metabolism	Histidine metabolism	K00013 - histidinol dehydrogenase [EC:1.1.1.23]
1.416	1.21E-02	Unclassified	Unclassified: signaling and cellular processes	Others	K06201 - copper homeostasis protein, cutC
1.414	1.78E-02	Metabolism	Amino Acid Metabolism	Histidine metabolism	K00765 - ATP phosphoribosyltransferase [EC:2.4.2.17]
1.396	1.49E-02	Metabolism	Carbohydrate Metabolism	Pyruvate metabolism	K01649 - 2-isopropylmalate synthase [EC:2.3.3.13]
1.377	9.73E-04	Unclassified	Protein families: genetic information processing	Transcription factors	K03708 - transcriptional regulator of stress and heat shock response, ctsR
1.355	2.99E-02	Metabolism	Carbohydrate Metabolism	C5-Branched dibasic acid metabolism	K01703 - 3-isopropylmalate/(R)-2-methylmalate dehydratase large subunit [EC:4.2.1.33 4.2.1.35]
1.348	7.85E-03	Metabolism	Metabolism of Cofactors and Vitamins	Pantothenate and CoA biosynthesis	K00077 - 2-dehydropantoate 2-reductase [EC:1.1.1.169]
1.300	1.06E-03	Unclassified	Protein families: genetic information processing	Transcription factors	K10947 - PadR family transcriptional regulator, regulatory protein PadR
1.295	1.49E-02	Metabolism	Carbohydrate Metabolism	Glyoxylate and dicarboxylate metabolism	K00865 - glycerate kinase [EC:2.7.1.31]
1.287	3.30E-02	Metabolism	Carbohydrate Metabolism	Fructose and mannose metabolism	K02793 - PTS system, mannose-specific IIA component [EC:2.7.1.69]

1.286	2.86E-02	Environmental Information Processing	Membrane Transport	ABC transporters	K11050 - multidrug/hemolysin transport system ATP-binding protein
1.278	2.57E-03	Unclassified	Not included in Pathway or Brite	Poorly characterized	K09167 - uncharacterized protein
1.275	1.61E-02	Unclassified	Protein families: genetic information processing	Chromosome and associated proteins	K05896 - segregation and condensation protein A, scpA
1.205	2.54E-03	Environmental Information Processing	Membrane Transport	ABC transporters	K02018 - molybdate transport system permease protein
1.201	4.75E-02	Unclassified	Protein families: genetic information processing	Ribosome biogenesis	K03790 - [ribosomal protein S5]-alanine N-acetyltransferase [EC: 2.3.1.267], rimJ
1.198	8.38E-03	Unclassified	Unclassified: signaling and cellular processes	Structural proteins	K07282 - gamma-polyglutamate biosynthesis protein CapA
1.196	3.99E-02	Metabolism	Carbohydrate Metabolism	Pyruvate metabolism	K00027 - malate dehydrogenase (oxaloacetate-decarboxylating) [EC:1.1.1.38]
1.170	7.83E-03	Metabolism	Amino Acid Metabolism	Phenylalanine, tyrosine and tryptophan biosynthesis	K01736 - chorismate synthase [EC:4.2.3.5]
1.139	6.77E-03	Environmental Information Processing	Signal Transduction	Two-component system	K07646 - two-component system, OmpR family, sensor histidine kinase KdpD [EC:2.7.13.3]
1.115	4.76E-03	Unclassified	Not included in Pathway or Brite	Poorly characterized	K08981 - putative membrane protein
1.063	3.44E-02	Metabolism	Carbohydrate Metabolism	Glycolysis / Gluconeogenesis	K01803 - triosephosphate isomerase (TIM) [EC:5.3.1.1]
1.057	1.96E-03	Metabolism	Carbohydrate Metabolism	Glycolysis / Gluconeogenesis	K01785 - aldose 1-epimerase [EC:5.1.3.3]
1.006	3.92E-02	Cellular Processes	Transport and catabolism	Autophagy-yeast	K06902 - MFS transporter, UMF1 family
1.002	3.68E-02	Metabolism	Amino Acid Metabolism	Phenylalanine, tyrosine and tryptophan biosynthesis	K01735 - 3-dehydroquinate synthase [EC:4.2.3.4]
0.991	3.44E-02	Metabolism	Energy Metabolism	Nitrogen metabolism	K00605 - aminomethyltransferase [EC:2.1.2.10]
0.938	4.03E-02	Unclassified	Unclassified: signaling and cellular processes	Transport	K03294 - basic amino acid/polyamine antiporter, APA family
0.935	4.55E-03	Unclassified	Unclassified: metabolism	Enzymes with EC numbers	K05985 - ribonuclease M5 [EC: 3.1.26.8], rnmV
0.915	1.14E-02	Unclassified	Protein families: genetic information processing	Ribosome biogenesis	K09761 - 16S rRNA (uracil1498-N3)-methyltransferase [EC: 2.1.1.193], rsmE
0.913	6.82E-03	Unclassified	Unclassified: signaling and cellular processes	Transport	K03308 - neurotransmitter:Na+ symporter, NSS family

0.911	4.71E-02	Unclassified	Not included in Pathway or Brite	Poorly characterized	K07078 - uncharacterized protein
0.907	4.01E-03	Unclassified	Protein families: signaling and cellular processes	Transporters	K01990 - ABC-2 type transport system ATP-binding protein
0.871	1.44E-02	Metabolism	Carbohydrate Metabolism	Glycolysis / Gluconeogenesis	K01835 - phosphoglucomutase [EC:5.4.2.2]
0.840	2.17E-02	Metabolism	Metabolism of Terpenoids and Polyketides	Terpenoid backbone biosynthesis	K00099 - 1-deoxy-D-xylulose-5-phosphate reductoisomerase [EC:1.1.1.267]
0.816	2.29E-02	Environmental Information Processing	Membrane Transport	ABC transporters	K18104 - ATP-binding cassette, subfamily B, bacterial AbcA/BmrA [EC:7.6.2.2]
0.799	3.58E-02	Genetic Information Processing	Translation	RNA transport	K00784 - ribonuclease Z [EC:3.1.26.11]
0.791	2.77E-03	Metabolism	Amino Acid Metabolism	Arginine and proline metabolism	K00931 - glutamate 5-kinase [EC:2.7.2.11]
0.790	2.33E-03	Metabolism	Nucleotide Metabolism	Pyrimidine metabolism	K01493 - dCMP deaminase [EC:3.5.4.12]
0.777	1.14E-02	Unclassified	Unclassified: metabolism	Others	K15975 - glyoxalase family protein
0.768	3.30E-02	Unclassified	Not included in Pathway or Brite	Poorly characterized	K07139 - uncharacterized protein
0.751	1.51E-02	Unclassified	Protein families: signaling and cellular processes	Transporters	K07238 - zinc transporter, ZIP family
0.737	4.72E-02	Unclassified	Protein families: signaling and cellular processes	Two-component system	K07183 - two-component system, response regulator / RNA-binding antiterminator, nasT
0.717	3.91E-02	Metabolism	Glycan Biosynthesis and Metabolism	Peptidoglycan biosynthesis	K12555 - penicillin-binding protein 2A [EC:2.4.1.129 2.3.2.-]
0.716	4.75E-02	Metabolism	Metabolism of Cofactors and Vitamins	Porphyrin and chlorophyll metabolism	K00231 - oxygen-dependent protoporphyrinogen oxidase [EC:1.3.3.4]
0.708	4.05E-02	Metabolism	Amino Acid Metabolism	Valine, leucine and isoleucine degradation	K00020 - 3-hydroxyisobutyrate dehydrogenase [EC:1.1.1.31]
0.701	4.02E-02	Unclassified	Protein families: genetic information processing	Transcription factors	K07723 - CopG family transcriptional regulator / antitoxin EndoAI, ndoAI
0.670	1.34E-02	Metabolism	Nucleotide Metabolism	Purine metabolism	K00527 - ribonucleoside-triphosphate reductase [EC:1.17.4.2]
0.648	1.14E-02	Environmental Information Processing	Signal Transduction	Two-component system	K11618 - two-component system, NarL family, response regulator LiaR
0.597	4.82E-02	Unclassified	Protein families: signaling and cellular processes	Transporters	K06147 - ATP-binding cassette, subfamily B, bacterial

0.564	3.95E-02	Genetic Information Processing	Folding, Sorting and Degradation	Protein export	K03070 - preprotein translocase subunit SecA
0.550	1.46E-02	Metabolism	Nucleotide Metabolism	Purine metabolism	K01589 - 5-(carboxyamino)imidazole ribonucleotide synthase [EC:6.3.4.18]
0.543	2.17E-02	Unclassified	Protein families: genetic information processing	Transcription factors	K09685 - purine operon repressor, purR
0.521	4.95E-02	Metabolism	Energy Metabolism	Methane metabolism	K00831 - phosphoserine aminotransferase [EC:2.6.1.52]
0.501	4.59E-03	Metabolism	Carbohydrate Metabolism	Starch and sucrose metabolism	K07024 - sucrose-6-phosphatase [EC:3.1.3.24], SPP
0.447	1.05E-02	Metabolism	Lipid Metabolism	Glycerophospholipid metabolism	K06131 - cardiolipin synthase [EC:2.7.8.-]
-0.384	1.71E-02	Unclassified	Not included in Pathway or Brite	Poorly characterized	K07030 - uncharacterized protein
-0.396	2.73E-02	Metabolism	Metabolism of Terpenoids and Polyketides	Terpenoid backbone biosynthesis	K00805 - heptaprenyl diphosphate synthase [EC:2.5.1.30]
-0.414	4.80E-02	Metabolism	Amino Acid Metabolism	Glycine, serine and threonine metabolism	K00872 - homoserine kinase [EC:2.7.1.39]
-0.454	5.33E-03	Metabolism	Nucleotide Metabolism	Purine metabolism	K02428 - dITP/XTP pyrophosphatase [EC:3.6.1.19]
-0.519	4.44E-02	Metabolism	Metabolism of Terpenoids and Polyketides	Terpenoid backbone biosynthesis	K00938 - phosphomevalonate kinase [EC:2.7.4.2]
-0.560	2.46E-02	Genetic Information Processing	Replication and Repair	Mismatch repair	K03572 - DNA mismatch repair protein MutL
-0.570	1.00E-02	Unclassified	Not included in Pathway or Brite	Poorly characterized	K09963 - uncharacterized protein
-0.581	2.33E-02	Unclassified	Protein families: genetic information processing	Ribosome biogenesis	K03500 - 16S rRNA (cytosine967-C5)-methyltransferase [EC:2.1.1.176], rsmB
-0.583	2.80E-02	Unclassified	Protein families: genetic information processing	Transfer RNA biogenesis	K03439 - tRNA (guanine-N7-)-methyltransferase [EC:2.1.1.33], trmB
-0.592	1.44E-02	Metabolism	Nucleotide Metabolism	Purine metabolism	K01939 - adenylosuccinate synthase [EC:6.3.4.4]
-0.616	1.84E-02	Unclassified	Protein families: genetic information processing	Transfer RNA biogenesis	K07560 - D-aminoacyl-tRNA deacylase, dtd
-0.618	3.99E-02	Genetic Information Processing	Folding, Sorting and Degradation	Protein export	K12257 - SecD/SecF fusion protein, SecDF
-0.621	6.75E-03	Unclassified	Protein families: metabolism	Peptidases and inhibitors	K03797 - carboxyl-terminal processing protease [EC:3.4.21.102], ctpA, prc
-0.621	7.48E-03	Unclassified	Protein families: metabolism	Peptidases and inhibitors	K01419 - ATP-dependent HslUV protease, peptidase subunit HslV [EC:3.4.25.2]

-0.623	4.39E-02	Metabolism	Lipid Metabolism	Fatty acid biosynthesis	K00059 - 3-oxoacyl-[acyl-carrier protein] reductase [EC:1.1.1.100], fabG
-0.637	1.47E-02	Unclassified	Protein families: metabolism	Peptidases and inhibitors	K01262 - Xaa-Pro aminopeptidase [EC: 3.4.11.9], pepP
-0.651	1.66E-02	Metabolism	Metabolism of other Amino Acids	D-Glutamine and D-glutamate metabolism	K01925 - UDP-N-acetylmuramoylalanine--D-glutamate ligase, murD
-0.659	3.99E-02	Environmental Information Processing	Signal Transduction	Two-component system	K07775 - two-component system, OmpR family, response regulator ResD
-0.661	2.54E-02	Unclassified	Protein families: signaling and cellular processes	Transporters	K05569 - multicomponent Na+:H+ antiporter subunit E, mnhE, mrpE
-0.677	1.24E-02	Unclassified	Unclassified: metabolism	Enzymes with EC numbers	K00540 - F420H(2)-dependent quinone reductase [EC:1.1.98.-], fqr
-0.688	2.80E-02	Unclassified	Protein families: genetic information processing	Ribosome biogenesis	K14540 - ribosome biogenesis GTPase A, rbgA
-0.697	7.05E-03	Metabolism	Nucleotide Metabolism	Purine metabolism	K00951 - GTP pyrophosphokinase [EC:2.7.6.5]
-0.698	2.49E-02	Unclassified	Protein families: genetic information processing	Transcription machinery	K02601 - transcription termination/antitermination protein NusG
-0.706	2.52E-02	Unclassified	Protein families: metabolism	Peptidases and inhibitors	K01299 - carboxypeptidase Taq [EC:3.4.17.19]
-0.713	4.80E-02	Genetic Information Processing	Replication and Repair	Base excision repair	K01142 - exodeoxyribonuclease III [EC:3.1.11.2]
-0.716	1.06E-03	Metabolism	Metabolism of Cofactors and Vitamins	Thiamine metabolism	K04487 - cysteine desulfurase [EC:2.8.1.7]
-0.730	3.22E-03	Unclassified	Protein families: signaling and cellular processes	Antimicrobial resistance genes	K00662 - aminoglycoside 3-N-acetyltransferase [EC: 2.3.1.81]
-0.744	1.09E-02	Metabolism	Metabolism of Cofactors and Vitamins	Porphyrin and chlorophyll metabolism	K01749 - hydroxymethylbilane synthase [EC:2.5.1.61]
-0.744	1.14E-02	Metabolism	Energy Metabolism	Oxidative phosphorylation	K02826 - cytochrome aa3-600 menaquinol oxidase subunit II [EC:1.10.3.12]
-0.745	4.94E-02	Metabolism	Lipid Metabolism	Fatty acid biosynthesis	K10780 - enoyl-[acyl carrier protein] reductase III [EC:1.3.1.-]
-0.751	1.65E-03	Unclassified	Not included in Pathway or Brite	Poorly characterized	K07058 - membrane protein
-0.762	2.51E-02	Metabolism	Carbohydrate Metabolism	Butanoate metabolism	K01641 - hydroxymethylglutaryl-CoA synthase [EC:2.3.3.10]
-0.764	2.59E-02	Metabolism	Glycan Biosynthesis and Metabolism	Peptidoglycan biosynthesis	K07260 - D-alanyl-D-alanine carboxypeptidase [EC:3.4.16.4]
-0.778	1.31E-02	Metabolism	Nucleotide Metabolism	Purine metabolism	K02338 - DNA polymerase III subunit beta [EC:2.7.7.7]
-0.795	2.19E-02	Unclassified	Unclassified: metabolism	Others	K06999 - phospholipase/carboxylesterase

-0.804	4.91E-02	Environmental Information Processing	Membrane Transport	Phosphotransferase system (PTS)	K02784 - phosphocarrier protein HPr, ptsH
-0.805	1.41E-02	Metabolism	Amino Acid Metabolism	Lysine biosynthesis	K01714 - dihydrodipicolinate synthase [EC:4.2.1.52]
-0.809	4.75E-04	Metabolism	Metabolism of Terpenoids and Polyketides	Terpenoid backbone biosynthesis	K13789 - geranylgeranyl diphosphate synthase, type II [EC:2.5.1.1 2.5.1.10 2.5.1.29]
-0.825	3.63E-02	Unclassified	Not included in Pathway or Brite	Poorly characterized	K07040 - uncharacterized protein
-0.835	3.26E-02	Unclassified	Unclassified: metabolism	Amino acid metabolism	K06997 - PLP dependent protein, yggS
-0.837	1.00E-02	Metabolism	Energy Metabolism	Oxidative phosphorylation	K02827 - cytochrome aa3-600 menaquinol oxidase subunit I [EC:1.10.3.12]
-0.846	1.44E-02	Unclassified	Protein families: genetic information processing	Transfer RNA biogenesis	K00773 - queuine tRNA-ribosyltransferase [EC: 2.4.2.29], tgt
-0.846	1.25E-02	Genetic Information Processing	Replication and Repair	Nucleotide excision repair	K03702 - excinuclease ABC subunit B
-0.846	1.28E-03	Metabolism	Metabolism of Cofactors and Vitamins	Thiamine metabolism	K03151 - thiamine biosynthesis protein ThiI
-0.850	1.45E-02	Metabolism	Nucleotide Metabolism	Purine metabolism	K00940 - nucleoside-diphosphate kinase [EC:2.7.4.6]
-0.851	3.18E-03	Metabolism	Metabolism of Cofactors and Vitamins	Porphyrin and chlorophyll metabolism	K01719 - uroporphyrinogen-III synthase [EC:4.2.1.75]
-0.862	4.26E-03	Organismal Systems	Digestive System	Mineral absorption	K07213 - copper chaperone
-0.863	4.31E-03	Unclassified	Protein families: genetic information processing	Transfer RNA biogenesis	K07568 - S-adenosylmethionine:tRNA ribosyltransferase-isomerase [EC: 2.4.99.17], queA
-0.871	2.88E-02	Genetic Information Processing	Translation	Ribosome	K02970 - small subunit ribosomal protein S21
-0.884	5.45E-04	Genetic Information Processing	Replication and Repair	Homologous recombination	K03581 - exodeoxyribonuclease V alpha subunit [EC:3.1.11.5]
-0.886	3.13E-02	Unclassified	Unclassified: signaling and cellular processes	Cell growth	K06412 - stage V sporulation protein G, spoVG
-0.887	2.80E-02	Metabolism	Carbohydrate Metabolism	Glycolysis / Gluconeogenesis	K00873 - pyruvate kinase [EC:2.7.1.40]
-0.887	1.64E-03	Environmental Information Processing	Membrane Transport	ABC transporters	K15583 - oligopeptide transport system ATP-binding protein
-0.913	2.31E-02	Unclassified	Protein families: genetic information processing	Messenger RNA biogenesis	K03698 - 3'-5' exoribonuclease [EC:3.1.-.-], cbf
-0.918	3.15E-03	Genetic Information Processing	Translation	Aminoacyl-tRNA biosynthesis	K01893 - asparaginyl-tRNA synthetase [EC:6.1.1.22]

-0.919	3.28E-03	Environmental Information Processing	Membrane Transport	ABC transporters	K16013 - ATP-binding cassette, subfamily C, bacterial CydD
-0.927	3.25E-02	Environmental Information Processing	Membrane Transport	ABC transporters	K02002 - glycine betaine/proline transport system substrate-binding protein
-0.930	2.17E-02	Unclassified	Unclassified: metabolism	Enzymes with EC numbers	K09162 - chlorite dismutase [EC:1.13.11.49], cld
-0.931	5.46E-03	Unclassified	Unclassified: signaling and cellular processes	Cell growth	K06346 - spoIIIJ-associated protein
-0.932	7.96E-03	Metabolism	Energy Metabolism	Nitrogen metabolism	K01953 - asparagine synthase (glutamine-hydrolysing) [EC:6.3.5.4]
-0.938	3.67E-03	Metabolism	Metabolism of Cofactors and Vitamins	Vitamin B6 metabolism	K00868 - pyridoxine kinase [EC:2.7.1.35]
-0.943	5.58E-04	Metabolism	Lipid Metabolism	Fatty acid biosynthesis	K00208 - enoyl-[acyl-carrier protein] reductase I [EC:1.3.1.9]
-0.959	3.23E-02	Metabolism	Glycan Biosynthesis and Metabolism	Peptidoglycan biosynthesis	K05364 - penicillin-binding protein A, pbpA
-0.966	7.90E-03	Unclassified	Protein families: metabolism	Peptidases and inhibitors	K01271 - Xaa-Pro dipeptidase [EC:3.4.13.9], pepQ
-0.975	7.99E-03	Genetic Information Processing	Folding, Sorting and Degradation	Protein export	K03076 - preprotein translocase subunit SecY
-0.979	1.45E-02	Unclassified	Protein families: genetic information processing	Transfer RNA biogenesis	K11991 - tRNA (adenine34) deaminase [EC: 3.5.4.33], tadA
-0.982	2.00E-02	Metabolism	Glycan Biosynthesis and Metabolism	Peptidoglycan biosynthesis	K05366 - penicillin-binding protein 1A [EC:2.4.1.- 3.4.-.-]
-0.985	1.23E-05	Genetic Information Processing	Replication and Repair	Nucleotide excision repair	K03703 - excinuclease ABC subunit C, uvrC
-0.991	5.91E-03	Metabolism	Nucleotide Metabolism	Pyrimidine metabolism	K00761 - uracil phosphoribosyltransferase [EC:2.4.2.9]
-0.993	7.59E-04	Unclassified	Unclassified: genetic information processing	Protein processing	K03664 - SsrA-binding protein
-0.997	9.73E-03	Genetic Information Processing	Folding, Sorting and Degradation	Protein export	K03101 - signal peptidase II [EC:3.4.23.36]
-1.017	3.40E-03	Unclassified	Protein families: genetic information processing	DNA repair and recombinationn proteins	K03502 - DNA polymerase V, umuC
-1.019	1.49E-02	Genetic Information Processing	Translation	Ribosome	K02986 - small subunit ribosomal protein S4
-1.026	3.80E-03	Genetic Information Processing	Translation	Aminoacyl-tRNA biosynthesis	K09698 - nondiscriminating glutamyl-tRNA synthetase [EC:6.1.1.24]
-1.030	3.21E-04	Metabolism	Metabolism of Cofactors and Vitamins	Porphyrin and chlorophyll metabolism	K01845 - glutamate-1-semialdehyde 2,1-aminomutase, hemL [EC:5.4.3.8]

-1.032	2.09E-09	Unclassified	Unclassified: signaling and cellular processes	Transport	K03306 - inorganic phosphate transporter, PiT family
-1.037	5.22E-03	Genetic Information Processing	Replication and Repair	DNA replication	K03111 - single-strand DNA-binding protein
-1.041	5.90E-03	Environmental Information Processing	Membrane Transport	ABC transporters	K16012 - ATP-binding cassette, subfamily C, bacterial CydC
-1.041	5.58E-04	Metabolism	Metabolism of Cofactors and Vitamins	Folate biosynthesis	K00287 - dihydrofolate reductase [EC:1.5.1.3]
-1.046	1.53E-03	Unclassified	Protein families: genetic information processing	Chaperones and folding catalysts	K04083 - molecular chaperone Hsp33, hslO
-1.049	1.01E-02	Metabolism	Carbohydrate Metabolism	Glycolysis / Gluconeogenesis	K15633 - 2,3-bisphosphoglycerate-independent phosphoglycerate mutase [EC:5.4.2.1]
-1.059	1.01E-02	Genetic Information Processing	Translation	Ribosome	K02990 - small subunit ribosomal protein S6
-1.068	1.30E-02	Genetic Information Processing	Translation	Aminoacyl-tRNA biosynthesis	K01881 - prolyl-tRNA synthetase [EC:6.1.1.15]
-1.074	2.68E-03	Genetic Information Processing	Replication and Repair	Nucleotide excision repair	K03701 - excinuclease ABC subunit A
-1.090	3.87E-04	Metabolism	Nucleotide Metabolism	Purine metabolism	K00962 - polyribonucleotide nucleotidyltransferase [EC:2.7.7.8]
-1.096	4.57E-04	Unclassified	Protein families: genetic information processing	DNA replication proteins	K02621 - topoisomerase IV subunit A [EC: 5.6.2.2], parC
-1.107	6.92E-05	Unclassified	Protein families: genetic information processing	Translation factors	K02838 - ribosome recycling factor
-1.111	8.55E-03	Metabolism	Nucleotide Metabolism	Pyrimidine metabolism	K00384 - thioredoxin reductase (NADPH) [EC:1.8.1.9]
-1.114	5.45E-04	Genetic Information Processing	Folding, Sorting and Degradation	Sulfur relay system	K00566 - tRNA-specific 2-thiouridylase [EC:2.8.1.-]
-1.114	1.23E-05	Metabolism	Metabolism of Cofactors and Vitamins	Porphyrin and chlorophyll metabolism	K01599 - uroporphyrinogen decarboxylase hemE [EC:4.1.1.37]
-1.116	3.67E-03	Metabolism	Carbohydrate Metabolism	Pentose phosphate pathway	K00033 - 6-phosphogluconate dehydrogenase [EC:1.1.1.44]
-1.119	1.51E-02	Metabolism	Carbohydrate Metabolism	Glycolysis / Gluconeogenesis	K02777 - PTS system, glucose-specific IIA component [EC:2.7.1.69]
-1.134	1.96E-02	Metabolism	Carbohydrate Metabolism	Pyruvate metabolism	K00656 - formate C-acetyltransferase [EC:2.3.1.54]
-1.145	1.71E-04	Metabolism	Metabolism of Other Amino Acids	Glutathione metabolism	K01919 - glutamate--cysteine ligase gshA [EC:6.3.2.2]
-1.147	7.81E-03	Genetic Information Processing	Folding, Sorting and Degradation	RNA degradation	K12574 - ribonuclease J [EC:3.1.-.-]
-1.152	3.41E-02	Metabolism	Carbohydrate Metabolism	Pentose phosphate pathway	K01839 - phosphopentomutase [EC:5.4.2.7]

-1.154	2.17E-02	Metabolism	Lipid Metabolism	Arachidonic acid metabolism	K00432 - glutathione peroxidase [EC:1.11.1.9]
-1.154	3.73E-02	Unclassified	Protein families: metabolism	Peptidases and inhibitors	K01372 - bleomycin hydrolase [EC: 3.4.22.40], pepC
-1.154	3.91E-03	Unclassified	Protein families: genetic information processing	Transcription factors	K17472 - Rrf2 family transcriptional regulator, cysteine metabolism repressor, cymR
-1.156	1.28E-03	Metabolism	Carbohydrate Metabolism	Glycolysis / Gluconeogenesis	K01624 - fructose-bisphosphate aldolase, class II [EC:4.1.2.13]
-1.157	5.45E-04	Metabolism	Metabolism of Cofactors and Vitamins	Pantothenate and CoA biosynthesis	K03525 - type III pantothenate kinase [EC:2.7.1.33]
-1.175	6.77E-03	Unclassified	Protein families: genetic information processing	DNA replication proteins	K02469 - DNA gyrase subunit A [EC:5.6.2.2]
-1.178	7.96E-04	Unclassified	Not included in Pathway or Brite	Poorly characterized	K09976 - uncharacterized protein
-1.179	2.33E-02	Metabolism	Carbohydrate Metabolism	Butanoate metabolism	K01580 - glutamate decarboxylase [EC:4.1.1.15]
-1.192	6.27E-04	Unclassified	Not included in Pathway or Brite	Poorly characterized	K09764 - uncharacterized protein
-1.200	5.09E-03	Genetic Information Processing	Translation	Ribosome	K02907 - large subunit ribosomal protein L30
-1.207	2.82E-03	Metabolism	Metabolism of Cofactors and Vitamins	Thiamine metabolism	K06949 - ribosome biogenesis GTPase / thiamine phosphate phosphatase [EC: 3.6.1.- 3.1.3.100], rsgA, engC
-1.211	1.51E-02	Unclassified	Unclassified: metabolism	Enzymes with EC numbers	K07447 - putative holliday junction resolvase [EC:3.1.-.-], ruvX
-1.228	1.21E-02	Metabolism	Carbohydrate Metabolism	Glycolysis / Gluconeogenesis	K00927 - phosphoglycerate kinase [EC:2.7.2.3]
-1.237	1.51E-02	Unclassified	Protein families: genetic information processing	Transcription machinery	K05518 - phosphoserine phosphatase RsbX [EC: 3.1.3.3], rsbX
-1.241	3.92E-04	Genetic Information Processing	Replication and Repair	Homologous recombination	K03655 - ATP-dependent DNA helicase RecG [EC:3.6.4.12]
-1.245	2.81E-03	Unclassified	Protein families: genetic information processing	Translation factors	K02357 - elongation factor Ts
-1.246	2.33E-03	Genetic Information Processing	Translation	Ribosome	K02871 - large subunit ribosomal protein L13
-1.249	1.71E-02	Metabolism	Carbohydrate Metabolism	Fructose and mannose metabolism	K02795 - PTS system, mannose-specific IIC component
-1.250	3.57E-03	Metabolism	Energy Metabolism	Sulfur metabolism	K01738 - cysteine synthase A [EC:2.5.1.47]
-1.250	5.00E-04	Metabolism	Energy Metabolism	Oxidative phosphorylation	K02113 - F-type H+-transporting ATPase subunit delta [EC:3.6.3.14], atpH
-1.259	4.05E-03	Metabolism	Carbohydrate Metabolism	Glycolysis / Gluconeogenesis	K01689 - enolase [EC:4.2.1.11]

-1.269	5.11E-03	Genetic Information Processing	Translation	Ribosome	K02887 - large subunit ribosomal protein L20
-1.274	1.08E-03	Environmental Information Processing	Membrane Transport	ABC transporters	K10823 - oligopeptide transport system ATP-binding protein
-1.280	5.00E-04	Genetic Information Processing	Translation	Ribosome	K02988 - small subunit ribosomal protein S5
-1.308	7.30E-04	Metabolism	Metabolism of Other Amino Acids	Selenocompound metabolism	K11717 - cysteine desulfurase / selenocysteine lyase [EC:2.8.1.7 4.4.1.16]
-1.313	8.20E-03	Unclassified	Unclassified: metabolism	Enzymes with EC numbers	K07305 - peptide-methionine (R)-S-oxide reductase [EC:1.8.4.12], msrB
-1.315	8.38E-03	Unclassified	Protein families: genetic information processing	Transcription factors	K01926 - redox-sensing transcriptional repressor, rex
-1.317	7.67E-04	Unclassified	Protein families: metabolism	Peptidoglycan biosynthesis and degradation proteins	K06078 - murein lipoprotein, lpp
-1.319	9.73E-04	Unclassified	Protein families: genetic information processing	Chaperones and folding catalysts	K03768 - peptidyl-prolyl cis-trans isomerase B (cyclophilin B) [EC: 5.2.1.8] PPIB, ppiB
-1.323	3.54E-03	Metabolism	Amino Acid Metabolism	Valine, leucine and isoleucine degradation	K09699 - 2-oxoisovalerate dehydrogenase E2 component (dihydrolipoyl transacylase) [EC:2.3.1.168]
-1.324	1.48E-03	Unclassified	Protein families: genetic information processing	Transfer RNA biogenesis	K03216 - tRNA (cytidine/uridine-2'-O-)-methyltransferase [EC: 2.1.1. 207]
-1.328	6.05E-04	Environmental Information Processing	Membrane Transport	ABC transporters	K18891 - ATP-binding cassette, subfamily B, multidrug efflux pump, patA, rscA, lmrC, satA
-1.337	9.73E-04	Genetic Information Processing	Translation	Ribosome	K02874 - large subunit ribosomal protein L14
-1.338	8.32E-03	Unclassified	Protein families: genetic information processing	Ribosome biogenesis	K02834 - ribosome-binding factor A, rbfA
-1.340	1.24E-03	Genetic Information Processing	Translation	Ribosome	K02926 - large subunit ribosomal protein L4
-1.342	1.53E-03	Unclassified	Protein families: signaling and cellular processes	Transporters	K03282 - large conductance mechanosensitive channel, mscL
-1.343	1.36E-03	Environmental Information Processing	Membrane Transport	ABC transporters	K18892 - ATP-binding cassette, subfamily B, multidrug efflux pump, patB, rscB, lmrC, satB
-1.344	7.59E-04	Unclassified	Protein families: genetic information processing	Translation factors	K02520 - translation initiation factor IF-3, MTIF3, infC

-1.345	1.43E-02	Unclassified	Not included in Pathway or Brite	Poorly characterized	K09117 - uncharacterized protein
-1.351	4.35E-04	Genetic Information Processing	Translation	Ribosome	K02994 - small subunit ribosomal protein S8, rpsH
-1.352	1.47E-03	Genetic Information Processing	Translation	Ribosome	K02946 - small subunit ribosomal protein S10
-1.359	5.35E-04	Metabolism	Energy Metabolism	Oxidative phosphorylation	K02829 - cytochrome aa3-600 menaquinol oxidase subunit IV [EC:1.10.3.12]
-1.360	1.23E-05	Unclassified	Protein families: signaling and cellular processes	Transporters	K06901 - adenine/guanine/hypoxanthine permease, pbuG, azgA, ghxP, ghxQ, adeQ
-1.364	3.92E-04	Metabolism	Nucleotide Metabolism	Purine metabolism	K00759 - adenine phosphoribosyltransferase [EC:2.4.2.7]
-1.365	7.78E-04	Metabolism	Amino Acid Metabolism	Valine, leucine and isoleucine degradation	K00167 - 2-oxoisovalerate dehydrogenase E1 component, beta subunit [EC:1.2.4.4]
-1.379	3.18E-03	Metabolism	Carbohydrate Metabolism	Pentose phosphate pathway	K00036 - glucose-6-phosphate 1-dehydrogenase [EC:1.1.1.49]
-1.379	4.01E-03	Unclassified	Unclassified: metabolism	Enzymes with EC numbers	K11065 - thioredoxin-dependent peroxiredoxin [EC: 1.11.1.24], tpx
-1.382	6.91E-03	Unclassified	Protein families: signaling and cellular processes	Transporters	K03284 - magnesium transporter, corA
-1.390	7.26E-03	Genetic Information Processing	Translation	Ribosome	K02931 - large subunit ribosomal protein L5
-1.391	1.01E-02	Environmental Information Processing	Membrane Transport	Phosphotransferase system (PTS)	K08483 - phosphotransferase system, enzyme I, PtsI [EC:2.7.3.9]
-1.396	1.27E-03	Genetic Information Processing	Translation	Ribosome	K02902 - large subunit ribosomal protein L28
-1.397	4.63E-03	Unclassified	Not included in Pathway or Brite	Poorly characterized	K07071 - uncharacterized protein
-1.400	6.54E-04	Metabolism	Amino Acid Metabolism	Valine, leucine and isoleucine degradation	K00166 - 2-oxoisovalerate dehydrogenase E1 component, alpha subunit [EC:1.2.4.4]
-1.411	2.86E-02	Unclassified	Protein families: genetic information processing	Transcription factors	K03706 - transcriptional pleiotropic repressor, CodY
-1.420	3.44E-02	Environmental Information Processing	Signal Transduction	Two-component system	K07706 - two-component system, LytTR family, sensor histidine kinase AgrC [EC:2.7.13.3], agrC
-1.421	2.16E-03	Unclassified	Unclassified: genetic information processing	Protein processing	K03545 - trigger factor, tig
-1.424	6.02E-04	Metabolism	Energy Metabolism	Oxidative phosphorylation	K02828 - cytochrome aa3-600 menaquinol oxidase subunit III [EC:1.10.3.12]
-1.444	5.58E-04	Unclassified	Protein families: metabolism	Peptidases and inhibitors	K03798 - cell division protease FtsH [EC: 3.4.24.-]

-1.463	5.00E-04	Genetic Information Processing	Translation	Ribosome	K02933 - large subunit ribosomal protein L6
-1.497	6.53E-03	Metabolism	Energy Metabolism	Oxidative phosphorylation	K02108 - F-type H+-transporting ATPase subunit a [EC:3.6.3.14]
-1.515	4.75E-04	Metabolism	Carbohydrate Metabolism	Glycolysis / Gluconeogenesis	K00161 - pyruvate dehydrogenase E1 component subunit alpha [EC:1.2.4.1], pdhA
-1.520	4.95E-05	Metabolism	Energy Metabolism	Oxidative phosphorylation	K03885 - NADH dehydrogenase ndh [EC:1.6.99.3]
-1.528	1.59E-03	Genetic Information Processing	Translation	Ribosome	K02996 - small subunit ribosomal protein S9
-1.528	5.14E-05	Metabolism	Metabolism of Cofactors and Vitamins	Porphyrin and chlorophyll metabolism	K01772 - protoporphyrin/coproporphyrin ferrochelatase hemH [EC:4.99.1.1]
-1.529	7.59E-04	Metabolism	Carbohydrate Metabolism	Amino sugar and nucleotide sugar metabolism	K00820 - glucosamine--fructose-6-phosphate aminotransferase (isomerizing) [EC:2.6.1.16]
-1.532	7.59E-04	Metabolism	Nucleotide Metabolism	Purine metabolism	K03040 - DNA-directed RNA polymerase subunit alpha [EC:2.7.7.6]
-1.534	1.10E-03	Organismal Systems	Environmental Adaptation	Plant-pathogen interaction	K02358 - elongation factor Tu
-1.535	3.61E-04	Genetic Information Processing	Translation	Ribosome	K02965 - small subunit ribosomal protein S19, rpsS
-1.536	1.76E-04	Genetic Information Processing	Translation	Ribosome	K02886 - large subunit ribosomal protein L2, rplB
-1.551	1.23E-05	Metabolism	Metabolism of Cofactors and Vitamins	Riboflavin metabolism	K11753 - riboflavin kinase / FMN adenylyltransferase ribF [EC:2.7.1.26 2.7.7.2]
-1.594	9.85E-03	Cellular Processes	Cell Growth and Death	Cell cycle - Caulobacter	K03544 - ATP-dependent Clp protease ATP-binding subunit ClpX
-1.607	3.59E-04	Unclassified	Not included in Pathway or Brite	Poorly characterized	K09014 - Fe-S cluster assembly protein SufB
-1.635	4.55E-03	Metabolism	Carbohydrate Metabolism	Glycolysis / Gluconeogenesis	K00134 - glyceraldehyde 3-phosphate dehydrogenase [EC:1.2.1.12]
-1.650	5.58E-04	Metabolism	Energy Metabolism	Oxidative phosphorylation	K02112 - F-type H+-transporting ATPase subunit beta [EC:3.6.3.14]
-1.731	2.10E-02	Genetic Information Processing	Folding, Sorting and Degradation	Protein export	K03210 - preprotein translocase subunit YajC
-1.732	2.67E-03	Cellular Processes	Transport and Catabolism	Peroxisome	K04564 - superoxide dismutase, Fe-Mn family [EC:1.15.1.1]
-1.736	4.57E-04	Metabolism	Carbohydrate Metabolism	Glycolysis / Gluconeogenesis	K00627 - pyruvate dehydrogenase E2 component (dihydrulipoamide acetyltransferase) [EC: 2.3.1.12], PdhC
-1.739	1.76E-05	Genetic Information Processing	Translation	Ribosome	K02982 - small subunit ribosomal protein S3, rpsC
-1.763	6.98E-03	Metabolism	Lipid Metabolism	Fatty acid biosynthesis	K02078 - acyl carrier protein, acpP
-1.808	1.36E-03	Metabolism	Carbohydrate Metabolism	Glycolysis / Gluconeogenesis	K00382 - dihydrolipoamide dehydrogenase [EC:1.8.1.4]
-1.821	9.28E-04	Unclassified	Unclassified: genetic information processing	Transcription	K03704 - cold shock protein, cspA
-1.861	3.03E-04	Genetic Information Processing	Translation	Ribosome	K02909 - large subunit ribosomal protein L31 rmpE
-1.880	5.08E-04	Metabolism	Energy Metabolism	Oxidative phosphorylation	K02115 - F-type H+-transporting ATPase subunit gamma [EC:3.6.3.14]

-1.897	1.27E-03	Metabolism	Carbohydrate Metabolism	Glycolysis / Gluconeogenesis	K00162 - pyruvate dehydrogenase E1 component beta subunit [EC: 1.2.4.1], pdhB
-2.101	5.45E-04	Genetic Information Processing	Translation	Ribosome	K02914 - large subunit ribosomal protein L34
-2.106	3.22E-03	Genetic Information Processing	Translation	Ribosome	K02935 - large subunit ribosomal protein L7/L12
-2.156	2.47E-07	Unclassified	Protein families: genetic information processing	Chromosome and associated proteins	K03569 - rod shape-determining protein MreB and related proteins
-2.222	2.31E-03	Metabolism	Carbohydrate Metabolism	Glycolysis / Gluconeogenesis	K04072 - acetaldehyde dehydrogenase / alcohol dehydrogenase [EC:1.2.1.10 1.1.1.1]
