# Supplementary material for: Unravelling the Molecular Mechanisms Underlying the Protective Effect of Lactate on the High-Pressure Resistance of Listeria monocytogenes
Source: Biomolecules. 2021 Apr 30;11(5):677. doi: 10.3390/biom11050677 (PMC8147161; doi:10.3390/biom11050677)
Supplement: Supplementary file 1 [file biomolecules-11-00677-s001.zip › biomolecules-1111984-proof-suppl/supplementary table 7.pdf]

Table S7. List of KEGG Orthology (KO) genes differentially (FDR<0.05) expressed in the *L. monocytogenes* strain EGDe in samples without lactate pressurized and non-pressurized. Positive Log2 fold change indicate genes more abundant in pressurized samples.

Log2 Fold Change	FDR	KEGG annotations at level 1	KEGG annotations at level 2	KEGG pathway	KEGG Orthology (KO) genes
2.475	4.57E-05	Environmental Information Processing	Signal Transduction	Two-component system	K04751 - nitrogen regulatory protein P-II 1; glnB
2.188	4.80E-06	Metabolism	Carbohydrate Metabolism	Glyoxylate and dicarboxylate metabolism	K01915 - glutamine synthetase [EC:6.3.1.2]
2.045	7.50E-03	Unclassified	Protein families: signaling and cellular processes	Transporters	K03320 - ammonium transporter, Amt family
1.880	3.81E-05	Metabolism	Lipid Metabolism	Glycerophospholipid metabolism	K00981 - phosphatidate cytidylyltransferase CDS1, CDS2, cdsA [EC:2.7.7.41]
1.839	3.10E-05	Metabolism	Metabolism of Terpenoids and Polyketides	Terpenoid backbone biosynthesis	K00099 - 1-deoxy-D-xylulose-5-phosphate reductoisomerase dxr [EC:1.1.1.267]
1.814	1.05E-02	Metabolism	Amino Acid Metabolism	Valine, leucine and isoleucine biosynthesis	K01687 - dihydroxy-acid dehydratase ilvD [EC:4.2.1.9]
1.570	5.20E-06	Metabolism	Carbohydrate Metabolism	Butanoate metabolism	K00634 - phosphate butyryltransferase ptb [EC:2.3.1.19]
1.464	4.44E-03	Metabolism	Metabolism of Other Amino Acids	D-Alanine metabolism	K14188 - D-alanine--poly (phosphoribitol) ligase subunit 2 DltC
1.400	1.53E-03	Genetic Information Processing	Translation	Aminoacyl-tRNA biosynthesis	K01881 - prolyl-tRNA synthetase proS, PARS [EC:6.1.1.15]
1.335	9.63E-04	Cellular Processes	Cell Growth and Death	Cell cycle - Caulobacter	K11749 - regulator of sigma E protease rseP [EC:3.4.24.-]
1.278	9.23E-05	Environmental Information Processing	Signal Transduction	Two-component system	K03740 - D-alanine transfer protein dltD
1.240	1.17E-03	Metabolism	Lipid Metabolism	Fatty acid biosynthesis	K00648 - 3-oxoacyl-[acyl-carrier-protein] synthase III fabH [EC:2.3.1.180]
1.240	1.10E-03	Metabolism	Lipid Metabolism	Fatty acid biosynthesis	K00208 - enoyl-[acyl-carrier protein] reductase I [EC:1.3.1.9 1.3.1.10] FabI
1.227	1.40E-06	Metabolism	Amino Acid Metabolism	Glycine, serine and threonine metabolism	K00003 - homoserine dehydrogenase hom [EC:1.1.1.3]
1.184	6.36E-06	Metabolism	Amino Acid Metabolism	Glycine, serine and threonine metabolism	K01733 - threonine synthase thrC [EC:4.2.3.1]
1.160	9.09E-03	Genetic Information Processing	Folding, Sorting and Degradation	RNA degradation	K12574 - ribonuclease J [EC:3.1.-.-]
1.153	1.57E-09	Metabolism	Amino Acid Metabolism	Glycine, serine and threonine metabolism	K00872 - homoserine kinase thrB1 [EC:2.7.1.39]

1.128	2.57E-02	Unclassified	Protein families: genetic information processing	Chromosome and associated proteins	K03570 - rod shape-determining protein MreC
1.113	4.94E-03	Genetic Information Processing	Replication and Repair	Base excision repair	K01151 - deoxyribonuclease IV nfo [EC:3.1.21.2]
1.107	3.26E-04	Unclassified	Signaling and cellular processes	Transport	K03316 - monovalent cation:H^+ antiporter, CPA1 family, TC.CPA1
1.097	9.24E-04	Metabolism	Nucleotide Metabolism	Purine metabolism	K03763 - DNA polymerase III subunit alpha polC, Gram-positive type [EC:2.7.7.7]
1.094	1.66E-03	Unclassified	Unclassified: metabolism	Enzymes with EC number	K04068 - anaerobic ribonucleoside-triphosphate reductase activating protein, nrdG
1.072	8.18E-03	Metabolism	Metabolism of Other Amino Acids	D-Alanine metabolism	K03367 - D-alanine--poly(phosphoribitol) ligase subunit 1 dltA [EC:6.1.1.13]
1.066	2.31E-04	Metabolism	Glycan biosynthesis and metabolism	Peptidoglycan biosynthesis	K18149 - penicillin-binding protein pbp5, pbp4, pbp3
1.055	5.36E-04	Metabolism	Nucleotide Metabolism	Purine metabolism	K00527 - ribonucleoside-triphosphate reductase (thioredoxin) rtpR [EC:1.17.4.2]
1.015	2.09E-02	Genetic Information Processing	Folding, Sorting and Degradation	Protein export	K03076 - preprotein translocase subunit SecY
0.965	1.28E-02	Environmental Information Processing	Signal Transduction	Two-component system	K11622 - lia operon protein LiaF
0.938	2.47E-02	Metabolism	Amino Acid Metabolism	Valine, leucine and isoleucine degradation	K00166 - 2-oxoisovalerate dehydrogenase E1 component, alpha subunit BCKDHA, bkdA1 [EC:1.2.4.4]
0.937	1.91E-03	Genetic Information Processing	Folding, Sorting and Degradation	RNA degradation	K12573 - ribonuclease R rnr, vacB [EC:3.1.-.-]
0.923	2.06E-02	Genetic Information Processing	Translation	Ribosome	K02988 - small subunit ribosomal protein S5, RP-S5, MRPS5
0.914	1.09E-03	Unclassified	Unclassified: genetic information processing	Protein processing	K03664 - SsrA-binding protein, smpB
0.892	1.53E-03	Unclassified	Protein families: genetic information processing	DNA replication proteins	K02622 - topoisomerase IV subunit B, parE
0.855	1.32E-02	Environmental Information Processing	Membrane Transport	ABC transporters	K18891 - ATP-binding cassette, subfamily B, multidrug efflux pump patA, rscA, lmrC, satA
0.854	1.98E-02	Metabolism	Amino Acid Metabolism	Glycine, serine and threonine metabolism	K00133 - aspartate-semialdehyde dehydrogenase asd [EC:1.2.1.11]
0.850	1.92E-03	Metabolism	Amino Acid Metabolism	Arginine and proline metabolism	K00931 - glutamate 5-kinase proB [EC:2.7.2.11]

0.847	1.37E-02	Unclassified	Protein families: metabolism	Peptidoglycan biosynthesis and degradation proteins	K06078 - murein lipoprotein lpp
0.831	4.80E-02	Genetic Information Processing	Translation	Ribosome	K02895 - large subunit ribosomal protein L24, RP-L24, MRPL24, rplX
0.825	9.15E-03	Metabolism	Carbohydrate Metabolism	Glycolisis/Gluconeogenesis	K00382 - dihydrolipoamide dehydrogenase DLD, lpd, pdhD
0.824	4.56E-02	Human Diseases	Infectious Diseases	Legionellosis	K03596 - GTP-binding protein LepA
0.810	4.63E-03	Unclassified	Protein families: genetic information processing	DNA replication proteins	K02621 - topoisomerase IV subunit A parC
0.809	3.71E-02	Unclassified	Protein families: genetic information processing	Transfer RNA biogenesis	K03439 - tRNA (guanine-N7-)-methyltransferase trmB, METTL1, TRM8
0.807	2.23E-02	Unclassified	Protein families: genetic information processing	DNA repair and recombination proteins	K02347 - DNA polymerase (family X) polX, dpx
0.801	1.20E-02	Unclassified	Protein families: genetic information processing	DNA replication proteins	K02469 - DNA gyrase subunit A gyrA
0.797	2.06E-02	Metabolism	Amino Acid Metabolism	Valine, leucine and isoleucine degradation	K00167 - 2-oxoisovalerate dehydrogenase E1 component, beta subunit BCKDHB, bkdA2 [EC:1.2.4.4]
0.793	1.92E-02	Unclassified	Unclassified: metabolism	Enzymes with EC number	K03928 - carboxylesterase yvaK
0.788	1.14E-02	Genetic Information Processing	Replication and Repair	Mismatch repair	K03572 - DNA mismatch repair protein MutL
0.769	2.59E-03	Metabolism	Lipid Metabolism	Glycerolipid metabolism	K03621 - phosphate acyltransferase PlsX
0.768	1.28E-02	Unclassified	Protein families: genetic information processing	Translation factors	K02519 -translation initiation factor IF-2, infB, MTIF2
0.763	2.01E-02	Metabolism	Metabolism of Terpenoids and Polyketides	Terpenoid backbone biosynthesis	K01662 - 1-deoxy-D-xylulose-5-phosphate synthase dxs [EC:2.2.1.7]
0.761	3.82E-02	Unclassified	Protein families: signaling and cellular processes	Transporters	K18926 - MFS transporter, DHA2 family, lincomycin resistance protein lmrB
0.752	3.89E-02	Metabolism	Amino Acid Metabolism	Lysine biosynthesis	K01714 - dihydrodipicolinate synthase dapA [EC:4.2.1.52]
0.745	4.00E-02	Genetic Information Processing	Replication and Repair	Nucleotide excision repair	K03701 - excinuclease ABC subunit A uvrA
0.742	1.21E-02	Metabolism	Metabolism of Cofactors and Vitamins	Vitamin B6 metabolism	K08681 - 5'-phosphate synthase pdxT subunit pdx2, pdxT

0.729	3.93E-02	Genetic Information Processing	Translation	Aminoacyl-tRNA biosynthesis	K01876 - aspartyl-tRNA synthetase aspS [EC:6.1.1.12]
0.717	1.20E-02	Metabolism	Amino Acid Metabolism	Lysine biosynthesis	K01929 - UDP-N-acetylmuramoyl-tripeptide--D-alanyl-D-alanine ligase murF [EC:6.3.2.10]
0.715	4.91E-02	Unclassified	Protein families: genetic information processing	Transcription machinery	K02600 - transcription termination/antitermination protein NusA
0.714	3.56E-02	Unclassified	Protein families: genetic information processing	Translation factors	K02837 - peptide chan release factor 3 prfC
0.696	2.82E-02	Metabolism	Lipid Metabolism	Fatty acid biosynthesis	K00645 - [acyl-carrier-protein] S-malonyltransferase FabD
0.690	2.82E-02	Genetic Information Processing	Folding, Sorting and Degradation	RNA degradation	K04043 - molecular chaperone DnaK
0.686	4.81E-02	Metabolism	Nucleotide Metabolism	Purine metabolism	K02337 - DNA polymerase III subunit alpha DnaE [EC:2.7.7.7]
0.685	4.43E-02	Genetic Information Processing	Translation	Aminoacyl-tRNA biosynthesis	K01892 - histidyl-tRNA synthetase hisS [EC:6.1.1.21]
0.684	2.82E-02	Environmental Information Processing	Signal Transduction	Two-component system	K11618 - two-component system, NarL family, response regulator LiaR
0.683	4.81E-02	Genetic Information Processing	Translation	Aminoacyl-tRNA biosynthesis	K01868 - threonyl-tRNA synthetase thrS [EC:6.1.1.3]
0.682	1.14E-02	Unclassified	Protein families: signaling and cellular processes	Transporters	K03442 - small conductance mechanosensitive channel mscS
0.679	1.75E-02	Metabolism	Amino Acid Metabolism	Glycine, serine and threonine metabolism	K00928 - aspartate kinase lysC [EC:2.7.2.4]
0.673	1.14E-02	Genetic Information Processing	Translation	Aminoacyl-tRNA biosynthesis	K04567 - lysyl-tRNA synthetase, class II lysS [EC:6.1.1.6]
0.672	1.21E-02	Human Diseases	Infectious Diseases	Staphylococcus aureus infection	K14205 - phosphatidylglycerol lysyltransferase mprF, fmtC [EC:2.3.2.3]
0.667	4.73E-02	Unclassified	Protein families: genetic information processing	DNA repair and recombination proteins	K03502 - DNA polymerase V umuC
0.660	3.36E-03	Unclassified	Unclassified: genetic information processing	Transcription	K06959 - protein Tex
0.649	1.61E-02	Metabolism	Metabolism of Other Amino Acids	D-Glutamine and D-glutamate metabolism	K01925 - UDP-N-acetylmuramoylalanine--D-glutamate ligase, MurD

0.645	4.81E-02	Unclassified	Protein families: genetic information processing	Transfer RNA biogenesis	K00773 - queuine tRNA-ribosyltransferas, tgt, QTRT1
0.638	3.71E-02	Environmental Information Processing	Signal Transduction	Two-component system	K11617 - two-component system, NarL family, sensor histidine kinase LiaS [EC:2.7.13.3]
0.630	1.15E-02	Unclassified	Protein families: genetic information processing	Ribosome biogenesis	K06442 - 23S rRNA (cytidine1920-2'-O)/16S rRNA (cytidine1409-2'-O)-methyltransferase tlyA
0.592	4.73E-02	Metabolism	Metabolism of Other Amino Acids	Selenocompound metabolism	K01874 - methionyl-tRNA synthetase metG [EC:6.1.1.10]
0.582	2.90E-02	Unclassified	Protein families: genetic information processing	Chromosome and associated proteins	K03569 - rod shape-determining protein MreB and related proteins
0.582	1.09E-03	Metabolism	Amino Acid Metabolism	Phenylalanine, tyrosine and tryptophan biosynthesis	K00014 - shikimate dehydrogenase [EC:1.1.1.25]
0.576	4.73E-02	Environmental Information Processing	Membrane Transport	ABC transporters	K02000 - glycine betaine/proline transport system ATP-binding protein proV [EC:3.6.3.32]
0.551	4.73E-02	Metabolism	Amino Acid Metabolism	Valine, leucine and isoleucine degradation	K00826 - branched-chain amino acid aminotransferase ilvE [EC:2.6.1.42]
0.495	3.71E-02	Genetic Information Processing	Replication and Repair	Mismatch repair	K03555 - DNA mismatch repair protein MutS
0.485	4.00E-02	Genetic Information Processing	Folding, Sorting and Degradation	RNA degradation	K03654 - ATP-dependent DNA helicase RecQ [EC:3.6.4.12]
-0.892	2.90E-02	Metabolism	Carbohydrate Metabolism	Amino sugar and nucleotide sugar metabolism	K00820 - glutamine---fructose-6-phosphate transaminase (isomerizing) GlmS
-0.918	4.73E-02	Environmental Information Processing	Membrane Transport	Phosphotransferase system (PTS)	K02757 - PTS system, beta-glucosides-specific IIC component PTS-Bgl-EIIC, bglF, bglP
-0.980	2.09E-02	Environmental Information Processing	Signal Transduction	MAPK signaling pathway	K17686 - P-type Cu+ transporter copA, ctpA, ATP7
-1.002	1.07E-02	Metabolism	Amino Acid Metabolism	Arginine and proline metabolism	K01478 - arginine deiminase arcA [EC:3.5.3.6]
-1.051	1.15E-02	Metabolism	Metabolism of Cofactors and Vitamins	Folate biosynthesis	K03639 - molybdenum cofactor biosynthesis protein moaA, CNX2
-1.052	3.30E-02	Unclassified	Protein families: genetic information processing	DNA repair and recombination proteins	K13531 - methylated-DNA-[protein]-cysteine S-methyltransferase adaB
-1.144	1.21E-02	Unclassified	Unclassified: metabolism	Cofactor metabolism	K03753 - molybdopterin-guanine dinucleotide biosynthesis adapter protein mobB

-1.223	2.97E-02	Metabolism	Lipid Metabolism	Glycerophospholipid metabolism	K03736 - ethanolamine ammonia-lyase small subunit eutC [EC:4.3.1.7]
-1.233	3.96E-04	Unclassified	Unclassified: metabolism	Enzymes with EC number	K10254 - oleate hydratase ohyA, sph
-1.235	1.17E-03	Unclassified	Protein families: genetic information processing	Transcription machinery	K03088 - RNA polymerase sigma-70 factor, ECF subfamily rpoE
-1.252	1.56E-03	Metabolism	Carbohydrate Metabolism	Butanoate metabolism	K00135 - succinate-semialdehyde dehydrogenase (NADP+) gabD [EC:1.2.1.16]
-1.404	2.24E-02	Unclassified	Unclassified: metabolism	Amino acid metabolism	K04026 - ethanolamine utilization protein EutL
-1.452	8.89E-03	Metabolism	Lipid Metabolism	Glycerolipid metabolism	K05879 - dihydroxyacetone kinase, C-terminal domain dhaL [EC:2.7.1.-]
-1.569	2.81E-02	Metabolism	Carbohydrate Metabolism	Butanoate metabolism	K01580 - glutamate decarboxylase gadB [EC:4.1.1.15]
-1.573	1.87E-02	Unclassified	Unclassified: metabolism	Amino acid metabolism	K04023 - ethanolamine transporter eutH
-1.579	3.27E-02	Metabolism	Metabolism of Cofactors and Vitamins	Folate biosynthesis	K03636 - sulfur-carrier protein moaD, cysO
-1.697	3.93E-02	Environmental Information Processing	Membrane Transport	ABC transporters	K16958 - L-cystine transport system permease protein tcyL
-1.756	4.81E-02	Unclassified	Unclassified: metabolism	Enzymes with EC number	K04844 - hypothetical glycosyl hydrolase ycjT
-1.827	4.56E-02	Metabolism	Carbohydrate Metabolism	Inositol phosphate metabolism	K03338 - 5-dehydro-2-deoxygluconokinase iolC [EC:2.7.1.92]
-1.913	3.42E-02	Environmental Information Processing	Membrane Transport	ABC transporters	K15770 - putative arabinogalactan oligomer transport system substrate-binding protein ganO, cycB
-1.927	2.62E-02	Environmental Information Processing	Membrane Transport	ABC transporters	K15772 - putative arabinogalactan oligomer transport system permease protein ganQ
-2.030	1.07E-02	Unclassified	Protein families: signaling and cellular processes	Transporters	K02026 - multiple sugar transport system permease protein ABC.MS.P1
-2.077	6.69E-03	Unclassified	Unclassified: metabolism	Amino acid metabolism	K04030 - ethanolimine utilization protein EutQ
-2.152	4.09E-02	Metabolism	Carbohydrate Metabolism	Propanoate metabolism	K00140 - malonate-semialdehyde dehydrogenase (acetylating) / methylmalonate-semialdehyde dehydrogenase iolA [EC:1.2.1.18 1.2.1.27]
-2.164	3.96E-02	Unclassified	Protein families: signaling and cellular processes	Transporters	K02025 - multiple sugar transport system permease protein ABS.MS.P
-2.205	9.81E-03	Metabolism	Metabolism of Cofactors and Vitamins	Porphyrin and chlorophyll metabolism	K02224 - cobyrinic acid a,c-diamide synthase cobB-cbiA [EC:6.3.5.9 6.3.5.11]

-2.299	2.09E-02	Metabolism	Metabolism of Cofactors and Vitamins	Porphyrin and chlorophyll metabolism	K02227 - adenosylcobinamide-phosphate synthase cbiB, cobD [EC:6.3.1.10]
-2.313	4.81E-02	Cellular Processes	Cell Motility	Flagellar assembly	K02396 - flagellar hook-associated protein 1 FlgK
-2.401	9.74E-03	Cellular Processes	Cell Motility	Flagellar assembly	K02412 - flagellum-specific ATP synthase fliI [EC:3.6.3.14]
-2.418	4.81E-02	Metabolism	Metabolism of Cofactors and Vitamins	Porphyrin and chlorophyll metabolism	K02226 - alpha-ribazole phosphatase cobC, php [EC:3.1.3.73]
-2.446	2.09E-02	Cellular Processes	Cell Motility	Bacterial chemotaxis	K02410 - flagellar motor switch protein FliG
-2.492	2.06E-02	Environmental Information Processing	Membrane Transport	ABC transporters	K17320 - putative aldouronate transport system permease protein lplC
-2.495	4.20E-02	Cellular Processes	Cell Motility	Flagellar assembly	K02397 - flagellar hook-associated protein 3 FlgL
-2.502	3.45E-02	Unclassified	-	-	K09770 - uncharacterized protein
-2.535	2.72E-02	Environmental Information Processing	Signal Transduction	Two-component system	K03407 - two-component system, chemotaxis family, sensor kinase CheA
-2.597	3.45E-02	Cellular Processes	Cell Motility	Flagellar assembly	K02387 - flagellar basal-body rod protein FlgB
-2.628	3.82E-02	Cellular Processes	Cell Motility	Flagellar assembly	K02388 - flagellar basal-body rod protein FlgC
-2.893	1.14E-02	Environmental Information Processing	Membrane Transport	ABC transporters	K17319 - putative aldouronate transport system permease protein lplB
-2.984	3.74E-02	Cellular Processes	Cell Motility	Flagellar assembly	K02408 - flagellar hook-basal body complex protein FliE
-3.300	9.81E-03	Cellular Processes	Cell Motility	Flagellar assembly	K02421 - flagellar biosynthetic protein FliR
