# Supplementary material for: Unravelling the Molecular Mechanisms Underlying the Protective Effect of Lactate on the High-Pressure Resistance of Listeria monocytogenes
Source: Biomolecules. 2021 Apr 30;11(5):677. doi: 10.3390/biom11050677 (PMC8147161; doi:10.3390/biom11050677)
Supplement: Supplementary file 1 [file biomolecules-11-00677-s001.zip › biomolecules-1111984-proof-suppl/supplementary table 8.pdf]

Table S8. List of KEGG Orthology (KO) genes differentially (FDR<0.05) expressed in the *L. monocytogenes* strain EGDe in pressurized samples without and with lactate. Positive Log2 fold change indicate genes more abundant in samples with lactate.

Log2 Fold Change	FDR	KEGG annotation at level 1	KEGG annotation at level 2	KEGG pathway	KEGG Orthology (KO) genes
4.939	3.22E-03	Cellular Processes	Cell Motility	Flagellar assembly	K02421 - flagellar biosynthetic protein FliR
4.650	5.72E-03	Cellular Processes	Cell Motility	Flagellar assembly	K02392 - flagellar basal-body rod protein FlgG
4.616	3.11E-03	Cellular Processes	Cell Motility	Flagellar assembly	K02387 - flagellar basal-body rod protein FlgB
4.587	3.36E-03	Cellular Processes	Cell Motility	Flagellar assembly	K02408 - flagellar hook-basal body complex protein FliE
4.477	2.96E-03	Environmental Information Processing	Signal Transduction	Two-component system	K03407 - two-component system, chemotaxis family, sensor kinase CheA
4.441	3.43E-03	Cellular Processes	Cell Motility	Flagellar assembly	K02390 - flagellar hook protein FlgE
4.429	2.96E-03	Environmental Information Processing	Membrane Transport	ABC transporters	K17319 - putative aldouronate transport system permease protein (ABC transporters) lplB
4.317	9.43E-03	Brite Hierarchies	Protein families: signaling and cellular processes	Bacterial motility proteins	K02404 - flagellar biosynthesis protein FlhF
4.312	2.96E-03	Cellular Processes	Cell Motility	Flagellar assembly	K02396 - flagellar hook-associated protein 1 FlgK
4.307	4.17E-03	Cellular Processes	Cell Motility	Flagellar assembly	K02388 - flagellar basal-body rod protein FlgC
4.253	3.36E-03	Cellular Processes	Cell Motility	Flagellar assembly	K02397 - flagellar hook-associated protein 3 FlgL
4.208	4.07E-03	Cellular Processes	Cell Motility	Flagellar assembly	K02409 - flagellar M-ring protein FliF
4.171	2.96E-03	Cellular Processes	Cell Motility	Flagellar assembly	K02412 - flagellum-specific ATP synthase fliI [EC:3.6.3.14]
4.136	2.99E-02	Cellular Processes	Cell Motility	Flagellar assembly	K02400 - flagellar biosynthesis protein FlhA
4.080	7.23E-03	Cellular Processes	Cell Motility	Bacterial chemotaxis	K02416 - flagellar motor switch protein FliM
4.032	2.57E-02	Unclassified	Unclassified: genetic information processing	Replication and repair	K07467 - phage replication initiation protein rstA1
4.016	2.96E-03	Metabolism	Metabolism of Cofactors and Vitamins	Porphyrin and chlorophyll metabolism	K02226 - alpha-ribazole phosphatase cobC, phpB [EC:3.1.3.73]
3.995	3.31E-03	Brite Hierarchies	Protein families: signaling and cellular processes	Secretion system	K02246 - competence protein ComGD
3.956	6.45E-03	Brite Hierarchies	Protein families: signaling and cellular processes	Transporters	K11203 - PTS system, fructose-specific IIC-like component, PTS-Fru2-EIIC
3.943	5.17E-03	Cellular Processes	Cell Motility	Bacterial chemotaxis	K02410 - flagellar motor switch protein FliG
3.930	4.07E-03	Unclassified	-	-	K09770 - uncharacterized protein
3.914	5.39E-03	Cellular Processes	Cell Motility	Bacterial chemotaxis	K02417 - flagellar motor switch protein FliN/FliY

3.910	2.99E-02	Environmental Information Processing	Signal Transduction	Two-component system	K02556 - chemotaxis protein MotA
3.853	3.26E-03	Metabolism	Metabolism of Cofactors and Vitamins	Porphyrin and chlorophyll metabolism	K02227 - adenosylcobinamide-phosphate synthase cbiB, cobD [EC:6.3.1.10]
3.842	3.73E-03	Environmental Information Processing	Membrane Transport	ABC transporters	K17320 - putative aldouronate transport system permease protein lplC
3.832	8.92E-03	Environmental Information Processing	Signal Transduction	Two-component system	K03413 - two-component system, chemotaxis family, response regulator CheY
3.806	1.54E-02	Metabolism	Carbohydrate Metabolism	Propanoate metabolism	K00140 - malonate-semialdehyde dehydrogenase (acetylating) / methylmalonate-semialdehyde dehydrogenase [EC:1.2.1.18 1.2.1.27], iolA
3.719	5.39E-03	Brite Hierarchies	Protein families: signaling and cellular processes	Transporters	K02025 - multiple sugar transport system permease protein ABC.MS.P
3.692	3.85E-03	Metabolism	Carbohydrate Metabolism	Inositol phosphate metabolism	K03337 - 5-deoxy-glucuronate isomerase iolB [EC:5.3.1.-]
3.682	2.96E-03	Metabolism	Metabolism of Cofactors and Vitamins	Porphyrin and chlorophyll metabolism	K02224 - cobyrinic acid a,c-diamide synthase cobB-cbiA [EC:6.3.5.9 6.3.5.11]
3.600	3.09E-02	Cellular Processes	Cell Motility	Flagellar assembly	K02389 - flagellar basal-body rod modification protein FlgD
3.524	6.61E-03	Environmental Information Processing	Signal Transduction	Two-component system	K01546 - K+-transporting ATPase ATPase A chain KdpA [EC:3.6.3.12]
3.442	3.85E-03	Metabolism	Metabolism of Cofactors and Vitamins	Porphyrin and chlorophyll metabolism	K06042 - precorrin-8X methylmutase cobH-cbiC [EC:5.4.1.2]
3.436	6.76E-03	Metabolism	Metabolism of Cofactors and Vitamins	Porphyrin and chlorophyll metabolism	K02191 - cobalt-precorrin-7 (C15)-methyltransferase cbiT [EC:2.1.1.196]
3.427	3.26E-03	Environmental Information Processing	Membrane Transport	ABC transporters	K16959 - L-cystine transport system permease protein tcyM
3.414	2.02E-02	Cellular Processes	Cell Motility	Flagellar assembly	K02411 - flagellar assembly protein FliH
3.293	7.11E-03	Environmental Information Processing	Membrane Transport	ABC transporters	K15771 - putative arabinogalactan oligomer transport system permease protein ganP
3.271	6.38E-03	Environmental Information Processing	Membrane Transport	ABC transporters	K15770 - putative arabinogalactan oligomer transport system substrate-binding protein cycB, ganO
3.255	4.33E-03	Environmental Information Processing	Membrane Transport	ABC transporters	K16958 - L-cystine transport system permease protein tcyL
3.244	1.03E-02	Metabolism	Metabolism of Cofactors and Vitamins	Thiamine metabolism	K00878 - hydroxyethylthiazole kinase thiM [EC:2.7.1.50]
3.240	3.90E-02	Metabolism	Amino Acid Metabolism	Alanine, aspartate and glutamate metabolism	K00278 - L-aspartate oxidase nadB [EC:1.4.3.16]
3.230	4.33E-03	Environmental Information Processing	Membrane Transport	ABC transporters	K15772 - putative arabinogalactan oligomer transport system permease protein ganQ
3.219	3.90E-02	Metabolism	Metabolism of Cofactors and Vitamins	Thiamine metabolism	K03707 - thiaminase (transcriptional activator TenA) [EC:3.5.99.2]

3.211	1.04E-02	Metabolism	Metabolism of Cofactors and Vitamins	Porphyrin and chlorophyll metabolism	K02233 - adenosylcobinamide-GDP ribazoletransferase cobS, cobV [EC:2.7.8.26]
3.155	7.94E-03	Metabolism	Metabolism of Cofactors and Vitamins	Porphyrin and chlorophyll metabolism	K05936 - precorrin-4 C11-methyltransferase cobM, cbiF [EC:2.1.1.133]
3.144	4.49E-03	Metabolism	Carbohydrate Metabolism	Inositol phosphate metabolism	K03338 - 5-dehydro-2-deoxygluconokinase iolC [EC:2.7.1.92]
3.134	1.48E-02	Metabolism	Metabolism of Cofactors and Vitamins	Porphyrin and chlorophyll metabolism	K05934 - precorrin-3B C17-methyltransferase cobJ, cbiH [EC:2.1.1.131]
3.133	1.26E-02	Metabolism	Metabolism of Cofactors and Vitamins	Porphyrin and chlorophyll metabolism	K02188 - cobalt-precorrin-5B (C1)-methyltransferase cbiD [EC:2.1.1.195]
3.103	4.08E-03	Metabolism	Metabolism of Cofactors and Vitamins	Porphyrin and chlorophyll metabolism	K02190 - sirohydrochlorin cobaltochelatase cbiK [EC:4.99.1.3]
3.100	3.22E-03	Brite Hierarchies	Protein families: signaling and cellular processes	Transporters	K02026 - multiple sugar transport system permease protein ABS.MS.P1
3.100	4.33E-03	Brite Hierarchies	Protein families: signaling and cellular processes	Transporters	K10974 - cytosine permease codB
3.073	6.61E-03	Unclassified	-	-	K04844 - hypothetical glycosyl hydrolase ycjT
3.057	9.49E-03	Environmental Information Processing	Membrane Transport	ABC transporters	K02007 - cobalt/nickel transport system permease protein cbiM
3.017	7.54E-03	Environmental Information Processing	Signal Transduction	Two-component system	K07720 - two-component system, response regulator YesN
2.859	3.85E-03	Metabolism	Metabolism of Cofactors and Vitamins	Nicotinate and nicotinamide metabolism	K03517 - quinolinate synthase nadA [EC:2.5.1.72]
2.826	3.85E-03	Unclassified	-	-	K09703 - uncharacterized protein
2.824	2.02E-02	Environmental Information Processing	Membrane Transport	ABC transporters	K17318 - putative aldouronate transport system substrate-binding protein lplA
2.799	8.56E-03	Metabolism	Energy Metabolism	Oxidative phosphorylation	K02111 - F-type H+-transporting ATPase subunit alpha [EC:3.6.3.14]
2.748	4.88E-03	Brite Hierarchies	Protein families: signaling and cellular processes	Transporters	K08151 - MFS transporter, DHA1 family, tetracycline resistance protein tetA
2.746	5.87E-03	Metabolism	Carbohydrate Metabolism	Butanoate metabolism	K01580 - glutamate decarboxylase gadB, gadA, GAD [EC:4.1.1.15]
2.743	1.40E-02	Environmental Information Processing	Membrane Transport	ABC transporters	K02009 - cobalt transport protein cbiN
2.739	4.33E-03	Metabolism	Metabolism of Cofactors and Vitamins	Nicotinate and nicotinamide metabolism	K00767 - nicotinate-nucleotide pyrophosphorylase (carboxylating) nadC [EC:2.4.2.19]
2.706	4.22E-02	Brite Hierarchies	Protein families: signaling and cellular processes	Secretion system	K02247 - competence protein ComGE
2.672	2.96E-03	Unclassified	Unclassified: metabolism	Amino acid metabolism	K04028 - ethanolamine utilization protein EutN

2.653	1.20E-02	Metabolism	Metabolism of Cofactors and Vitamins	Porphyrin and chlorophyll metabolism	K03394 - precorrin-2/cobalt-factor-2 C20-methyltransferase cobI-cbiL [EC:2.1.1.130 2.1.1.151]
2.643	1.72E-02	Metabolism	Amino Acid Metabolism	Arginine and proline metabolism	K00145 - N-acetyl-gamma-glutamyl-phosphate reductase argC [EC:1.2.1.38]
2.606	1.05E-02	Metabolism	Amino Acid Metabolism	Arginine and proline metabolism	K00620 - glutamate N-acetyltransferase / amino-acid N-acetyltransferase argJ [EC:2.3.1.35 2.3.1.1]
2.575	5.24E-03	Metabolism	Energy Metabolism	Nitrogen metabolism	K00265 - glutamate synthase (NADPH/NADH) large chain gltB [EC:1.4.1.13 1.4.1.14]
2.524	1.78E-02	Metabolism	Amino Acid Metabolism	Arginine and proline metabolism	K10536 - agmatine deiminase aguA [EC:3.5.3.12]
2.506	1.20E-02	Metabolism	Amino Acid Metabolism	Phenylalanine, tyrosine and tryptophan biosynthesis	K01658 - anthranilate synthase component II trpG [EC:4.1.3.27]
2.477	9.49E-03	Metabolism	Amino Acid Metabolism	Phenylalanine, tyrosine and tryptophan biosynthesis	K00766 - anthranilate phosphoribosyltransferase trpD [EC:2.4.2.18]
2.475	1.37E-02	Metabolism	Amino Acid Metabolism	Arginine and proline metabolism	K00818 - acetylornithine aminotransferase argD [EC:2.6.1.11]
2.466	3.85E-03	Environmental Information Processing	Membrane Transport	ABC transporters	K02038 - phosphate transport system permease protein pstA
2.465	1.05E-02	Metabolism	Lipid Metabolism	Glycerolipid metabolism	K01699 - propanediol dehydratase large subunit pduC [EC:4.2.1.28]
2.420	3.26E-03	Unclassified	Unclassified: metabolism	Amino acid metabolism	K04030 - ethanolamine utilization protein EutQ
2.409	2.54E-02	Cellular Processes	Cell Motility	Flagellar assembly	K02419 - flagellar biosynthetic protein FliP
2.377	2.87E-02	Metabolism	Energy Metabolism	Nitrogen metabolism	K00266 - glutamate synthase (NADPH/NADH) small chain gltD [EC:1.4.1.13 1.4.1.14]
2.352	1.03E-02	Metabolism	Glycan Biosynthesis and Metabolism	Other glycan degradation	K01191 - alpha-mannosidase MAN2C1 [EC:3.2.1.24]
2.338	1.81E-02	Unclassified	-	-	K09704 - uncharacterized protein
2.331	3.81E-02	Metabolism	Carbohydrate Metabolism	Glyoxylate and dicarboxylate metabolism	K00123 - formate dehydrogenase, alpha subunit fdoG, fdhF, fdwA [EC:1.2.1.2]
2.324	4.80E-02	Brite Hierarchies	Protein families: genetic information processing	Ribosome biogenesis	K00783 - 23S rRNA (pseudouridine1915-N3)-methyltransferase, rlmH
2.295	3.31E-03	Environmental Information Processing	Membrane Transport	ABC transporters	K02036 - phosphate transport system ATP-binding protein pstB [EC:3.6.3.27]
2.286	1.87E-03	Metabolism	Lipid Metabolism	Glycerolipid metabolism	K05879 - dihydroxyacetone kinase, C-terminal domain dhaL [EC:2.7.1.-]
2.268	3.73E-02	Metabolism	Lipid Metabolism	Glycerolipid metabolism	K13919 - propanediol dehydratase medium subunit pduD [EC:4.2.1.28]
2.249	4.33E-03	Metabolism	Metabolism of Cofactors and Vitamins	Thiamine metabolism	K00788 - thiamine-phosphate pyrophosphorylase thiE [EC:2.5.1.3]
2.243	3.36E-03	Environmental Information Processing	Membrane Transport	ABC transporters	K02015 - iron complex transport system permease protein ABC.FEV.P
2.214	3.81E-02	Metabolism	Energy Metabolism	Sulfur metabolism	K01760 - cystathionine beta-lyase metC [EC:4.4.1.8]
2.156	3.26E-03	Unclassified	Unclassified: metabolism	Amino acid metabolism	K04023 - ethanolamine transporter eutH

2.154	2.08E-02	Environmental Information Processing	Signal Transduction	Two-component system	K02406 - flagellin FliC
2.153	5.66E-03	Brite Hierarchies	Protein families: signaling and cellular processes	Secretion system	K02244 - competence protein ComGB
2.143	7.94E-03	Environmental Information Processing	Membrane Transport	Phosphotransferase system (PTS)	K02757 - PTS system, beta-glucosides-specific IIC component, PTS-Bgl-EIIC, bglF, bglP
2.048	8.85E-03	Metabolism	Metabolism of Cofactors and Vitamins	Folate biosynthesis	K03636 - molybdopterin synthase sulfur carrier subunit moaD, cysO
2.044	2.69E-02	Unclassified	Unclassified: metabolism	Amino acid metabolism	K04024 - ethanolamine utilization protein EutJ
2.037	3.34E-02	Metabolism	Carbohydrate Metabolism	Pentose phosphate pathway	K01808 - ribose 5-phosphate isomerase B, rpiB
2.025	3.90E-02	Metabolism	Amino Acid Metabolism	Phenylalanine, tyrosine and tryptophan biosynthesis	K01817 - phosphoribosylanthranilate isomerase trpF [EC:5.3.1.24]
2.005	3.09E-02	Metabolism	Metabolism of Cofactors and Vitamins	Thiamine metabolism	K00941 - hydroxymethylpyrimidine/phosphomethylpyrimidine kinase thiD [EC:2.7.1.49 2.7.4.7]
1.999	3.90E-02	Brite Hierarchies	Protein families: signaling and cellular processes	Transporters	K18967 - diguanylate cyclase dge1
1.942	3.22E-03	Metabolism	Amino Acid Metabolism	Histidine metabolism	K00765 - ATP phosphoribosyltransferase hisG [EC:2.4.2.17]
1.930	4.57E-02	Metabolism	Amino Acid Metabolism	Cysteine and methionine metabolism	K00549 - 5-methyltetrahydropteroyltriglutamate--homocysteine methyltransferase metE [EC:2.1.1.14]
1.864	2.41E-02	Environmental Information Processing	Membrane Transport	ABC transporters	K11051 - multidrug/hemolysin transport system permease protein cylB
1.838	4.05E-02	Metabolism	Carbohydrate Metabolism	Propanoate metabolism	K13923 - phosphotransacylase pduL
1.831	3.31E-03	Unclassified	Unclassified: metabolism	Cofactor metabolism	K03753 - multiple sugar transport system permease protein mobB
1.824	3.22E-03	Metabolism	Metabolism of Cofactors and Vitamins	Porphyrin and chlorophyll metabolism	K04032 - ethanolamine utilization cobalamin adenosyltransferase eutT [EC:2.5.1.17]
1.798	1.19E-02	Brite Hierarchies	Protein families: signaling and cellular processes	Transporters	K08161 - MFS transporter, DHA1 family, multidrug resistance protein mdtG
1.787	4.07E-02	Brite Hierarchies	Protein families: signaling and cellular processes	Transporters	K02027 - multiple sugar transport system substrate-binding protein ABC.MS.S
1.778	3.04E-02	Brite Hierarchies	Protein families: genetic information processing	Transcription factors	K03481 - RpiR family transcriptional regulator, glv operon transcriptional regulator, glvR
1.773	3.73E-02	Brite Hierarchies	Protein families: genetic	Transcription factors	K02538 - activator of the mannose operon, transcriptional antiterminator manR

			information processing		
1.740	3.90E-02	Unclassified	Unclassified: genetic information processing	Replication and repair	K07483 - transposase
1.731	4.80E-02	Brite Hierarchies	Protein families: signaling and cellular processes	Transporters	K11741 - quaternary ammonium compound-resistance protein SugE
1.713	5.39E-03	Brite Hierarchies	Protein families: signaling and cellular processes	Transporters	K07507 - putative Mg2+ transporter-C (MgtC) family protein mgtC
1.678	1.96E-02	Environmental Information Processing	Membrane Transport	ABC transporters	K02006 - cobalt/nickel transport system ATP-binding protein cbiO
1.648	4.63E-02	Metabolism	Lipid Metabolism	Glycerolipid metabolism	K13921 - 1-propanol dehydrogenase pduQ
1.628	2.96E-03	Brite Hierarchies	Protein families: signaling and cellular processes	Transporters	K05567 - multicomponent Na+:H+ antiporter subunit C mnhC, mrpC
1.611	4.73E-02	Environmental Information Processing	Membrane Transport	Phosphotransferase system (PTS)	K02761 - PTS system, cellobiose-specific IIC component, PTS-Cel-EIIC, celB, chbC
1.602	2.96E-03	Unclassified	Unclassified: metabolism	Amino acid metabolism	K04026 - ethanolamine utilization protein EutL
1.588	1.26E-02	Metabolism	Amino Acid Metabolism	Arginine and proline metabolism	K01478 - arginine deiminase arcA [EC:3.5.3.6]
1.516	2.54E-02	Metabolism	Amino Acid Metabolism	Arginine and proline metabolism	K01426 - amidase yhgE [EC:3.5.1.4]
1.480	1.21E-02	Metabolism	Metabolism of Cofactors and Vitamins	Folate biosynthesis	K03638 - molybdopterin adenylyltransferase moaB
1.460	1.02E-02	Brite Hierarchies	Protein families: genetic information processing	DNA repair and recombination factors	K03547 - DNA repair protein SbcD/Mre11
1.412	4.41E-03	Environmental Information Processing	Membrane Transport	ABC transporters	K05845 - osmoprotectant transport system substrate-binding protein opuC
1.379	3.71E-02	Metabolism	Carbohydrate Metabolism	Amino sugar and nucleotide sugar metabolism	K00820 - glutamine---fructose-6-phosphate transaminase (isomerizing) GlmS, GFPT
1.377	9.43E-03	Unclassified	Unclassified: metabolism	Enzymes with EC numbers	K01531 - P-type Mg2+ transporter mgtA, mgtB
1.300	9.49E-03	Unclassified	Unclassified: genetic information processing	Replication and repair	K06909 - phage terminase large subunit xtmB
1.262	4.73E-02	Genetic Information Processing	Folding, Sorting and Degradation	Sulfur relay system	K11996 - adenylyltransferase and sulfurtransferase MOCS3, UBA4
1.233	4.33E-03	Metabolism	Lipid Metabolism	Glycerophospholipid metabolism	K03736 - ethanolamine ammonia-lyase small subunit EutC [EC:4.3.1.7]
1.112	4.08E-03	Brite Hierarchies	Protein families: signaling and	Transporters	K05565 - multicomponent Na+:H+ antiporter subunit A mnhA, mrpA

			cellular processes		
1.081	3.69E-02	Metabolism	Carbohydrate Metabolism	Amino sugar and nucleotide sugar metabolism	K12410 - NAD-dependent deacetylase npdA [EC:3.5.1.-]
0.989	4.91E-02	Brite Hierarchies	Protein families: signaling and cellular processes	Transporters	K05570 - multicomponent Na+:H+ antiporter subunit F, mnhF, mrpF
0.937	1.26E-02	Metabolism	Amino Acid Metabolism	Phenylalanine, tyrosine and tryptophan biosynthesis	K06208 - chorismate mutase aroH [EC:5.4.99.5]
0.918	3.26E-03	Brite Hierarchies	Protein families: signaling and cellular processes	Transporters	K05020 - glycine betaine transporter opuD, betL
0.890	1.04E-02	Metabolism	Metabolism of Terpenoids and Polyketides	Carotenoid biosynthesis	K10027 - phytoene desaturase crtl [EC:1.3.99.26 1.3.99.28 1.3.99.29 1.3.99.31]
0.880	2.99E-02	Brite Hierarchies	Protein families: signaling and cellular processes	Transporters	K04759 - ferrous iron transport protein B, feoB
0.877	4.86E-02	Metabolism	Amino Acid Metabolism	Cysteine and methionine metabolism	K08969 - aminotransferase mtnE, mtnV [EC:2.6.1.-]
0.839	3.85E-03	Metabolism	Metabolism of Cofactors and Vitamins	Ubiquinone and other terpenoid-quinone biosynthesis	K02549 - O-succinylbenzoate synthase menC [EC:4.2.1.113]
0.829	2.80E-02	Metabolism	Metabolism of Cofactors and Vitamins	Nicotinate and nicotinamide metabolism	K08281 - nicotinamidase/pyrazinamidase pncA [EC:3.5.1.19 3.5.1.-]
0.781	1.48E-02	Brite Hierarchies	Protein families: genetic information processing	Transcription factors	K10778 - AraC family transcriptional regulator, regulatory protein of adaptative response / methylated-DNA-[protein]-cysteine methyltransferase [EC:2.1.1.63], ada
0.779	3.90E-02	Environmental Information Processing	Membrane Transport	ABC transporters	K02013 - iron complex transport system ATP-binding protein ABC.FEV.A [EC:3.6.3.34]
0.679	3.71E-02	Unclassified	Unclassified: genetic information processing	Replication and repair	K07457 - endonuclease III related protein
0.628	4.62E-03	Environmental Information Processing	Membrane Transport	ABC transporters	K02072 - D-methionine transport system permease protein metI
0.623	3.70E-02	Brite Hierarchies	Protein families: signaling and cellular processes	Transporters	K07238 - zinc transporter, ZIP family, TC.ZIP, zupT, ZRT3, ZIP2
0.544	8.93E-03	Brite Hierarchies	Protein families: genetic information processing	Translation factors	K06158 - ATP-binding cassette, subfamily F, member 3 ABCF3
-0.399	4.91E-02	Metabolism	Nucleotide Metabolism	Purine metabolism	K00951 - GTP pyrophosphokinase relA [EC:2.7.6.5]

-0.422	2.02E-02	Brite Hierarchies	Protein families: genetic information processing	Transcription factors	K03486 - GntR family transcriptional regulator, trehalose operon transcriptional repressor treR
-0.493	4.22E-02	Metabolism	Lipid Metabolism	Glycerolipid metabolism	K00901 - diaglycerol kinase (ATP), DgkA
-0.513	4.91E-02	Metabolism	Metabolism of Cofactors and Vitamins	Vitamin B6 metabolism	K08681 - glutamine amidotransferase pdxT, pdx2 [EC:2.6.-.-]
-0.549	4.26E-02	Brite Hierarchies	Protein families: metabolism	Peptidases and inhibitors	K01419 - ATP-dependent HslUV protease, peptidase subunit HslV, ClpQ
-0.560	1.02E-02	Unclassified	-	-	K07023 - putative hydrolases of HD superfamily
-0.579	4.39E-02	Unclassified	Unclassified: genetic information processing	Transcription	K06959 - protein Tex
-0.584	1.08E-02	Brite Hierarchies	Protein families: genetic information processing	Transcription factors	K07738 - transcriptional repressor NrdR
-0.617	4.63E-02	Unclassified	-	-	K01421 - putative membrane protein yhgE
-0.636	1.54E-02	Metabolism	Metabolism of Other Amino Acids	D-Alanine metabolism	K01921 - D-alanine-D-alanine ligase ddL [EC:6.3.2.4]
-0.644	1.54E-02	Genetic Information Processing	Replication and Repair	Mismatch repair	K03572 - DNA mismatch repair protein MutL
-0.646	3.73E-02	Metabolism	Metabolism of Cofactors and Vitamins	Porphyrin and chlorophyll metabolism	K01772 - ferrochelatase hemH [EC:4.99.1.1]
-0.650	3.98E-03	Metabolism	Carbohydrate Metabolism	Pyruvate metabolism	K01759 - lactoylglutathione lyase gloA [EC:4.4.1.5]
-0.657	2.36E-02	Metabolism	Lipid Metabolism	Fatty acid biosynthesis	K02372 - 3-hydroxyacyl-[acyl-carrier-protein] dehydratase FabZ [EC:4.2.1.59]
-0.660	1.91E-02	Brite Hierarchies	Protein families: genetic information processing	Transcription factors	K03402 - transcriptional regulator of arginine metabolism argR, ahrC
-0.706	7.23E-03	Metabolism	Metabolism of Cofactors and Vitamins	Riboflavin metabolism	K11753 - riboflavin kinase / FMN adenylyltransferase ribF [EC:2.7.1.26 2.7.7.2]
-0.714	3.73E-02	Brite Hierarchies	Protein families: metabolism	Peptidoglycan biosynthesis and degradation proteinns	K01448 - N-acetylmuramoyl-L-alanine amidase, amiABC
-0.721	1.05E-02	Unclassified	-	-	K05937 - uncharacterized protein
-0.727	4.41E-03	Metabolism	Metabolism of Terpenoids and Polyketides	Zeatin biosynthesis	K00791 - tRNA dimethylallyltransferase miaA [EC:2.5.1.75]
-0.729	1.95E-02	Brite Hierarchies	Protein families: genetic information processing	Translation factors	K02838 - ribosome recycling factor frr, MRRF, RRF
-0.729	1.99E-02	Cellular Processes	Cellular community - prokaryotes	Quorum sensing	K02035 - peptide/nickel transport system substrate-binding protein ABC.PE.S

-0.729	5.72E-03	Unclassified	Unclassified: signaling and cellular processes	Cell growth	K06346 - spoIIIJ-associated protein jag
-0.756	4.71E-02	Environmental Information Processing	Membrane Transport	ABC transporters	K09812 - cell division transport system ATP-binding protein ftsE
-0.780	1.95E-02	Brite Hierarchies	Protein families: genetic information processing	Transcription factors	K07723 - CopG family transcriptional regulator / antitoxin EndoAI, ndoAI
-0.787	4.86E-02	Environmental Information Processing	Signal Transduction	Two-component system	K19692 - osmolarity two-component system, sensor histidine kinase TcsA
-0.821	2.50E-02	Brite Hierarchies	Protein families: metabolism	Peptidases and inhibitors	K08602 - oligoendopeptidase F, pepF
-0.823	3.75E-02	Brite Hierarchies	Protein families: genetic information processing	DNA repair and recombination factors	K03502 - DNA polymerase V, umuC
-0.827	1.05E-02	Metabolism	Lipid Metabolism	Glycerolipid metabolism	K03429 - 1,2-diacylglycerol 3-glucosyltransferase ugtP [EC:2.4.1.157]
-0.863	2.54E-02	Unclassified	Unclassified: genetic information processing	Translation	K07571 - S1 RNA binding domain protein
-0.867	1.40E-02	Brite Hierarchies	Protein families: metabolism	Peptidases and inhibitors	K01258 - tripeptide aminopeptidase pepT [EC:3.4.11.4]
-0.868	3.04E-02	Brite Hierarchies	Protein families: genetic information processing	Transfer RNA biogenesis	K11991 - tRNA(adenine34) deaminase, tadA
-0.872	2.69E-02	Unclassified	Unclassified: metabolism	Enzymes with EC numbers	K07304 - peptide-methionine (S)-S-oxide reductase, msrA
-0.875	4.46E-02	Metabolism	Carbohydrate Metabolism	Pyruvate metabolism	K02160 - acetyl-CoA carboxylase biotin carboxyl carrier protein accB, bccP
-0.889	4.44E-02	Brite Hierarchies	Protein families: signaling and cellular processes	Exosome	K02503 - histidine triad (HIT) family protein
-0.900	3.81E-02	Environmental Information Processing	Signal Transduction	Calcium signaling pathway	K16896 - two pore calcium channel protein 1, TPCN1
-0.908	6.45E-03	Environmental Information Processing	Signal Transduction	Two-component system	K07775 - two-component system, OmpR family, response regulator ResD
-0.914	1.05E-02	Brite Hierarchies	Protein families: genetic information processing	DNA replication proteins	K03168 - DNA topoisomerase I topA
-0.919	1.28E-02	Unclassified	Unclassified: metabolism	Enzymes with EC numbers	K03734 - FAD:protein FMN transferase apbE
-0.932	6.76E-03	Metabolism	Glycan Biosynthesis and Metabolism	Peptidoglycan biosynthesis	K07260 - D-alanyl-D-alanine carboxypeptidase VanY [EC:3.4.16.4]

-0.946	4.80E-02	Genetic Information Processing	Translation	Ribosome	K02970 - small subunit ribosomal protein S21
-0.950	3.71E-02	Environmental Information Processing	Signal Transduction	Two-component system	K07707 - two-component system, AgrA family, response regulator AgrA
-0.968	2.94E-02	Genetic Information Processing	Replication and Repair	Nucleotide excision repair	K03701 - excinuclease ABC subunit A, uvrA
-0.973	1.17E-02	Genetic Information Processing	Translation	Ribosome	K02939 - large subunit ribosomal protein L9
-0.982	1.96E-02	Unclassified	Unclassified: metabolism	Enzymes with EC numbers	K04068 - anaerobic ribonucleoside-triphosphate reductase activating protein, nrdG
-0.985	6.45E-03	Unclassified	Unclassified: metabolism	Enzymes with EC numbers	K00537 - arsenate reductase (glutaredoxin) arsC
-0.986	2.28E-02	Brite Hierarchies	Protein families: genetic information processing	Transcription factors	K02443 - glycerol uptake operon antiterminator glpP
-0.987	1.04E-02	Genetic Information Processing	Translation	Aminoacyl-tRNA biosynthesis	K04567 - lysyl-tRNA synthetase, class II lysS [EC:6.1.1.6]
-1.000	2.96E-03	Genetic Information Processing	Replication and Repair	DNA replication	K03469 - ribonuclease HI [EC:3.1.26.4]
-1.026	4.63E-02	Genetic Information Processing	Folding, Sorting and Degradation	RNA degradation	K04043 - molecular chaperone DnaK
-1.095	4.33E-03	Metabolism	Nucleotide Metabolism	Pyrimidine metabolism	K00761 - uracil phosphoribosyltransferase upp [EC:2.4.2.9]
-1.106	9.92E-03	Unclassified	Unclassified: signaling and cellular processes	Cell growth	K06412 - stage V sporulation protein G, spoVG
-1.117	3.26E-03	Unclassified	-	-	K09979 - uncharacterized protein
-1.208	3.26E-03	Metabolism	Nucleotide Metabolism	Purine metabolism	K00527 - ribonucleoside-triphosphate reductase rtpR [EC:1.17.4.2]
-1.242	1.21E-02	Brite Hierarchies	Protein families: genetic information processing	Chaperones and folding catalysts	K04078 - chaperonin GroES
-1.307	5.89E-03	Brite Hierarchies	Protein families: genetic information processing	Chaperones and folding catalysts	K07533 - foldase protein PrsA
-1.323	1.44E-02	Genetic Information Processing	Translation	Ribosome	K02914 - large subunit ribosomal protein L34
-1.528	4.05E-02	Environmental Information Processing	Signal Transduction	Two-component system	K04751 - nitrogen regulatory protein P-II 1, GlnB
