# Supplementary material for: Unravelling the Molecular Mechanisms Underlying the Protective Effect of Lactate on the High-Pressure Resistance of Listeria monocytogenes
Source: Biomolecules. 2021 Apr 30;11(5):677. doi: 10.3390/biom11050677 (PMC8147161; doi:10.3390/biom11050677)
Supplement: Supplementary file 1 [file biomolecules-11-00677-s001.zip › biomolecules-1111984-proof-suppl/supplementary table 9.pdf]

Table S9. List of KEGG Orthology (KO) genes differentially (FDR<0.05) expressed in the *L. monocytogenes* strain EGDe in samples with lactate non-pressurized and pressurized. Positive Log2 fold change indicate genes more abundant in pressurized samples.

Log2 Fold Change	FDR	KEGG annotation at level 1	KEGG annotation at level 2	KEGG pathway	KEGG Orthology (KO) genes
1.876	3.30E-02	Metabolism	Metabolism of other amino acids	D-Alanine metabolism	K14188 - D-alanine--poly(phosphoribitol) ligase subunit 2 DltC
1.793	3.99E-03	Metabolism	Amino Acid Metabolism	Valine, leucine and isoleucine biosynthesis	K01687 - dihydroxy-acid dehydratase ilvD [EC:4.2.1.9]
1.643	4.69E-04	Metabolism	Metabolism of Terpenoids and Polyketides	Terpenoid backbone biosynthesis	K00099 - 1-deoxy-D-xylulose-5-phosphate reductoisomerase dxr [EC:1.1.1.267]
1.414	3.81E-02	Metabolism	Amino Acid Metabolism	Valine, leucine and isoleucine biosynthesis	K00052 - 3-isopropylmalate dehydrogenase leuB [EC:1.1.1.85]
1.080	6.60E-03	Metabolism	Carbohydrate Metabolism	Glycolysis / Gluconeogenesis	K00382 - dihydrolipoamide dehydrogenase dld, pdhD, lpd
1.066	4.28E-03	Unclassified	Unclassified: signaling and cellular processes	Transport	K03316 - monovalent cation:H+ antiporter, CPA1 family, TC.CPA1
0.873	6.78E-04	Metabolism	Glycan biosynthesis and metabolism	Peptidoglycan biosynthesis	K18149 - penicillin-binding protein pbp3, pbp4, pbp5
0.839	6.60E-03	Environmental Information Processing	Membrane Transport	ABC transporters	K02072 - D-methionine transport system permease protein metI
0.831	2.38E-02	Unclassified	-	-	K07139 - uncharacterized protein
0.790	3.99E-03	Metabolism	Amino Acid Metabolism	Phenylalanine, tyrosine and tryptophan biosynthesis	K04517 - prephenate dehydrogenase tyrA2 [EC:1.3.1.12]
0.761	3.69E-02	Unclassified	Protein families: signaling and cellular processes	Transporters	K05020 - glycine betaine transporter opuD, betL
0.700	2.82E-02	Metabolism	Nucleotide Metabolism	Purine metabolism	K01486 - adenine deaminase ade [EC:3.5.4.2]
0.669	3.30E-02	Unclassified	Protein families: genetic information processing	Transfer RNA biogenesis	K03177 - tRNA pseudouridine55 synthase, truB, PUS4, TRUB1
0.656	3.81E-02	Metabolism	Amino Acid Metabolism	Phenylalanine, tyrosine and tryptophan biosynthesis	K01735 - 3-dehydroquinate synthase aroB [EC:4.2.3.4]
-0.735	1.32E-02	Metabolism	Carbohydrate Metabolism	Pyruvate metabolism	K01759 - lactoylglutathione lyase glo1, gloA[EC:4.4.1.5]
-0.802	9.43E-03	Environmental Information Processing	Membrane Transport	Phosphotransferase system (PTS)	K02760 - PTS system, cellobiose-specific IIB component [EC:2.7.1.69], PTS-Cel-EIIB, celA, chbB
-0.811	4.92E-02	Metabolism	Carbohydrate Metabolism	Glycolysis / Gluconeogenesis	K04041 - fructose-1,6-bisphosphatase III fbp3 [EC:3.1.3.11]
-0.828	3.69E-02	Unclassified	Protein families: signaling and cellular processes	Transporters	K03284 - magnesium transporter corA
-0.846	1.87E-05	Unclassified	-	-	K07023 - putative hydrolases of HD superfamily
-0.849	3.71E-02	Unclassified	-	-	K05937 - uncharacterized protein
-0.910	3.25E-02	Metabolism	Carbohydrate Metabolism	Butanoate metabolism	K01575 - acetolactate decarboxylase [EC:4.1.1.5], budA, alsD, aldC

-0.912	2.73E-02	Unclassified	Protein families: metabolism	Peptidoglycan biosynthesis and degradation proteins	K01448 - N-acetylmuramoyl-L-alanine amidase amiABC
-1.115	3.99E-03	Unclassified	Protein families: signaling and cellular processes	Transporters	K03282 - large conductance mechanosensitive channel mscL
-1.147	3.25E-02	Environmental Information Processing	Membrane Transport	ABC transporters	K09693 - teichoic acid transport system ATP-binding protein tagH [EC:3.6.3.40]
-1.317	1.70E-02	Unclassified	Unclassified: metabolism	Enzymes with EC numbers	K00537 - arsenate reductase (glutaredoxin) arsC
-1.353	3.81E-02	Environmental Information Processing	Signal Transduction	Two-component system	K07706 - two-component system, LytTR family, sensor histidine kinase AgrC
-1.581	4.80E-02	Metabolism	Lipid Metabolism	Arachidonic acid metabolism	K00432 - glutathione peroxidase gpx [EC:1.11.1.9]
-1.772	2.94E-02	Environmental Information Processing	Signal Transduction	Two-component system	K07813 - accessory gene regulator B, argB
-1.903	7.52E-03	Genetic Information Processing	Translation	Ribosome	K02914 - large subunit ribosomal protein L34
-1.956	3.16E-04	Metabolism	Carbohydrate Metabolism	Glycolysis / Gluconeogenesis	K04072 - acetaldehyde dehydrogenase / alcohol dehydrogenase adhE [EC:1.2.1.10 1.1.1.1]
